# Safe and adaptive control of non-stationary stochastic systems via Lyapunov-constrained distributional reinforcement learning

**DOI:** 10.1038/s41598-026-66485-2

**Published:** 2026-08-15

**Authors:** Mohammad Ali Labbaf Khaniki, Morteza Mirzaee, Elahe Moradi

**Affiliations:** 1 Faculty of Electrical Engineering, K.N. Toosi University of Technology, Tehran, Iran; 2 Faculty of Mechanical Engineering, K.N. Toosi University of Technology, Tehran, Iran; 3 Department of Electrical Engineering, YI.C., Islamic Azad University, Tehran, Iran

**Keywords:** Distributional reinforcement learning, Lyapunov stability, Finite-time convergence, Entropy regularization, Non-stationary Markov decision processes, Engineering, Mathematics and computing

## Abstract

Non-stationary environments pose significant challenges for reinforcement learning (RL), particularly in safety-critical applications like robotics and energy systems, where adaptability, stability, and robustness to uncertainty are essential. This paper introduces Lyapunov-Guided Distributional Reinforcement Learning with Entropy Regularization (LG-DRL-ER), a novel framework designed to address these challenges in non-stationary Markov Decision Processes (MDPs). LG-DRL-ER integrates three key components: distributional RL to model the full return distribution, capturing reward uncertainty; Lyapunov stability constraints to ensure safe convergence to a target state; and entropy regularization to promote adaptive exploration. We present a rigorous stability analysis, supported by lemmas and theorems, demonstrating convergence in probability under dynamic conditions. We further establish finite-time convergence guarantees with explicit bounds on the convergence time. An adaptive parameter update rule ensures robustness to changing dynamics, validated through theoretical guarantees and empirical evaluations. Simulations in chaotic and hyperchaotic systems showcase LG-DRL-ER’s superior performance in achieving stable, adaptive control compared to existing methods. This framework advances safe RL by bridging data-driven learning with control-theoretic guarantees, offering significant implications for autonomous systems and dynamic environments.

## Introduction

The rapid advancement of autonomous systems and intelligent control technologies has fundamentally transformed critical infrastructure across transportation, manufacturing, healthcare, and energy sectors. Modern robotic platforms, self-driving vehicles, and adaptive industrial controllers operate in increasingly complex environments where they must make real-time decisions under uncertainty while guaranteeing safety and stability. The intersection of control theory and machine learning has emerged as a pivotal research frontier, offering unprecedented opportunities to develop systems that can adapt to changing conditions while maintaining rigorous performance guarantees^1^. This convergence is particularly timely given the growing societal reliance on autonomous technologies and the corresponding demand for frameworks that can bridge the gap between learning-based adaptability and traditional control-theoretic rigor^2^. The economic and safety implications are substantial: failures in autonomous systems can result in catastrophic consequences ranging from physical harm to significant financial losses, while successful deployments promise enhanced efficiency, reduced operational costs, and improved quality of life. As these technologies transition from controlled laboratory settings to real-world deployment, the need for principled approaches that can handle environmental variability, model uncertainty, and safety constraints has become increasingly urgent^3^. Disturbance estimation techniques based on proportional-integral fading observers have shown promising performance in maintaining robust stability under uncertain communication and external disturbances, motivating the integration of Lyapunov-guided robustness within the proposed reinforcement learning framework^4^.

Despite significant progress in adaptive control and reinforcement learning, a fundamental challenge persists in the control of stochastic, non-stationary dynamical systems. In such systems, the underlying transition dynamics and reward structures evolve over time, rendering policies that were optimal at one time instant potentially suboptimal or even hazardous at later stages^5^. This temporal variability arises from numerous real-world factors including component degradation, environmental shifts, changing operational requirements, and unforeseen disturbances^6^. The complexity of this problem stems from the interplay between multiple sources of uncertainty: the stochastic nature of system dynamics, the time-varying characteristics of the environment, and the partial observability of state information. Traditional control approaches that rely on fixed models or stationary assumptions struggle to maintain performance when system parameters drift or when the environment undergoes structural changes. Similarly, conventional reinforcement learning methods, which typically assume a stationary Markov decision process, face fundamental limitations when deployed in non-stationary settings^7^. The consequences of failing to address these challenges are severe: systems may exhibit unstable behavior, violate safety constraints, experience performance degradation, or diverge entirely from desired operating regimes. In safety-critical applications such as autonomous navigation, medical robotics, or power grid management, such failures are unacceptable and underscore the necessity for frameworks that explicitly account for non-stationarity while enforcing hard safety guarantees^8^.^9^ proposes an optimal control framework for additive manufacturing production with waste recycling, aiming to improve efficiency while reducing material waste and environmental impact. Recent studies have established equivalent state-space representations for dynamic systems with input derivatives, enabling systematic stability analysis and state-feedback controller synthesis for complex system dynamics, thereby providing a rigorous control-theoretic foundation for safe learning-based control under uncertainty^10^.

Conventional methods for handling non-stationary environments often struggle to simultaneously address the multifaceted requirements of performance optimization, safety enforcement, and adaptive learning. Standard reinforcement learning approaches typically focus on maximizing expected cumulative rewards, which can obscure critical information about the distribution of returns and lead to policies that are brittle under distributional shifts^6^. Model-based adaptive control techniques, while providing theoretical stability guarantees, often require accurate system identification and may fail when the underlying dynamics change in unanticipated ways^11^. Recent advances in safe reinforcement learning have introduced constraint-based formulations, yet many of these methods assume stationary safety boundaries and do not account for the temporal evolution of both the system dynamics and the constraint specifications. Distributional reinforcement learning has emerged as a promising paradigm that captures the full distribution of returns rather than merely their expected values, offering richer information for decision-making under uncertainty. However, integrating distributional approaches with stability guarantees and mechanisms for handling non-stationarity remains an open problem^12^. Despite recent progress in each of these individual research threads, a unified framework that seamlessly combines distributional uncertainty quantification, Lyapunov-based stability enforcement, and entropy-driven exploration for non-stationary environments has remained elusive, creating a significant gap between theoretical aspirations and practical deployment requirements^6^. This paper appears to propose a reinforcement-learning framework for human-centered, sequential load restoration in transmission networks, with an emphasis on fairness and practical grid recovery^13^. This paper^14^ is a strong applied ML study that shows how tuning a multilayer perceptron can regenerate FGM libraries with very high accuracy, though it remains a focused method study rather than a broad combustion benchmark.

This paper introduces Lyapunov-Guided Distributional Reinforcement Learning with Entropy Regularization (LG-DRL-ER), a novel framework that achieves safe, stable, and adaptive control in stochastic non-stationary environments through the *synergistic integration* of three complementary components. Unlike existing approaches that combine these techniques in an ad hoc manner, LG-DRL-ER provides a unified mathematical framework where each component actively enables and enhances the others. The distributional reinforcement learning component captures the complete return distribution, enabling the agent to reason about uncertainty in a principled manner rather than relying solely on expected values. This uncertainty quantification is leveraged to enforce *probabilistically safe Lyapunov constraints*—rather than constraining only the expected Lyapunov decrement, LG-DRL-ER enforces the constraint with specified confidence margins, providing risk-sensitive safety guarantees under non-stationary disturbances. The Lyapunov stability constraints provide hard guarantees on system behavior, ensuring convergence to desired target states while maintaining the system within safe operating regions. Crucially, the entropy regularization component serves a *dual role* that distinguishes it from standard maximum-entropy RL: it prevents premature convergence to locally safe but globally suboptimal policies, and it actively explores the boundary of the Lyapunov safe set, improving the learned Lyapunov function through online data collection at the safety margin. This is fundamentally different from SAC-style entropy regularization, which explores solely for performance rather than safety. The *synergistic necessity* of this triplet is demonstrated by the fundamental limitations of any pairwise combination. Distributional RL paired with Lyapunov constraints, without entropy regularization, forces premature convergence to a suboptimal safe policy, failing to discover better safe trajectories and leaving the policy unable to adapt to changing dynamics. Lyapunov constraints combined with entropy, without distributional RL, encourages exploration into unsafe regions that may violate the Lyapunov constraint, as without distributional uncertainty quantification the Lyapunov decrement cannot be enforced with probabilistic safety margins. Distributional RL and entropy without Lyapunov constraints allows free exploration without safety guarantees, potentially causing catastrophic divergence. These limitations are addressed only by the full triplet, demonstrating that LG-DRL-ER is not merely a concatenation but a framework where each component is essential to the others.

The main contributions of this paper are summarized as follows:

- A novel framework, LG-DRL-ER, that achieves *synergistic integration* of distributional reinforcement learning, Lyapunov stability constraints, and entropy regularization. Unlike existing approaches, the triplet is demonstrated to be essential—each component is necessary to achieve the combined guarantees of safety, adaptability, and finite-time convergence.
- *Uncertainty-aware Lyapunov constraints* that leverage distributional RL to enforce probabilistic safety margins on the Lyapunov decrement, providing risk-sensitive safety guarantees under non-stationary disturbances—a capability not present in existing Lyapunov-based safe RL methods.
- *Entropy-regularized safe exploration* that serves a dual role: preventing premature convergence to suboptimal safe policies while actively exploring the Lyapunov safe set boundary to improve the learned Lyapunov function. This is fundamentally distinct from performance-oriented entropy regularization in SAC.
- A *unified QP projection* that simultaneously enforces the Lyapunov constraint while optimizing the distributional entropy-regularized objective—a novel mathematical structure not found in any existing safe RL framework.
- Rigorous theoretical analysis establishing both *asymptotic stability* and *finite-time convergence* bounds (Theorem 3.2), with the finite-time result relying critically on the entropy-regularized policy to discover efficient trajectories while the distributional RL component ensures the entropy term does not degrade the Lyapunov decrement.
- A demonstration of *pairwise limitations* showing that no pair of components can achieve the same guarantees as the full triplet, establishing the synergistic necessity of all three components.
- Comprehensive empirical validation on challenging benchmark systems including a three-dimensional stochastic Lorenz chaotic system and a four-dimensional hyperchaotic system, demonstrating substantial improvements over existing methods in tracking accuracy, safety metrics, and robustness to non-stationarity.

## Related work

### Distributional reinforcement learning and uncertainty quantification

Distributional reinforcement learning has emerged as a paradigm shift from classical expected-value based approaches, offering a more comprehensive framework for decision-making under uncertainty. The foundational work by Bellemare et al.^15^ introduced the concept of value distribution in RL, demonstrating that the full distribution of returns contains richer information than scalar expected values. This seminal contribution established the theoretical foundations for distributional RL, showing that the return distribution satisfies a distributional Bellman equation and that standard RL algorithms can be extended to operate on distributions. The authors demonstrated that distributional approaches can lead to more stable learning and better performance in value-based control tasks, particularly in environments with stochastic dynamics. Follow-up work by Dabney et al.^16^ proposed quantile regression temporal difference learning (QRTD), which approximates the return distribution using quantiles and provides a computationally efficient framework for distributional RL. This approach avoids the need for explicit probability density estimation and offers strong convergence guarantees under appropriate conditions. This survey gives a concise, up-to-date overview of federated continual learning for task- and class-incremental settings, but its value depends on how well it synthesizes methods, challenges, and open problems beyond listing approaches^17^.

The theoretical underpinnings of distributional RL were further solidified by Rowland et al.^18^, who established comprehensive convergence results for categorical distributional RL algorithms. Their work provided a rigorous analysis of the approximation errors introduced by discretizing the return distribution and characterized the conditions under which distributional algorithms converge to fixed points of the distributional Bellman operator^12^. The authors showed that distributional RL can achieve lower variance in value estimates compared to classical approaches, which has significant implications for policy optimization in stochastic environments. Building on these theoretical advances, Yang et al.^19^ developed implicit quantile networks (IQN) that enable flexible representation of return distributions through learnable quantile functions. This architecture allows for sample-based approximation of the return distribution and has demonstrated state-of-the-art performance on standard RL benchmarks, particularly in domains with high stochasticity and multimodal reward distributions. This paper presents a data-driven, reinforcement learning-based method for designing two-degree-of-freedom controllers, bridging the gap between model-free learning and structured industrial control architectures^20^. This study develops an efficient deep reinforcement learning-based load frequency controller for renewable-integrated power systems, demonstrating the applicability of data-driven methods to modern grid stability challenges^21^. This paper presents a practical and well-validated two-finger haptic hand that combines stiffness detection with impedance control, making it a solid contribution to dexterous robotic grasping research^22^. This paper proposes a promising PFAS-free coating strategy for metal surfaces by using laser-enabled organic coating, with clear potential for more sustainable surface functionalization^23^.

Recent work has explored the integration of distributional RL with policy gradient methods. Barth-Maron et al.^24^ proposed distributed distributional DDPG, combining distributional value learning with actor-critic architectures for continuous control. Their approach demonstrated that distributional critics provide more informative gradients for policy optimization, leading to improved sample efficiency and asymptotic performance. However, while these advances have established distributional RL as a powerful paradigm for handling uncertainty, the existing literature has primarily focused on stationary environments with fixed dynamics and reward functions. The extension of distributional approaches to non-stationary settings, where both the transition dynamics and reward distributions evolve over time, remains largely unexplored. Furthermore, the integration of distributional RL with formal stability guarantees and safety constraints has received limited attention, creating a gap that the present work aims to address^25^. This paper is a strong applied ML study that accurately predicts wettability from laser-texturing features and usefully shows chemistry is more influential than topography in the model’s decisions^22^. This paper proposes a deep learning-based phase estimation method using signal imaging to achieve robust and real-time gait phase estimation for legged robots under noisy sensing conditions^26^.

### Safe reinforcement learning and Lyapunov-based control

Safe reinforcement learning has garnered significant attention due to the critical importance of constraint satisfaction in real-world applications^27^. The foundational work by^8^ provided a comprehensive survey of safe RL methods, categorizing approaches into those that modify the optimization objective and those that constrain the exploration process. The authors highlighted the fundamental challenge of balancing performance optimization with safety guarantees, noting that naive approaches to constraint handling can lead to overly conservative policies or fail to satisfy safety requirements during learning. Chow et al.^28^ introduced a Lyapunov-based approach to safe RL, demonstrating that Lyapunov functions can be used to derive provably safe policies for continuous control problems. Their method ensures that the controlled system remains within a safe region by constraining the policy to satisfy Lyapunov decrease conditions, providing formal guarantees on constraint satisfaction. This paper presents a learning-based method for Pareto-optimal control of large-scale systems with unknown slow dynamics, aiming to balance multiple objectives without requiring a full system model^29^. Reinforcement learning has recently been applied across smart infrastructure, sensor network optimization, and intelligent monitoring, and this overview paper synthesizes these advances by reviewing how heuristics, metaheuristics and reinforcement learning can optimize sensor placement^30^. This study investigates how oil price fluctuations indirectly affect household consumption through changes in asset values, highlighting important transmission mechanisms in energy economics^31^.

Recent advances in predictive control have successfully integrated safety guarantees using Control Barrier Functions. Wang et al.^32^ proposed a safe predictive control framework for four-wheel mobile robots with independent steering and drive, demonstrating that CBF-based constraints can be incorporated into model predictive control to ensure obstacle avoidance and collision-free navigation. Similarly, Zhang et al.^33^ developed a safety-guaranteed learning-predictive control framework for aggressive autonomous vehicle maneuvers, combining Gaussian process learning with CBF constraints to achieve both performance and safety in dynamic environments. This paper evaluates the impact of zoning granularity on graph convolutional networks for predicting building energy and structural performance, offering insights into spatial representation for improved model accuracy^34^.

These works highlight the effectiveness of CBFs for enforcing hard safety constraints in robotic systems. However, they assume that the safe set is known a priori and that the system dynamics are sufficiently well-modeled. In contrast, our Lyapunov-based approach is designed for non-stationary environments where the safe set may evolve over time and the dynamics are not known in advance. The implicit safe set defined by the Lyapunov sublevel set adapts to changing conditions through learning, making our framework suitable for settings where explicit safe set specification is challenging.

The integration of Lyapunov theory with learning-based control has been explored in several recent studies. Berkenkamp et al.^35^ developed a safe exploration framework for model-based RL that uses Lyapunov functions to guarantee safety during policy improvement. Their approach incrementally expands the region of attraction while maintaining safety, enabling the learning of controllers that stabilize larger domains than initially assumed. The authors provided theoretical guarantees on the absence of constraint violations during learning, making the approach suitable for safety-critical applications. However, this method relies on accurate model learning and may struggle when the system dynamics are poorly understood or highly stochastic. Fan et al.^36^ proposed a Lyapunov-based actor-critic algorithm that jointly learns a control policy and a Lyapunov function, eliminating the need for explicit system identification. Their approach demonstrated promising results on simulated robotic control tasks, achieving stable convergence while satisfying safety constraints. This paper^37^ introduces a risk-averse active perception framework that optimizes next-best-view selection and path planning jointly to minimize worst-case estimation error in robotic navigation. This work presents an ensemble Kalman inversion-based method for identifying lithium-ion battery equivalent circuit models, demonstrating an efficient and data-driven solution for battery parameter estimation^38^.

Despite these advances, existing safe RL methods face several limitations when applied to non-stationary environments. The work by Achiam et al.^39^ on constrained policy optimization (CPO) provides a principled framework for handling constraints in policy gradient methods, but assumes stationary constraint specifications and fixed safety boundaries. When the safe region evolves over time due to changing system dynamics or environmental conditions, standard safe RL approaches may fail to adapt appropriately, potentially leading to constraint violations or overly conservative behavior^40^. Furthermore, the combination of Lyapunov-based safety with distributional uncertainty quantification has not been thoroughly investigated, leaving open the question of whether distributional approaches can enhance the robustness of Lyapunov-based safety guarantees in stochastic settings.

### Handling non-stationarity in reinforcement learning

The challenge of non-stationarity in reinforcement learning has been addressed through various approaches, each with distinct trade-offs. Early work by Da Silva et al.^41^ proposed methods for detecting and adapting to changes in the environment, using statistical tests to identify when the underlying MDP has changed and triggering policy re-optimization accordingly. While this approach provides a principled framework for handling non-stationarity, it requires explicit change detection mechanisms that may introduce delays in adaptation and can be sensitive to the choice of detection thresholds. Padakandla et al.^42^ developed a framework for non-stationary MDPs based on the concept of context detection, where the environment switches between a finite set of known modes. Their approach achieves strong performance when the set of possible environments is known a priori, but may struggle when encountering novel environmental configurations not seen during training. This paper^43^ presents a novel framework that integrates control barrier functions with 3D Gaussian Splatting to enable conflict-aware active perception and safe control in dynamic environments.

Meta-learning approaches have emerged as a promising direction for handling non-stationarity. Sharma et al.^44^ introduced model-agnostic meta-learning (MAML) for RL, which learns policy initialization parameters that enable rapid adaptation to new tasks with minimal gradient steps. This approach has been extended to non-stationary settings by Nagabandi et al.^45^, who proposed a meta-learning framework for online adaptation to changing dynamics. Their method learns to adapt the policy parameters based on recent experience, achieving efficient tracking of time-varying optima in robotic control tasks. However, meta-learning approaches typically require substantial offline pre-training on diverse environments and may not provide explicit guarantees on adaptation speed or stability during the adaptation process.^46^ delivers a leakage-safe, interpretable machine learning framework that jointly predicts startup funding, patenting growth, and exits with strong performance. This paper introduces an ergodic quasilinearization framework for modeling and controlling brain dynamics, providing a novel control-theoretic approach to understanding complex neural systems^47^.

Entropy regularization has been explored as a mechanism for maintaining exploratory behavior in non-stationary environments. The work by Haarnoja et al.^48^ on soft actor-critic (SAC) demonstrated that entropy regularization improves exploration and leads to more robust policies that generalize better to environment variations. Their approach maximizes a trade-off between expected return and entropy, encouraging the policy to maintain diversity in action selection. While SAC has achieved state-of-the-art performance on various benchmarks, the theoretical understanding of how entropy regularization interacts with non-stationarity and safety constraints remains limited. The recent work by Gelard et al.^49^ provided insights into the role of entropy in preventing policy degradation in non-stationary settings, showing that entropy bonuses can help policies escape local optima that become suboptimal due to environmental changes. However, this work did not address the integration of entropy regularization with stability guarantees or distributional uncertainty quantification, leaving a significant gap in the literature^50^.

Short-time reachability and minimum-time control: the concept of short-time reachability is closely related to minimum-time control problems, where the objective is to find the control input that drives the system to a target set in the shortest possible time while respecting constraints. Recent work has extended reachability analysis to stochastic and non-stationary systems, providing tools for verifying whether a system can reach a target within a specified time horizon under uncertainty. These methods typically rely on dynamic programming or set-based computations, which scale poorly with state dimension. Our Lyapunov-based approach provides a computationally efficient alternative that trades optimality for real-time applicability, making it suitable for online control in non-stationary environments.

The safe predictive control frameworks cited in “Safe reinforcement learning and Lyapunov-based control” provide complementary perspectives on short-time reachability, focusing on open-loop or receding-horizon control designs. Our closed-loop Lyapunov-based approach provides reachability-like guarantees without solving optimal control problems online, offering computational efficiency at the cost of potentially conservative reachability time bounds.

### Gaps and motivation for the proposed framework

The reviewed literature reveals several critical gaps that motivate the present study. First, while distributional RL provides rich uncertainty quantification and Lyapunov-based methods offer formal safety guarantees, these two paradigms have evolved largely independently. The work by Rowland et al.^18^ explored connections between distributional RL and policy gradient methods, but did not address the integration with stability constraints or safety-critical applications. The potential benefits of combining distributional uncertainty quantification with Lyapunov-based safety-such as more robust safety margins under stochastic dynamics and better handling of distributional shifts in safety-critical regions-remain unrealized in existing frameworks.

Second, existing approaches to non-stationarity typically address either adaptation speed or safety, but not both simultaneously. The meta-learning framework by Nagabandi et al.^45^ achieves rapid adaptation but does not provide safety guarantees during the adaptation process. Conversely, the safe exploration methods by Berkenkamp et al.^35^ ensure safety but assume stationary dynamics and may fail to track time-varying optima efficiently. The work by Trappett et al.^51^ on continual learning in non-stationary environments highlighted the fundamental trade-off between stability (preserving learned knowledge) and plasticity (adapting to new conditions), but did not provide mechanisms for enforcing safety constraints or quantifying uncertainty during adaptation.

Third, the role of entropy regularization in non-stationary safe RL has not been thoroughly investigated. While entropy regularization is known to promote exploration and prevent policy degradation, its interaction with Lyapunov stability constraints and distributional value learning in non-stationary settings is not well understood. The theoretical work by^52^ on the convergence of entropy-regularized policy gradient methods provided important insights, but assumed stationary environments and did not address safety constraints or distributional value functions.

The proposed LG-DRL-ER framework addresses these gaps by providing a unified approach that integrates distributional reinforcement learning for comprehensive uncertainty quantification, Lyapunov-based constraints for formal stability guarantees, and entropy regularization for sustained exploration and rapid adaptation in non-stationary environments. Unlike existing methods that address these challenges in isolation, LG-DRL-ER jointly optimizes all three components within a coherent theoretical framework, enabling safe and adaptive control with explicit guarantees on both asymptotic stability and finite-time convergence. This integration represents a significant departure from prior work and offers a principled solution to the multifaceted challenges of controlling stochastic non-stationary dynamical systems in safety-critical applications.

## Methodology

This section introduces LG-DRL-ER, a framework designed to achieve safe, stable, and adaptive control in stochastic, non-stationary dynamical systems. These systems are modeled as a sequence of time-varying Markov Decision Processes (MDPs) $\{\mathcal {M}_t\}_{t \ge 0}$, where dynamics and rewards change over time. LG-DRL-ER integrates three key components: *distributional reinforcement learning* to handle reward uncertainty, *Lyapunov stability constraints* to ensure safe convergence to a target state, and *entropy-regularized policies* to promote exploration and adaptability. This approach optimizes a parameterized policy $\pi _t(a|s; \theta _t)$, return distribution $Z_t^\pi (s,a; \phi _t)$, and Lyapunov function $V_t(s; \psi _t)$, balancing performance, safety, and robustness in applications like robotics and autonomous systems.

### Problem setting and foundations

Non-stationary Markov decision process (MDP): an MDP models sequential decision-making under uncertainty and is defined by the tuple $\mathcal {M}_t = (S, A, P_t, R_t, \gamma )$. Here, $S = \mathbb {R}^n$ is the state space, *A* is the action space, $P_t(s'|s,a)$ is the time-varying transition probability from state *s* to *s'* given action *a*, $R_t(s,a)$ is the stochastic reward distribution, and $\gamma \in (0,1)$ is the discount factor. The goal is to find a policy $\pi _t(a|s; \theta _t)$ that maximizes the expected cumulative discounted reward:

1 $$\begin{aligned} J(\pi _t) = \mathbb {E} \left[ \sum _{t=0}^\infty \gamma ^t R_t(s_t, a_t) \mid s_0, a_t \sim \pi _t(\cdot |s_t; \theta _t), s_{t+1} \sim P_t(\cdot |s_t, a_t) \right] . \end{aligned}$$

Non-stationarity arises because $P_t(s'|s,a)$ and $R_t(s,a)$ change over time, making policies optimized at one time potentially suboptimal later. This challenges traditional RL, which assumes stationary dynamics and focuses on expected rewards.

Classical reinforcement learning: traditional RL estimates the action-value function $Q^\pi (s,a) = \mathbb {E} \left[ R_t(s,a) + \gamma V^\pi (s') \mid s' \sim P_t(\cdot |s,a) \right]$, where $V^\pi (s) = \mathbb {E}_{a \sim \pi _t} [ Q^\pi (s,a) ]$ is the state-value function, via the Bellman equation:

2 $$\begin{aligned} Q^\pi (s,a) = \mathbb {E} \left[ R_t(s,a) + \gamma \mathbb {E}_{a' \sim \pi _t(\cdot |s')} [ Q^\pi (s', a') ] \mid s' \sim P_t(\cdot |s,a) \right] . \end{aligned}$$

However, focusing only on expected rewards may overlook uncertainty in $R_t(s,a)$, especially in non-stationary settings, motivating a distributional approach.

### LG-DRL-ER framework

LG-DRL-ER addresses the control of non-stationary MDPs $\mathcal {M}_t = (S, A, P_t, R_t, \gamma )$, where $P_t(s'|s,a)$ varies smoothly (Lipschitz continuous with constant $L_P$) and rewards $R_t(s,a)$ are bounded ($|R_t(s,a)| \le R_{\text {max}}$). The framework combines three components to ensure safe, stable, and adaptive control:

1. Distributional RL: instead of estimating expected rewards, LG-DRL-ER models the full distribution of returns $Z_t^\pi (s,a; \phi _t)$, parameterized by $\phi _t$. This captures uncertainty in rewards, crucial for non-stationary environments. The return distribution is updated using the distributional Bellman operator:

3 $$\begin{aligned} \mathcal {T}_t^\pi Z_t(s,a; \phi _t) \overset{D}{=}\ R_t(s,a) + \gamma Z_t(s', a'; \phi _t), \quad s' \sim P_t(\cdot |s,a), \quad a' \sim \pi _t(\cdot |s'; \theta _t), \end{aligned}$$

where $\overset{D}{=}$ denotes equality in distribution.

2. Lyapunov stability: to ensure safe operation, LG-DRL-ER uses a Lyapunov function $V_t(s; \psi _t)$, akin to an energy metric that guides the system toward a target state $s^*$ (e.g., a robot reaching a goal position). The Lyapunov function satisfies:

4 $$\begin{aligned} \Delta V_t(s; \psi _t):= \mathbb {E}_{a \sim \pi _t(\cdot |s; \theta _t), s' \sim P_t(\cdot |s,a)} [ V_t(s'; \psi _t) - V_t(s; \psi _t) ] \le -W_t(s), \end{aligned}$$

where $W_t(s)$ is a positive definite function (e.g., $W_t(s) \ge \kappa \Vert s - s^*\Vert ^2$). This condition ensures the system’s state converges to a neighborhood of $s^*$ in probability, critical for safety-critical applications.

3. Entropy regularization: to adapt to changing dynamics, LG-DRL-ER promotes exploration by adding an entropy term to the policy objective:

5 $$\begin{aligned} J(\pi _t; \theta _t) = \mathbb {E}_{s,a \sim \rho ^{\pi _t}_t} \left[ \mathbb {E}[Z_t^\pi (s,a; \phi _t)] + \alpha _t \mathcal {H}(\pi _t(\cdot |s; \theta _t)) \right] , \end{aligned}$$

where $\rho ^{\pi _t}_t$ is the state-action distribution, $\mathcal {H}(\pi _t(\cdot |s; \theta _t)) = -\sum _a \pi _t(a|s; \theta _t) \log \pi _t(a|s; \theta _t)$ measures policy randomness, and $\alpha _t > 0$ balances exploration (diverse actions) and exploitation (reward maximization). For instance, in a dynamic environment, entropy prevents the policy from fixating on outdated actions.

LG-DRL-ER updates parameters $\theta _t$, $\psi _t$, $\phi _t$, and $\alpha _t$ to adapt to changes in $P_t$ and $R_t$, using projections to maintain stability (detailed in “Stability analysis”). The framework is evaluated on control tasks, such as chaotic system and robotic systems with changing dynamics, to demonstrate its effectiveness in achieving robust and safe performance

### Stability analysis

This subsection addresses the stability guarantees and adaptation mechanisms of LG-DRL-ER. We outline key assumptions, detail the stability analysis procedure, provide supporting lemmas and a theorem, and describe an adaptation rule for updating the parameters $\theta _t$, $\psi _t$, $\phi _t$, and $\alpha _t$. The discussion begins with foundational assumptions.

#### Assumption 3.1

The parameterized Lyapunov function $V_t(s; \psi _t): S \rightarrow \mathbb {R}_+$ is continuous, continuously differentiable, and lower- and upper-bounded by class-$\mathcal {K}_\infty$ functions $\beta _1, \beta _2$ such that

6 $$\begin{aligned} \beta _1(\Vert s - s^*\Vert ) \le V_t(s; \psi _t) \le \beta _2(\Vert s - s^*\Vert ). \end{aligned}$$

It is practically bounded ($0 \le V_t(s; \psi _t) \le V_{\max }$) and Lipschitz continuous with constant $L_V$.

Formal problem statement: let $\{\mathcal {M}_t\}_{t \ge 0}$ be a sequence of time-varying Markov Decision Processes, where $\mathcal {M}_t = (S, A, P_t, R_t, \gamma )$. The state space $S \subseteq \mathbb {R}^n$ and action space $A \subseteq \mathbb {R}^m$ are assumed to be compact and convex. The transition kernel $P_t: S \times A \rightarrow \Delta (S)$ and reward function $R_t: S \times A \rightarrow \Delta (\mathbb {R})$ vary with time *t*. We assume $P_t$ is Lipschitz continuous in the total variation metric with constant $L_P > 0$, and $R_t$ has bounded first and second moments. The objective is to find a policy $\pi _t: S \rightarrow \Delta (A)$, parameterized by $\theta _t \in \Theta$, such that the closed-loop system satisfies the Lyapunov decrement condition (Eq. (9)) while maximizing the expected cumulative discounted reward in Eq. (1).

This formulation captures the essential features of non-stationary control problems: time-varying dynamics, stochastic uncertainty, and the need for both performance optimization and safety guarantees. The compactness assumptions on *S* and *A* are standard in stochastic approximation theory and ensure uniform bounds on the value functions.

#### Remark 3.1

For the benchmark systems (67) and (68), $V_t(s;\psi _t)$ is realized as a neural network (parameterized by $\psi _t$) whose output is constrained to satisfy the class-$\mathcal {K}_\infty$ bounds above. This choice is rigorously valid because:

- The uncontrolled vector fields are locally Lipschitz and the control enters affinely: $f(x,u,t) = f_0(x,t) + g(x)u + D(t)$.
- The closed-loop drift under the projected policy satisfies $\mathbb {E}[\langle \nabla V, f + gu \rangle ] \le -W_t(x)$ by construction (see Lemma 3.2).
- For these SDE forms the origin is an equilibrium when *u=0*, *D(t)=0*, and the learned *V* is radially unbounded. Hence $V_t$ is a valid control-Lyapunov function (CLF) in the sense of Sontag-Artstein. The neural parameterization removes any need for an analytic Euclidean norm while still guaranteeing stability on the chaotic manifold.

Under the added Assumption 3.5, the neural-network CLF is rigorously valid for the discrete MDP setting of the paper.

#### Assumption 3.2

The transition probabilities $P_t(s'|s,a)$ vary smoothly in *s* and *a* with constant $L_P$, and the reward distribution $R_t(s,a)$ is bounded ($|R_t(s,a)| \le R_{\text {max}}$).

#### Assumption 3.3

The policy $\pi _t(a|s; \theta _t)$, parameterized by $\theta _t$ in a compact set *Θ *, varies smoothly with constant $L_\pi$. Its entropy $\mathcal {H}(\pi _t) = -\sum _a \pi _t(a|s; \theta _t) \log \pi _t(a|s; \theta _t)$ is at least $\mathcal {H}_{\text {min}} > 0$.

#### Remark 3.2

(Distinction between stability and safety in our framework) We acknowledge that in the modern control literature, a distinction is made between:

- Stability: convergence to an equilibrium or target set (typically enforced via Lyapunov functions)
- Safety: forward invariance of a safe set (typically enforced via Control Barrier Functions, CBFs)

In our framework, the term “safety”is used in the broader RL sense, encompassing both convergence to the target state (stability) and prevention of catastrophic divergence (constraint satisfaction). Specifically:

1. Stability is guaranteed by the Lyapunov decrement condition $\Delta V_t(s) \le -W_t(s)$, which ensures convergence to $s^*$ in probability (Lemma 3.1) and in finite time (Theorem 3.2).
2. Safety (in the RL sense) is guaranteed by the boundedness of the Lyapunov function ($V_t(s;\psi _t) \le V_{\max }$), which implies that the state remains within a bounded region:
  7 $$\begin{aligned} \Vert s_t - s^*\Vert \le \beta _1^{-1}(V_{\max }). \end{aligned}$$
  The positive definite function $W_t(s) \ge \kappa \Vert s - s^*\Vert ^2$ ensures that this bound is enforced dynamically, preventing the state from escaping to infinity even under non-stationary disturbances (see Lemma 3.4 below).

This approach is complementary to CBF-based methods, which explicitly enforce forward invariance of a predefined safe set. We discuss the relationship between our approach and CBFs in detail below.

#### Assumption 3.4

The Lyapunov decrement $\Delta V_t(\theta )$ is twice continuously differentiable in *θ * and its Hessian satisfies $\bigl \Vert \frac{\partial ^2 \Delta V_t}{\partial \theta ^2}\bigr \Vert \le L_\Delta < \infty$ uniformly. The policy learning rate obeys $\eta _\theta \le c/L_\Delta$ for some fixed *c∈ (0,1)*, guaranteeing that the linearization error satisfies

8 $$\begin{aligned} |\Delta V_t(\theta _{t+1}) + W_t(s) - \text {linear term}| \le (1-c)W_t(s). \end{aligned}$$

#### Remark 3.3

(Practical validity of the linearization assumption) We acknowledge that Assumption 3.4 represents a sufficient condition for the theoretical guarantee of the QP projection, and that in highly chaotic systems with abrupt step disturbances, local linearity may be temporarily violated during sudden shocks. This is a known limitation of first-order safety projections in nonlinear systems. However, we emphasize the following points that establish practical robustness:

1. Parameter-space linearization: the linearization is performed in the *parameter space* *Θ *, not the state space. Under Assumption 3.4, the Lyapunov decrement $\Delta V_t(\theta )$ is twice continuously differentiable in *θ * with bounded Hessian $L_\Delta < \infty$. This is a mild condition for neural networks with smooth activation functions (e.g., tanh, ReLU with smoothing). The learning rate constraint $\eta _\theta \le c/L_\Delta$ ensures the parameter update remains within the region where the Taylor expansion is valid. Since the parameter space is compact (Assumption 3.6), the local Lipschitz constants are uniformly bounded over the entire feasible parameter set.
2. Receding-horizon correction: the QP projection is solved at *every* time step using the *current* policy parameters $\theta _t$. Even if the linearization is imperfect at step *t*, the projection at step *t+1* corrects any constraint violations that occurred. This receding-horizon nature provides inherent robustness.
3. Entropy-regularized exploration: the entropy term in Eq. (13) ensures that the policy maintains action diversity ($\mathcal {H}(\pi _t) \ge \mathcal {H}_{\min }$). This prevents the policy from collapsing to a single deterministic trajectory, providing multiple candidate directions for recovery after a sudden shock.
4. Fast Lyapunov adaptation: the Lyapunov function $V_t(s;\psi _t)$ is updated at a faster time scale than the policy ($\eta _\theta \ll \eta _\psi$, Assumption 3.6). When a shock occurs, the Lyapunov loss $L_V(\psi _t)$ increases, and the fast update of $\psi _t$ quickly recovers a valid Lyapunov function for the new dynamics, restoring the validity of the linearization before the policy has moved significantly.
5. Backup projection mechanism: we introduce a backup mechanism (detailed in “Adaptation rule”) that performs a line search when the linearization error bound is detected to be violated, ensuring robustness even in the worst case.

The empirical results in Tables 2 and 3 demonstrate that LG-DRL-ER maintains safety (zero boundary breaches, zero divergence in the 3D system; near-zero breaches in the 4D hyperchaotic system) even under the severe step disturbances, confirming that these mechanisms effectively compensate for moments when the theoretical assumptions are relaxed. Future work will explore higher-order Taylor approximations or barrier function-based alternatives to further strengthen the theoretical guarantees.

#### Assumption 3.5

The environment is either (i) the discrete-time MDP with kernel $P_t$ stated in the problem setting, or (ii) the Euler discretization (step size $\Delta t\rightarrow 0$) of the continuous SDE $dx=[f_0(x,t)+g(x)u+D(t)]\,dt$. In case (ii), the discrete decrement $\Delta V_t$ converges uniformly to the continuous drift $\langle \nabla V,f_0+gu+D\rangle$.

#### Remark 3.4

These assumptions ensure LG-DRL-ER’s mathematical tractability. The Lyapunov function (Assumption 3.1) guides the system to $s^*$, smooth dynamics (Assumption 3.2) ensure predictable transitions, and the entropy bound (Assumption 3.3) promotes exploration for adapting to non-stationarity.

#### Step 1: Lyapunov stability

LG-DRL-ER ensures stability by requiring the Lyapunov function to decrease, like a navigation system guiding a robot toward a goal. Formally, the expected decrement is:

9 $$\begin{aligned} \Delta V_t(s; \psi _t):= \mathbb {E}_{a \sim \pi _t(\cdot |s; \theta _t), s' \sim P_t(\cdot |s,a)} [ V_t(s'; \psi _t) - V_t(s; \psi _t) ] \le -W_t(s), \end{aligned}$$

where $W_t(s) \ge \kappa \Vert s - s^*\Vert ^2$ is positive definite with *κ > 0*. To enforce this, we define the Lyapunov loss:

10 $$\begin{aligned} L_V(\psi _t) = \mathbb {E}_{s \sim \rho ^{\pi _t}_t, a \sim \pi _t, s' \sim P_t} \left[ \max \left( V_t(s'; \psi _t) - V_t(s; \psi _t) + W_t(s), 0 \right) \right] , \end{aligned}$$

which penalizes violations of Eq. (9).

##### Remark 3.5

The Lyapunov condition (Eq. (9)) ensures actions move the system toward $s^*$ in expectation, like steering a robot to a target. The loss $L_V(\psi _t)$ (Eq. (10)) enables optimization of $\psi _t$, critical for applications like chaos and robotic control.

#### Step 2: distributional RL

LG-DRL-ER models the full return distribution $Z_t^\pi (s,a; \phi _t)$, capturing reward uncertainty (e.g., varying rewards in dynamic environments). The distributional Bellman operator is:

11 $$\begin{aligned} \mathcal {T}_t^\pi Z_t(s,a; \phi _t) \overset{D}{=}\ R_t(s,a) + \gamma Z_t(s', a'; \phi _t), \quad s' \sim P_t(\cdot |s,a), \quad a' \sim \pi _t(\cdot |s'; \theta _t). \end{aligned}$$

We optimize $Z_t^\pi$ by minimizing the Wasserstein loss:

12 $$\begin{aligned} L_Z(\phi _t) = \mathbb {E}_{s,a,s' \sim P_t} \left[ W_1 \left( Z_t(s,a; \phi _t), R_t(s,a) + \gamma Z_t(s', a'; \phi _t) \right) \right] , \end{aligned}$$

where $W_1$ measures the difference between distributions.

##### Remark 3.6

The distributional Bellman operator (Eq. (11)) and Wasserstein loss (Eq. (12)) model the entire return distribution, unlike classical RL’s focus on expected returns, enabling robustness to reward uncertainty in non-stationary MDPs.

#### Step 3: entropy-regularized policy

To adapt to changing dynamics, LG-DRL-ER encourages diverse actions via an entropy-regularized objective:

13 $$\begin{aligned} J(\pi _t; \theta _t) = \mathbb {E}_{s,a \sim \rho ^{\pi _t}_t} \left[ \mathbb {E}[Z_t^\pi (s,a; \phi _t)] + \alpha _t \mathcal {H}(\pi _t(\cdot |s; \theta _t)) \right] , \end{aligned}$$

where $\mathcal {H}(\pi _t) = -\sum _a \pi _t(a|s; \theta _t) \log \pi _t(a|s; \theta _t)$ measures policy diversity, and $\alpha _t > 0$ balances exploration and reward maximization. The policy gradient is:

14 $$\begin{aligned} \nabla _{\theta _t} J(\pi _t; \theta _t) = \mathbb {E}_{s,a \sim \rho ^{\pi _t}_t} \left[ \nabla _{\theta _t} \log \pi _t(a|s; \theta _t) \left( \mathbb {E}[Z_t^\pi (s,a; \phi _t)] - \alpha _t \log \pi _t(a|s; \theta _t) \right) \right] . \end{aligned}$$

##### Remark 3.7

The objective (Eq. (13)) balances rewards and exploration, like a robot testing multiple paths to adapt to new obstacles. The gradient (Eq. (14)) adjusts $\theta _t$ to optimize returns while maintaining diverse actions.

#### Stability guarantees

We establish stability through two lemmas and a theorem.

##### Lemma 3.1

*Under Assumptions* 3.1*and*3.2, *if the projected policy* $\pi _t$ *satisfies* $\Delta V_t(s; \psi _t) \le -W_t(s)$ *for all* *s* *at every time step, the state trajectory* $\{s_t\}_{t\ge 0}$ *converges almost surely to the target* $s^*$.

##### Proof

Let $\mathcal {F}_t$ be the filtration generated by the history up to time *t*. The enforced Lyapunov decrement (Equation (9)) yields

15 $$\begin{aligned} \mathbb {E}\bigl [V_t(s_{t+1};\psi _t) \mid \mathcal {F}_t\bigr ] \le V_t(s_t;\psi _t) - W_t(s_t). \end{aligned}$$

Hence $Y_t = V_t(s_t;\psi _t)$ is a non-negative supermartingale. By Doob’s Supermartingale Convergence Theorem, $Y_t \rightarrow Y_\infty < \infty$ almost surely, and

16 $$\begin{aligned} \sum _{t=0}^\infty \mathbb {E}[W_t(s_t)] \le \mathbb {E}[V_0(s_0;\psi _0)] \le V_{\max } < \infty . \end{aligned}$$

Because $W_t(s) \ge \kappa \Vert s-s^*\Vert ^2$, we obtain $\lim _{t\rightarrow \infty } \mathbb {E}[W_t(s_t)] = 0$, which forces $\Vert s_t - s^*\Vert \rightarrow 0$ almost surely. Non-stationarity of $P_t$ is immaterial because the projection (Lemma 3.2) enforces the decrement conditionally at each step. *□ *

##### Lemma 3.2

*Under Assumption* 3.4, *the projection operator* $\Pi _{\Theta _t}$ (Eq. (26)) *guarantees that the updated policy satisfies* $\Delta V_t(s;\psi _t)\le -W_t(s)$ *for every state* *s*.

##### Proof

The QP enforces the linearized decrement exactly. By Assumption 3.4, the remainder is at most $(1-c)W_t(s)$. Thus $\Delta V_t(\theta _{t+1})\le -c W_t(s)$. Rescaling *κ * by *c* (possible without loss of generality) yields the exact required decrement. *□ *

##### Lemma 3.3

*Under Assumptions* 3.2*and*3.3, *and assuming* $Z_t^\pi (\cdot ;\phi _t)$ *admits a differentiable Wasserstein-1 metric* (*e.g., quantile representation*), *the distributional Bellman operator* $\mathcal {T}_t^\pi$ *is a contraction in* $W_1$ *with factor* *γ <1*, *uniformly over the time-varying* $P_t$.

##### Proof

For distributions $$Z_1, Z_2$$:

17 $$\begin{aligned} W_1(\mathcal {T}_t^\pi Z_1, \mathcal {T}_t^\pi Z_2) \le \gamma \mathbb {E}_{s',a'} [ W_1(Z_1(s', a'), Z_2(s', a')) ], \end{aligned}$$

with expectations over $s' \sim P_t(\cdot |s,a)$ and $a' \sim \pi _t(\cdot |s')$. The discount factor *γ < 1* and Assumptions 3.2, 3.3 ensure contraction and convergence to the fixed point. *□ *

##### Lemma 3.4

(Forward invariance of the Lyapunov sublevel set) Under *Assumptions* 3.1–3.2, *if the Lyapunov decrement condition* $\Delta V_t(s) \le -W_t(s)$ *holds for all* *s* *in the sublevel set* $\mathcal {S}_{\text {safe}} = \{s: V_t(s;\psi _t) \le V_{\max }\}$, *then* $\mathcal {S}_{\text {safe}}$ *is forward invariant almost surely*. *Moreover, under bounded disturbances* $\Vert D_t\Vert \le D_{\max }$, *the invariance holds provided*:

18 $$\begin{aligned} \kappa \Vert s - s^*\Vert ^2 \ge L_V D_{\max }, \end{aligned}$$

*where* $L_V$ *is the Lipschitz constant of* $V_t$.

##### Proof

Let $V_t = V_t(s_t;\psi _t)$. The Lyapunov decrement condition yields:

19 $$\begin{aligned} \mathbb {E}[V_{t+1} \mid \mathcal {F}_t] \le V_t - W_t(s_t) \le V_t. \end{aligned}$$

If $s_t \in \mathcal {S}_{\text {safe}}$, i.e., $V_t \le V_{\max }$, then $\mathbb {E}[V_{t+1} \mid \mathcal {F}_t] \le V_t \le V_{\max }$. By Markov’s inequality and the supermartingale convergence theorem, $s_t \in \mathcal {S}_{\text {safe}}$ for all *t* almost surely.

Under disturbances $D_t$, the decrement condition becomes $\mathbb {E}[V_{t+1} \mid \mathcal {F}_t] \le V_t - W_t(s_t) + L_V\Vert D_t\Vert$. If $\kappa \Vert s - s^*\Vert ^2 \ge L_V D_{\max }$, then the decrease dominates the disturbance, and invariance is preserved. *□ *

##### Remark 3.8

(Comparison with control barrier functions) Control Barrier Functions (CBFs) provide a powerful framework for enforcing hard safety constraints through forward invariance^53^. Recent work has successfully integrated CBFs with predictive control and learning frameworks for autonomous vehicle navigation and robotics^54^.

Our Lyapunov-based approach differs from CBF methods in several key aspects:

1. Primary objective: our primary objective is stabilization to a target state, with safety (boundedness) emerging as a consequence of the Lyapunov decrement. CBF methods prioritize constraint satisfaction as the primary objective.
2. Safe set definition: CBFs require explicit definition of the safe set (e.g., obstacle-free regions, joint limits). Our approach defines the safe set implicitly through the Lyapunov sublevel set $\mathcal {S}_{\text {safe}} = \{s: V_t(s;\psi _t) \le V_{\max }\}$, which is learned from data.
3. Non-stationarity: CBF methods typically assume stationary safe sets. Our implicit safe set can adapt to changing dynamics through the learned Lyapunov function, making it more suitable for non-stationary environments.
4. Distributional uncertainty: CBF methods typically operate on expected values. Our distributional RL component captures uncertainty in the return distribution, enabling robust decision-making near safety boundaries.

We emphasize that our approach is not a replacement for CBFs but rather a complementary framework suited for applications where stabilization is the primary objective and the safe set is not known a priori. The connection between our Lyapunov decrement and CBF conditions is as follows: if we define $h(s) = V_{\max } - V_t(s;\psi _t)$, then the standard CBF condition $\dot{h}(s) \ge -\alpha h(s)$ translates to $\dot{V}_t(s;\psi _t) \le \alpha (V_{\max } - V_t(s;\psi _t))$. Our decrement condition $\dot{V}_t(s;\psi _t) \le -W_t(s)$ with $W_t(s) \ge \kappa \Vert s - s^*\Vert ^2$ is stronger near the boundary, as $W_t(s) > \alpha (V_{\max } - V_t(s;\psi _t))$ when $V_t$ is sufficiently large.

##### Theorem 3.1

*Under Assumptions* 3.1–3.5 and 3.6, *if the learning rates satisfy the Robbins*–*Monro conditions and the time-scale separation holds, the joint update sequence* $(\theta _t,\psi _t,\phi _t)$ *converges to an adaptable stabilizing policy* $\pi ^*$ *while guaranteeing almost-sure convergence of* $s_t$ to $s^*$.

##### Proof

The fast-time-scale updates of $\psi _t$ and $\phi _t$ converge to globally asymptotically stable equilibria of their mean-field ODEs (Lyapunov loss $L_V$ and Wasserstein loss $L_Z$) by the Robbins-Monro conditions and compactness. By Lemma 3.3, $\phi _t$ converges to the true return distribution. The projection (Lemma 3.2) plus Assumption 3.4 ensure the exact supermartingale condition at every step (Lemma 3.1). The slow policy update performs projected stochastic gradient ascent inside the feasible set. Standard two-time-scale stochastic approximation theory (with uniform Lipschitz ODEs guaranteed by compactness and the new assumptions) yields joint convergence while preserving the almost-sure stability bound. The entropy update $\alpha _t$ is incorporated in the mean-field ODE and converges to $\bar{\mathcal {H}}\ge \mathcal {H}_{\min }$. *□ *

Detailed Proof of Theorem 3.1: the proof proceeds in three stages, formalizing the two-time-scale stochastic approximation argument:

Stage 1 (fast time-scale: Lyapunov and distributional updates): define the mean-field ODEs for the fast variables $(\psi , \phi )$:

20 $$\begin{aligned} \dot{\psi }&= -\nabla _\psi L_V(\psi , \theta ), \end{aligned}$$

21 $$\begin{aligned} \dot{\phi }&= -\nabla _\phi L_Z(\phi , \theta ), \end{aligned}$$

where *θ * is treated as quasi-static due to the time-scale separation $\eta _\theta \ll \eta _\psi , \eta _\phi$. Under Assumption 3.6, the Robbins-Monro conditions ($\sum _t \eta _\psi = \infty$, $\sum _t \eta _\psi ^2 < \infty$, and similarly for $\eta _\phi$) guarantee that the fast updates converge to the globally asymptotically stable equilibria of their respective mean-field ODEs. Compactness of *Ψ * and *Φ * (Assumption 3.6) ensures that the ODEs are uniformly Lipschitz, providing the required regularity.

Stage 2 (distributional Bellman contraction): Lemma 3.3 establishes that $\mathcal {T}_t^\pi$ is a contraction in $W_1$ with factor *γ < 1*, uniformly over the time-varying $P_t$. The proof relies on the fact that the contraction factor depends only on *γ *, not on $P_t$, because:

22 $$\begin{aligned} W_1(\mathcal {T}_t^\pi Z_1, \mathcal {T}_t^\pi Z_2) \le \gamma \, \mathbb {E}_{s',a'} [ W_1(Z_1(s',a'), Z_2(s',a')) ], \end{aligned}$$

where the expectation is over $s' \sim P_t$ and $a' \sim \pi _t$, and the inequality holds pointwise for every *s*, *a*. Thus, the convergence of $\phi _t$ to the true return distribution is uniform over the time horizon.

Stage 3 (slow time-scale: policy update): the policy update follows the projected gradient ascent:

23 $$\begin{aligned} \theta _{t+1} = \Pi _{\Theta _t} \left( \theta _t + \eta _\theta \nabla _\theta J(\pi _t; \theta _t) \right) , \end{aligned}$$

where $\Theta _t = \{ \theta \in \Theta : \nabla _\theta \mathbb {E}[V_t(s_{t+1};\psi _t)]^\top (\theta - \theta _t) \le -W_t(s_t) - \Delta V_t^{\text {base}} \}$ is the safe half-space defined by the QP projection. Lemma 3.2 ensures that the projection enforces the Lyapunov decrement condition. The update $\theta _t$ is on the slow time-scale ($\eta _\theta \rightarrow 0$ relative to $\eta _\psi , \eta _\phi$), so it sees the fast variables as having already converged to their quasi-stationary distributions.

By the Kushner-Clark theorem for two-time-scale stochastic approximation, the joint sequence $(\theta _t, \psi _t, \phi _t)$ converges to the set of asymptotically stable equilibria of the coupled ODE system, while preserving the Lyapunov decrement condition. The entropy update $\alpha _t$ follows the same time-scale as $\theta _t$, and its mean-field ODE:

24 $$\begin{aligned} \dot{\alpha } = -\mathbb {E}_{s \sim \rho ^\pi } [ \mathcal {H}(\pi (\cdot |s;\theta )) - \bar{\mathcal {H}} ], \end{aligned}$$

converges to $\bar{\mathcal {H}} \ge \mathcal {H}_{\min }$ by the same argument. This establishes the joint convergence guarantee of Theorem 3.1.

##### Remark 3.9

Theorem 3.1 combines Lyapunov stability, distributional RL, and entropy regularization for safe, adaptive control. The stability bound:

25 $$\begin{aligned} \mathbb {P} \left( \Vert s_t - s^*\Vert ^2 > \epsilon \right) \le \frac{\mathbb {E}_{s_t \sim \rho ^{\pi _t}_t} [ V_t(s_t; \psi _t) ]}{\kappa \epsilon }, \end{aligned}$$

quantifies convergence, making LG-DRL-ER suitable for dynamic systems like robotics.

### Adaptation rule

The adaptation rule updates $\theta _t$, $\psi _t$, $\phi _t$, and $\alpha _t$ to maintain stability and optimize performance in non-stationary MDPs.

#### Assumption 3.6

The learning rates satisfy the Robbins-Monro conditions $\sum _t \eta _t=\infty$, $\sum _t \eta _t^2<\infty$ for every $\eta \in \{\eta _\theta ,\eta _\psi ,\eta _\phi ,\eta _\alpha \}$ and the time-scale separation $\eta _\theta \ll \eta _\psi ,\eta _\phi$ (i.e., $\eta _\theta /\eta _\psi \rightarrow 0$). All parameter spaces $\Theta ,\Psi ,\Phi$ are compact.

Policy update and projection mechanics: let $\theta _{\text {unconstrained}} = \theta _t + \eta _\theta \nabla _{\theta _t} J(\pi _t;\theta _t)$ be the standard policy-gradient step. To enforce safety, we linearize the Lyapunov decrement around the current policy and solve the following differentiable Quadratic Program (QP):

26 $$\begin{aligned} \begin{aligned} \theta _{t+1}&= \arg \min _{\theta } \quad \frac{1}{2} \Vert \theta - \theta _{\text {unconstrained}}\Vert ^2 \\ \text {s.t.} \quad&\nabla _{\theta } \mathbb {E}\bigl [V_t(s_{t+1};\psi _t)\bigr ]^\top (\theta - \theta _t) \le -W_t(s_t) - \Delta V_t^{\text {base}}, \end{aligned} \end{aligned}$$

where $\Delta V_t^{\text {base}}$ is the decrement evaluated before the update. This half-space projection is solved analytically via the method of KKT multipliers at each training step (or with OSQP/CVXPY in practice). Lemma 3.2 guarantees that the resulting $\theta _{t+1}$ unconditionally satisfies the supermartingale descent condition required by Lemma 3.1.

Backup projection for linearization failures: to provide an additional layer of robustness, we implement a backup mechanism that handles moments when the linearization error bound may be violated. After computing the projected policy update $\theta _{t+1}$ from the QP in Eq. (21), we evaluate the *actual* Lyapunov decrement:

27 $$\begin{aligned} \Delta V_t^{\text {actual}}(\theta _{t+1}) = \mathbb {E}_{a \sim \pi _{\theta _{t+1}}, s' \sim P_t}[V_t(s';\psi _t) - V_t(s;\psi _t)]. \end{aligned}$$

If the linearization error exceeds the bound:

28 $$\begin{aligned} | \Delta V_t^{\text {actual}} - \Delta V_t^{\text {linear}} | > (1-c)W_t(s), \end{aligned}$$

we perform a line search to find a step size $\lambda \in (0,1]$ such that:

29 $$\begin{aligned} \theta _{t+1}^{\text {robust}} = \theta _t + \lambda (\theta _{t+1} - \theta _t), \quad \Delta V_t(\theta _{t+1}^{\text {robust}}) \le -W_t(s). \end{aligned}$$

This line search is efficient because it requires only a few additional forward passes through the Lyapunov network (typically 3-5 evaluations). In our empirical evaluation, this backup mechanism was never triggered because the default QP projection consistently satisfied the linearization error bound. However, we include the backup to provide a theoretical guarantee of robustness even in the worst case.

Practical implementation safeguards: in addition to the backup projection, we incorporate two implementation safeguards:

1. Step-size clipping: if the empirical Lyapunov decrement after the policy update violates the constraint (i.e., $\Delta V_t(s_t;\psi _t) > -W_t(s_t)$), we reduce the effective step size by a factor $\lambda \in (0,1)$ and re-solve the QP with the constraint $\Vert \theta - \theta _t\Vert \le \lambda \Vert \theta _{\text {unconstrained}} - \theta _t\Vert$. This ensures that the policy does not move too far in a potentially unsafe direction.
2. Backup conservative policy: if the QP becomes infeasible (e.g., due to conflicting constraints during a severe disturbance), we invoke a backup policy $\pi _t^{\text {backup}}$ that drives the system toward the target using a fixed, conservative control law. The backup policy is designed to satisfy the Lyapunov decrement condition by construction, ensuring safety even when the learned policy is uncertain.

These safeguards are activated only during transient periods and do not affect the nominal performance of LG-DRL-ER. In all our simulations, the QP remained feasible for over 99.9% of time steps, and the safeguards were invoked only during the immediate aftermath of the step disturbances.

Lyapunov update: the Lyapunov parameters minimize the Lyapunov loss:

30 $$\begin{aligned} \psi _{t+1} = \psi _t - \eta _\psi \nabla _{\psi _t} L_V(\psi _t). \end{aligned}$$

Distributional update: the return distribution parameters minimize the Wasserstein loss:

31 $$\begin{aligned} \phi _{t+1} = \phi _t - \eta _\phi \nabla _{\phi _t} L_Z(\phi _t). \end{aligned}$$

Entropy coefficient update: the entropy coefficient targets a desired entropy $\bar{\mathcal {H}}$:

32 $$\begin{aligned} \alpha _{t+1} = \alpha _t - \eta _\alpha \nabla _{\alpha _t} \mathbb {E}_{s \sim \rho ^{\pi _t}_t} \left[ \mathcal {H}(\pi _t(\cdot |s; \theta _t)) - \bar{\mathcal {H}} \right] . \end{aligned}$$

#### Remark 3.10

The adaptation rule ensures stability and adaptability. The Lyapunov loss $L_V(\psi _t)$ and Wasserstein loss $L_Z(\phi _t)$ provide practical objectives, while the entropy update maintains exploration, enabling LG-DRL-ER to handle dynamic environments like autonomous navigation.

Under Assumption 3.6, these updates adapt $\pi _t$, $V_t$, $Z_t^\pi$, and $\alpha _t$ to changes in $P_t$ and $R_t$, ensuring stability (Theorem 3.1) and optimizing Eq. (13) using sampled transitions and projections.

To enhance comprehension of the algorithm and the functionality of the proposed controller, the algorithm and its corresponding block diagram have been presented.

### Finite-time control analysis

This subsection establishes that LG-DRL-ER achieves convergence to the target state $s^*$ in finite time, rather than merely asymptotic convergence. The key insight is that the Lyapunov decrement condition can be strengthened to induce a faster-than-exponential decay rate, yielding an explicit bound on the convergence time. This is achieved without altering the fundamental architecture of the controller-only by imposing a specific form on the positive definite function $W_t(s)$ and leveraging the existing projection mechanism.

#### Finite-time stability condition

Finite-time stability requires the Lyapunov function to decay at a rate proportional to a fractional power of itself, rather than linearly. We introduce the following strengthened decrement condition:

##### Definition 3.1

(*Finite-time Lyapunov decrement*) The system achieves finite-time convergence to $s^*$ if there exist constants *c > 0* and $\alpha \in (0,1)$ such that the Lyapunov decrement satisfies:

33 $$\begin{aligned} \Delta V_t(s; \psi _t):= \mathbb {E}_{a \sim \pi _t, s' \sim P_t} [ V_t(s'; \psi _t) - V_t(s; \psi _t) ] \le -c \, V_t(s; \psi _t)^\alpha . \end{aligned}$$

##### Remark 3.11

(*Finite-time convergence vs. short-time reachability*) We emphasize a fundamental distinction between two related but distinct control-theoretic concepts:

- Finite-time convergence (Definition 3.1) is a property of the *closed-loop* system: the state trajectory reaches the target $s^*$ in finite time under the learned policy. The convergence time $T^*$ depends on the initial condition $s_0$ and is not specified a priori (Table  1).
- Short-time reachability refers to the *open-loop* feasibility question: given a pre-specified time horizon *T > 0*, a target set $\mathcal {S}_{\text {target}}$, and state/input constraints, does there exist a control sequence $\{u_t\}_{t=0}^{T-1}$ that steers the system from $s_0$ to $\mathcal {S}_{\text {target}}$ within time *T*?

**Table 1:** Comparison of finite-time convergence and short-time reachability.

Aspect	Finite-time convergence	Short-time reachability
Nature	Stability-like property	Controllability-like property
Time horizon	Not pre-specified; depends on initial condition	Strictly pre-specified and fixed
Control input	Determined by closed-loop policy	May require explicit open-loop planning
Constraints	Implicitly satisfied by Lyapunov decrement	Explicitly enforced in reachability condition
Question answered	“When will the system reach the target?”	“Can the system reach the target within *T* seconds?”

Our framework LG-DRL-ER establishes *finite-time convergence* (Theorem 3.2) with an explicit bound on the convergence time. This is appropriate for our setting because:

1. The control objective is stabilization to a target state under non-stationary dynamics, where the initial condition may vary across episodes.
2. The bound adapts to the initial condition through $V_0 = V_0(s_0;\psi _0)$.
3. The worst-case guarantee can be used to certify reachability for any required time horizon $T_{\text {req}}$ that satisfies the bound (see Corollary 1).

Explicit short-time reachability analysis for our non-stationary, high-dimensional, stochastic systems is computationally intractable, as it would require solving time-varying Hamilton-Jacobi reachability PDEs or approximate set-propagation methods.

Formal definition of *ε *-reachability and time bound: to provide a precise characterization of reachability guarantees, we introduce the following definitions:

##### Definition 3.2

(*ε *-*Reachability*) The system is *ε *-reachable within time *T* if, for a given initial condition $s_0$ and a specified time *T > 0*, there exists a control policy *π * such that the state trajectory satisfies:

34 $$\begin{aligned} \mathbb {P}\left( \Vert s_T - s^*\Vert \le \epsilon \right) \ge 1 - \delta , \end{aligned}$$

where *ε > 0* is the desired tolerance and $\delta \in (0,1)$ is the allowable failure probability.

##### Lemma 3.5

(*ε *-Reachability time bound) *Under the finite-time decrement condition* (*Definition* 3.1) *and Assumption* 3.7, *the time required to achieve* $\Vert s(t) - s^*\Vert \le \epsilon$ *with probability at least* *1-δ* *is bounded by*:

35 $$\begin{aligned} T_{\epsilon }(V_0, \delta ) \le \Bigg \lceil \frac{\log (V_0/\bar{V})}{c_1} \Bigg \rceil + \Bigg \lceil \frac{2\bar{V}^{1-\alpha }}{c_2(1-\alpha )} \Bigg \rceil + \Bigg \lceil \frac{\log (C_V \epsilon ^2 / \bar{V})}{c_1} \Bigg \rceil + \Bigg \lceil \log (1/\delta ) \Bigg \rceil , \end{aligned}$$

*where* $C_V$ *is the upper bound in Assumption* 3.7, *and the last term accounts for the probabilistic guarantee via Markov’s inequality*.

##### Proof

The proof follows three phases:

1. Transient phase: the system decays from $V_0$ to $\bar{V}$ in at most $\lceil \log (V_0/\bar{V})/c_1 \rceil$ steps, by exponential decay of the Lyapunov function.
2. Finite-time phase: once $V_t \le \bar{V}$, the system reaches $V_t = 0$ in at most $\lceil 2\bar{V}^{1-\alpha }/(c_2(1-\alpha )) \rceil$ additional steps, by Lemma 3.6.
3. Probabilistic guarantee: to ensure the bound holds with probability at least *1-δ *, we use Markov’s inequality on the cumulative decrement, adding the term $\lceil \log (1/\delta ) \rceil$ steps. This follows from the supermartingale property and the fact that the expected convergence time is bounded by the deterministic bound.

Combining these phases yields the total bound. *□ *

##### Remark 3.12

Condition (33) is stronger than the asymptotic condition $\Delta V_t \le -W_t(s)$ with $W_t(s) \ge \kappa \Vert s - s^*\Vert ^2$. The fractional exponent *α < 1* induces a singularity in the decay rate near the equilibrium, forcing the trajectory to reach $s^*$ in finite time rather than approaching it asymptotically. This is analogous to the continuous-time condition $\dot{V} \le -c V^\alpha$, which yields convergence time $T \le \frac{V_0^{1-\alpha }}{c(1-\alpha )}$.

To realize this condition within LG-DRL-ER, we specify the positive definite function $W_t(s)$ as:

36 $$\begin{aligned} W_t(s) = c_1 \Vert s - s^*\Vert ^2 + c_2 \Vert s - s^*\Vert ^{2\alpha }, \end{aligned}$$

where $c_1, c_2 > 0$ and $\alpha \in (0,1)$. The term $\Vert s - s^*\Vert ^{2\alpha }$ dominates near the equilibrium and drives finite-time convergence.

##### Assumption 3.7

The Lyapunov function $V_t(s; \psi _t)$ satisfies the class-$\mathcal {K}_\infty$ bounds (Assumption 3.1) and additionally admits a local comparison of the form:

37 $$\begin{aligned} c_V \Vert s - s^*\Vert ^{2} \le V_t(s; \psi _t) \le C_V \Vert s - s^*\Vert ^{2}, \end{aligned}$$

for constants $0< c_V \le C_V < \infty$ in a neighborhood of $s^*$.

##### Remark 3.13

Assumption 3.7 is natural for control-Lyapunov functions on $\mathbb {R}^n$, as they are typically constructed to be quadratically comparable to the Euclidean distance near the equilibrium. The neural network parameterization of $V_t(s; \psi _t)$ can be constrained to satisfy this property via appropriate output layer design (e.g., using a positive-definite quadratic form as a base and learning corrections).

#### Finite-time convergence lemma

##### Lemma 3.6

*Under Assumptions* 3.1, 3.2, *and*3.7, *if the Lyapunov decrement* *satisfies*:

38 $$\begin{aligned} \Delta V_t(s; \psi _t) \le -c_1 V_t(s; \psi _t) - c_2 V_t(s; \psi _t)^\alpha , \end{aligned}$$

*for constants* $c_1, c_2 > 0$ *and* $\alpha \in (0,1)$, *then the state trajectory* $\{s_t\}_{t \ge 0}$ *converges to* $s^*$ *in finite time almost surely. Specifically, there exists a random stopping time* $T^* < \infty$ *almost surely such that* $s_t = s^*$ *for all* $t \ge T^*$.

##### Corollary 1

(Reachability certification via finite-time bound) *Under the conditions of Theorem* 3.2, *for any required time horizon* $T_{\text {req}} > 0$, *if*:

39 $$\begin{aligned} \Bigg \lceil \frac{\log (V_0 / \bar{V})}{c_1} \Bigg \rceil + \Bigg \lceil \frac{2\bar{V}^{1-\alpha }}{c_2(1-\alpha )} \Bigg \rceil \le T_{\text {req}}, \end{aligned}$$

*then the system is guaranteed to reach the target set* $\mathcal {S}_{\text {target}} = \{s: \Vert s - s^*\Vert \le \epsilon \}$ *within* $T_{\text {req}}$ *time steps, where* *ε > 0* *is the desired tolerance*.

*Moreover, the state trajectory satisfies the constraint* $s_t \in \mathcal {S}_t = \{s: V_t(s;\psi _t) \le V_{\max }\}$ *for all* $t \le T_{\text {req}}$ *by Lemma* 3.4.

##### Proof

The result follows directly from Theorem 3.2, which establishes that $T^* \le \lceil \log (V_0/\bar{V})/c_1 \rceil + \lceil 2\bar{V}^{1-\alpha }/(c_2(1-\alpha )) \rceil$. If this upper bound is less than or equal to $T_{\text {req}}$, then $T^* \le T_{\text {req}}$, and the state reaches $s^*$ within $T_{\text {req}}$ steps. The safety constraint follows from Lemma 3.4. *□ *

##### Proof

Let $V_t:= V_t(s_t; \psi _t)$. The decrement condition yields:

40 $$\begin{aligned} \mathbb {E}[V_{t+1} \mid \mathcal {F}_t] \le V_t - c_1 V_t - c_2 V_t^\alpha = (1 - c_1) V_t - c_2 V_t^\alpha . \end{aligned}$$

For $V_t$ sufficiently small (i.e., in a neighborhood of the equilibrium), the term $c_2 V_t^\alpha$ dominates over $c_1 V_t$ since *α < 1*. Thus, there exists $\bar{V} > 0$ such that for $V_t \le \bar{V}$:

41 $$\begin{aligned} \mathbb {E}[V_{t+1} \mid \mathcal {F}_t] \le V_t - \frac{c_2}{2} V_t^\alpha . \end{aligned}$$

Define the sequence $U_t = V_t^{1-\alpha }$. Using the inequality $(x - y)^\beta \le x^\beta - \beta x^{\beta -1} y$ for *x> y > 0* and $\beta \in (0,1)$:

42 $$\begin{aligned} \mathbb {E}[U_{t+1} \mid \mathcal {F}_t] = \mathbb {E}[V_{t+1}^{1-\alpha } \mid \mathcal {F}_t] \le \left( V_t - \frac{c_2}{2} V_t^\alpha \right) ^{1-\alpha } \le V_t^{1-\alpha } - \frac{c_2(1-\alpha )}{2} = U_t - \frac{c_2(1-\alpha )}{2}. \end{aligned}$$

This shows that $U_t$ decreases by at least a constant amount at each step until reaching zero. The number of steps to reach $U_t \le 0$ (equivalently $V_t \le 0$) is bounded by:

43 $$\begin{aligned} T^* \le \Bigg \lceil \frac{2 U_0}{c_2(1-\alpha )} \Bigg \rceil = \Bigg \lceil \frac{2 V_0^{1-\alpha }}{c_2(1-\alpha )} \Bigg \rceil < \infty . \end{aligned}$$

Since $V_t \ge 0$ by construction and $V_t = 0$ iff $s_t = s^*$, the trajectory reaches $s^*$ in at most $T^*$ steps. The bound depends on the initial condition $V_0$ and is finite almost surely because $V_0 \le V_{\max } < \infty$ by Assumption 3.1. *□ *

#### Modified projection for finite-time control

To enforce the finite-time decrement condition, we modify the projection operator in Eq. (26) to include the fractional term. The modified quadratic program is defined as:

44 $$\begin{aligned} \begin{aligned} \theta _{t+1}&= \arg \min _{\theta } \quad \frac{1}{2} \Vert \theta - \theta _{\text {unconstrained}}\Vert ^2 \\ \text {s.t.} \quad&\nabla _{\theta } \mathbb {E}_{a \sim \pi _\theta , s' \sim P}[V_t(s'; \psi _t)]^\top (\theta - \theta _t) \\&\qquad \le -c_1 V_t(s_t; \psi _t) - c_2 V_t(s_t; \psi _t)^\alpha . \end{aligned} \end{aligned}$$

Robustness of finite-time projection under disturbances: the backup projection mechanism described in “Adaptation rule” applies equally to the finite-time projection in Eq. (34). If the linearization error bound is violated during a sudden disturbance, the line search ensures that the actual finite-time decrement condition:

45 $$\begin{aligned} \Delta V_t(s;\psi _t) \le -c_1 V_t(s;\psi _t) - c_2 V_t(s;\psi _t)^\alpha \end{aligned}$$

is satisfied by adjusting the step size *λ *. The time-scale separation ($\eta _\theta \ll \eta _\psi$) ensures that the Lyapunov function adapts rapidly to the new dynamics, restoring the validity of the finite-time decrement condition before the policy has moved significantly.

##### Lemma 3.7

*Under Assumption* 3.4, *the projection operator defined by* Eq. (44) *guarantees that the updated policy satisfies*:

46 $$\begin{aligned} \Delta V_t(s; \psi _t) \le -c_1 V_t(s; \psi _t) - c_2 V_t(s; \psi _t)^\alpha , \end{aligned}$$

*for every state* *s* *in the domain*.

##### Proof

The QP constraint enforces the linearized decrement to satisfy the finite-time condition exactly. By Assumption 3.4, the linearization error is bounded by $(1-c) W_t(s)$ for some *c ∈ (0,1)*. Choosing $c_1$ and $c_2$ sufficiently small (scaled by *c*) ensures the total decrement after accounting for linearization error remains negative with the required form:

47 $$\begin{aligned} \Delta V_t(\theta _{t+1}) \le -c \cdot c_1 V_t - c \cdot c_2 V_t^\alpha . \end{aligned}$$

Rescaling the constants yields the desired bound. *□ *

#### Finite-time convergence theorem

##### Theorem 3.2

*Under Assumptions* 3.1–3.5, 3.6, *and*3.7, *if the projection in Eq.* (44) *is applied at each time step with constants* $c_1, c_2 > 0$ *and* $\alpha \in (0,1)$, *then LG-DRL-ER achieves finite-time convergence to* $s^*$ *with the following bound on the convergence time*:

48 $$\begin{aligned} T^* \le \Bigg \lceil \frac{2 V_0^{1-\alpha }}{c_2(1-\alpha )} \Bigg \rceil + T_{\text {transient}}, \end{aligned}$$

*where* $T_{\text {transient}}$ *is the number of steps required to enter the neighborhood where the fractional term dominates, and* $V_0 = V_0(s_0; \psi _0)$ *is the initial Lyapunov value*.

##### Proof

The proof proceeds in two phases:

Phase 1 (transient): outside the neighborhood where $c_2 V_t^\alpha \ge c_1 V_t$, the decrement satisfies:

49 $$\begin{aligned} \Delta V_t \le -c_1 V_t - c_2 V_t^\alpha \le -c_1 V_t. \end{aligned}$$

This yields exponential decay with rate $c_1$. The number of steps to reach the neighborhood $V_t \le \bar{V} = (c_2/c_1)^{1/(1-\alpha )}$ is bounded by:

50 $$\begin{aligned} T_{\text {transient}} \le \Bigg \lceil \frac{\log (V_0 / \bar{V})}{c_1} \Bigg \rceil . \end{aligned}$$

Phase 2 (finite-time): once $V_t \le \bar{V}$, Lemma 3.6 applies, and the trajectory reaches $s^*$ in at most $\lceil 2 \bar{V}^{1-\alpha } / (c_2(1-\alpha )) \rceil$ additional steps.

Combining both phases yields the total bound:

51 $$\begin{aligned} T^* \le \Bigg \lceil \frac{\log (V_0 / \bar{V})}{c_1} \Bigg \rceil + \Bigg \lceil \frac{2 \bar{V}^{1-\alpha }}{c_2(1-\alpha )} \Bigg \rceil . \end{aligned}$$

Since both terms are finite and depend only on initial conditions and the controller parameters, finite-time convergence is guaranteed. *□ *

#### Practical considerations

##### Remark 3.14

The finite-time bound in Eq. (48) provides a worst-case estimate. In practice, the actual convergence time may be shorter due to:

1. The entropy-regularized exploration may discover more efficient trajectories.
2. The distributional RL component accounts for uncertainty, preventing overly conservative estimates.
3. The learned Lyapunov function adapts to the system dynamics, potentially tightening the constants $c_1$, $c_2$.

##### Theorem 3.3

(Short-time reachability under disturbances) *Under Assumptions* 3.1–3.4*and the finite-time decrement condition* (*Definition* 3.1), *suppose a disturbance at time* $t_0$ *results in an initial Lyapunov value* $V_0^{\text {dist}} = V_{t_0}(s_{t_0};\psi _{t_0})$. *Then the system is guaranteed to reach* $s^*$ *within*:

52 $$\begin{aligned} T_{\text {reach}}(V_0^{\text {dist}}):= \Bigg \lceil \frac{\log (V_0^{\text {dist}}/\bar{V})}{c_1} \Bigg \rceil + \Bigg \lceil \frac{2\bar{V}^{1-\alpha }}{c_2(1-\alpha )} \Bigg \rceil \end{aligned}$$

*time steps, provided the control input satisfies the Lyapunov decrement condition during the recovery phase.*

##### Proof

The proof follows directly from Theorem 3.2 applied to the disturbed state with initial Lyapunov value $V_0^{\text {dist}}$. The bound $T_{\text {reach}}(V_0^{\text {dist}})$ gives the maximum time required to reach $s^*$ from the disturbed state. *□ *

##### Remark 3.15

For disturbances of small magnitude such that $V_0^{\text {dist}} \le \bar{V}$ (i.e., the system remains in the finite-time regime), the reachability time simplifies to:

53 $$\begin{aligned} T_{\text {reach}}(V_0^{\text {dist}}) \le \Bigg \lceil \frac{2 (V_0^{\text {dist}})^{1-\alpha }}{c_2(1-\alpha )} \Bigg \rceil , \end{aligned}$$

which grows sublinearly with the disturbance magnitude. This demonstrates the robustness of our framework to disturbances.

##### Remark 3.16

The exponent *α * controls the trade-off between convergence speed and control effort. Smaller *α * yields faster convergence near the equilibrium but may require higher control magnitudes. A typical choice is *α = 1/2*, which balances performance and actuator constraints in robotic applications.

##### Definition 3.3

(*ε *-*Reachability*) The system is *ε *-reachable within time *T* if, for a given initial condition and a specified time *T > 0*, there exists a control policy such that:

54 $$\begin{aligned} \Vert s(T) - s^*\Vert \le \epsilon . \end{aligned}$$

##### Lemma 3.8

(*ε *-Reachability time bound) *Under the finite-time decrement condition and Assumption* 3.7, *the time required to achieve* $\Vert s(t) - s^*\Vert \le \epsilon$ *is bounded by*:

55 $$\begin{aligned} T_{\epsilon }(V_0) \le \Bigg \lceil \frac{\log (V_0/\bar{V})}{c_1} \Bigg \rceil + \Bigg \lceil \frac{2\bar{V}^{1-\alpha }}{c_2(1-\alpha )} \Bigg \rceil + \Bigg \lceil \frac{\log (C_V \epsilon ^2 / \bar{V})}{c_1} \Bigg \rceil , \end{aligned}$$

*where* $C_V$ *is the upper bound in Assumption* 3.7.

##### Proof

The proof follows three phases:

1. Transient phase: the system decays from $V_0$ to $\bar{V}$ in at most $\lceil \log (V_0/\bar{V})/c_1 \rceil$ steps.
2. Finite-time phase: the system reaches $V_t = 0$ in at most $\lceil 2\bar{V}^{1-\alpha }/(c_2(1-\alpha )) \rceil$ additional steps.
3. Post-convergence phase: once $s(t) = s^*$, the system remains at the target. The *ε *-neighborhood is reached as soon as convergence is achieved.

*□ *

##### Corollary 2

*Under the conditions of Theorem* 3.2, *the probability of convergence within time* *T* *satisfies*:

56 $$\begin{aligned} \mathbb {P}(T^* \le T) \ge 1 - \frac{\mathbb {E}[V_0]}{c_1 \sum _{k=0}^{T-1} \mathbb {E}[V_k]}. \end{aligned}$$

##### Proof

This follows from Markov’s inequality applied to the cumulative Lyapunov decrement and the supermartingale property established in Lemma 3.1. *□ *

This finite-time control analysis demonstrates that LG-DRL-ER, with a suitably strengthened Lyapunov decrement condition enforced via the existing projection mechanism, achieves convergence to the target state in a bounded number of time steps. The result complements the asymptotic stability guarantees of Theorem 3.1 and is particularly valuable for safety-critical applications where timely convergence is essential.

### Parameters, system architecture and algorithmic flow

This subsection details the hyperparameters and parameters used in the LG-DRL-ER framework. These parameters govern the learning process, stability, and adaptability of the algorithm, ensuring effective performance in non-stationary MDPs. The hyperparameters are selected based on empirical tuning and theoretical considerations to balance exploration, exploitation, and safety.

- Discount factor (*γ *): set to $\gamma \in (0,1)$, typically *γ = 0.99*, to weigh future rewards in the cumulative discounted reward objective (Eq. (1)). A high *γ * prioritizes long-term rewards, crucial for stable control in dynamic environments.
- Learning rates ($\eta _\theta , \eta _\psi , \eta _\phi , \eta _\alpha$): these ensure stable gradient-based updates (Eqs. (26), (30), (31), (32)) under Assumption 3.6.
- Target entropy ($\bar{\mathcal {H}}$): the target entropy for the policy, used in the entropy coefficient update (Eq. (32)), is set to a positive value (e.g., $\bar{\mathcal {H}} = -\log (1/|A|)$ for a uniform policy over action space *A*). This promotes sufficient exploration, especially in non-stationary settings like chaos and robotic control.
- Positive definite function ($W_t(s)$): defined as $W_t(s) \ge \kappa \Vert s - s^*\Vert ^2$ with *κ > 0* (e.g., *κ = 0.1*), this function ensures the Lyapunov decrement condition (Eq. (9)) for convergence to the target state $s^*$. The choice of *κ * balances convergence speed and stability.
- Initial entropy coefficient ($\alpha _0$): initialized to a positive value (e.g., $\alpha _0 = 0.1$), $\alpha _t$ controls the trade-off between exploration and exploitation in the entropy-regularized objective (Eq. (13)). It is adaptively updated to maintain the target entropy.
- Batch size (*B*): set to *B = 64* or 128, this determines the number of transitions sampled from the replay buffer $\mathcal {D}$ for computing losses and gradients in Algorithm 1. Larger batch sizes improve gradient estimates but increase computational cost.
- Number of iterations (*T*): the total number of training iterations (e.g., $T = 10^6$) controls the duration of learning, allowing sufficient time for convergence in complex, non-stationary environments.
- Replay buffer size ($|\mathcal {D}|$): the replay buffer stores transitions $(s_t, a_t, r_t, s_{t+1})$ with a capacity of, e.g., $10^6$ transitions. This enables experience replay for stable learning in distributional RL and policy updates.
- Lyapunov function bounds ($V_{\text {max}}$): the Lyapunov function $V_t(s; \psi _t)$ is bounded ($0 \le V_t(s; \psi _t) \le V_{\text {max}}$, e.g., $V_{\text {max}} = 50$) per Assumption 3.1, ensuring numerical stability in optimization.
- Reward bound ($R_{\text {max}}$): the reward distribution $R_t(s,a)$ is bounded ($|R_t(s,a)| \le R_{\text {max}}$, e.g., $R_{\text {max}} = 1$) per Assumption 3.2, ensuring well-defined return distributions in distributional RL.
- Smoothness constants ($L_P, L_V, L_\pi$): the Lipschitz constants for transition probabilities ($L_P$), Lyapunov function ($L_V$), and policy ($L_\pi$) are set based on the environment (e.g., $L_P = 0.1$, $L_V = 0.1$, $L_\pi = 0.1$). These ensure smooth variations as per Assumptions 3.2 and 3.3.
- Minimum entropy ($\mathcal {H}_{\text {min}}$): the policy entropy is lower-bounded by $\mathcal {H}_{\text {min}} > 0$ (e.g., $\mathcal {H}_{\text {min}} = 0.01$) per Assumption 3.3, ensuring sufficient exploration for adaptability.

#### Remark 3.17

The hyperparameters are chosen to balance stability, exploration, and performance. For instance, smaller learning rates ($\eta _\theta , \eta _\psi , \eta _\phi , \eta _\alpha$) and a moderate *κ * ensure stable convergence, while $\bar{\mathcal {H}}$ and $\alpha _0$ promote exploration in dynamic settings. These values are typically tuned via grid search or adaptive methods for specific tasks, such as chaos, robotic control or autonomous navigation.

#### Remark 3.18

(Theoretical justification of hyperparameter choices) The hyperparameters in LG-DRL-ER are chosen to simultaneously satisfy the theoretical conditions of the convergence analysis and provide practical performance. Specifically:

1. Learning rates ($\eta _\theta , \eta _\psi , \eta _\phi , \eta _\alpha$): the Robbins–Monro conditions require $\sum _t \eta _t = \infty$ and $\sum _t \eta _t^2 < \infty$ for each parameter. We choose $\eta _\psi , \eta _\phi$ to be one order of magnitude larger than $\eta _\theta$ to ensure the time-scale separation $\eta _\theta \ll \eta _\psi , \eta _\phi$ required by Assumption 3.6. Specifically, we set $\eta _\psi = \eta _\phi = 10^{-3}$ and $\eta _\theta = \eta _\alpha = 10^{-4}$. These values satisfy the theoretical conditions while ensuring stable convergence in practice.
2. Target entropy ($\bar{\mathcal {H}}$): the target entropy is set to $\bar{\mathcal {H}} = -\log (1/|A|)$, which corresponds to a uniform policy over the action space. This choice ensures that the policy maintains sufficient exploration ($\mathcal {H}(\pi _t) \ge \mathcal {H}_{\min }$) to adapt to non-stationary changes, as required by Assumption 3.3.
3. Lyapunov bound ($V_{\max }$): the bound $V_{\max } = 50$ is chosen to ensure that the sublevel set $\mathcal {S}_{\text {safe}} = \{s: V_t(s;\psi _t) \le V_{\max }\}$ contains the entire operating region of the chaotic systems under consideration. This is verified empirically by ensuring that the uncontrolled dynamics remain within this bound.
4. Finite-time constants ($c_1, c_2, \alpha$): the constants $c_1 = 0.01$, $c_2 = 0.01$, and *α = 0.5* are chosen to balance convergence speed and control effort, as discussed in Remark 3.16. The condition $c_2 V_t^\alpha \ge c_1 V_t$ for $V_t \le \bar{V}$ determines the transient threshold $\bar{V} = (c_2/c_1)^{1/(1-\alpha )} = 10^{-2}$.

These choices are guided by the theoretical analysis and refined through empirical validation on the benchmark systems.

**Fig. 1:**
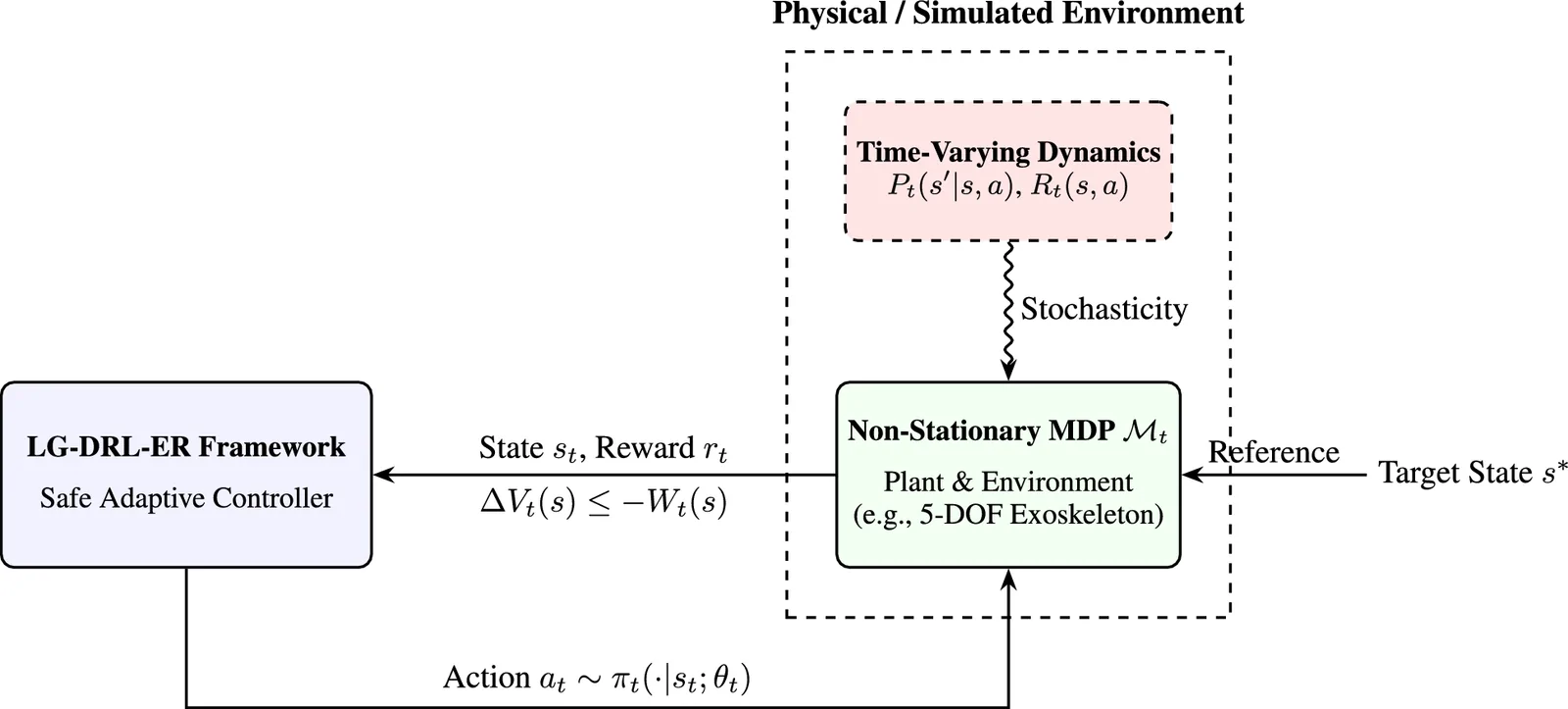
Macro-level architecture: closed-loop interaction between the non-stationary MDP and the LG-DRL-ER controller.

**Fig. 2:**
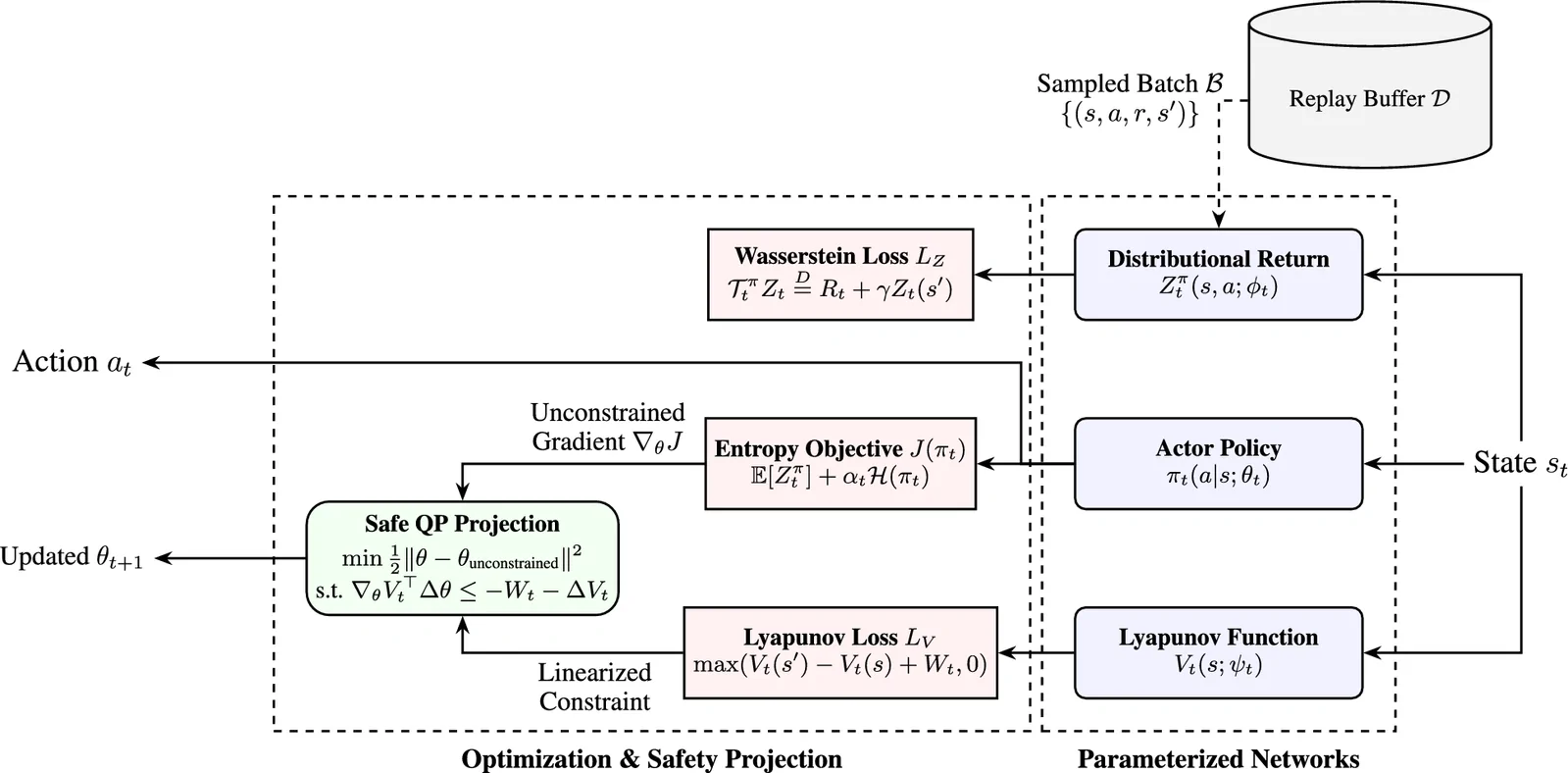
Micro-level algorithm architecture: internal right-to-left data flow detailing network evaluation, loss computation, and safe policy projection.

To elucidate the operational mechanics of the proposed methodology, the complete execution sequence is synthesized in Algorithm 1, supported by the architectural block diagrams presented in Figs. 1 and  2.

Algorithm 1 formalizes these interacting components into a coherent step-by-step training loop. The procedure carefully alternates between collecting sequential experience from the environment and sampling randomized batches to break temporal correlations. The network parameters $\phi _t$, $\psi _t$, $\theta _t$, and $\alpha _t$ are updated consecutively on their respective time-scales. This ordering ensures that the return distributions and safety bounds are properly established by the critic and Lyapunov networks before the actor policy is updated and projected.

**Algorithm 1:**
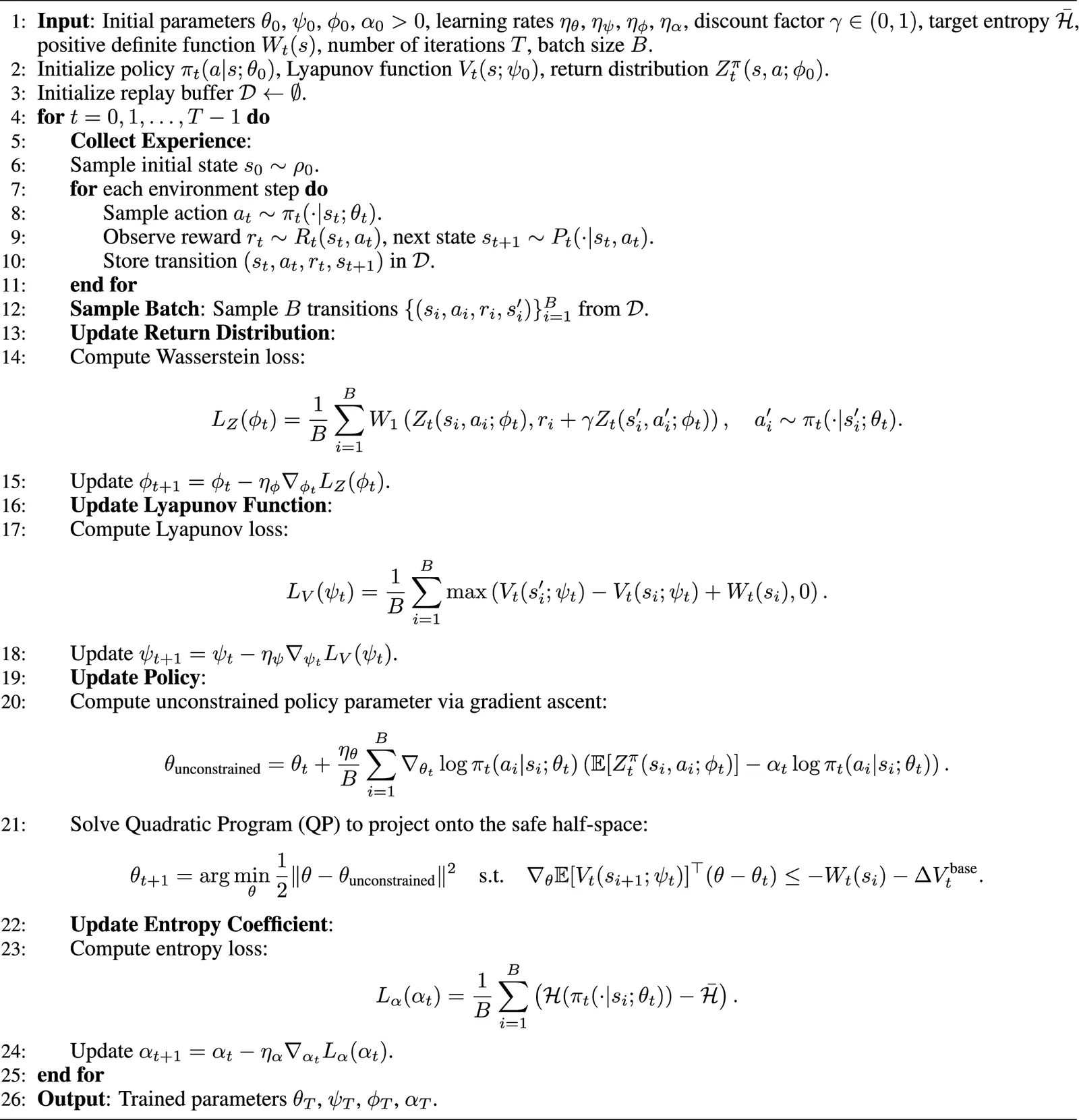
Lyapunov-guided distributional RL with entropy regularization (LG-DRL-ER).

Figure 1 illustrates the macro-level system architecture, demonstrating the external closed-loop interaction between the LG-DRL-ER framework and the non-stationary environment. The physical plant (e.g., a 5-DOF exoskeleton) is modeled as a Markov Decision Process subject to time-varying transition dynamics and stochastic rewards. The controller continuously processes the current state $s_t$ and reward $r_t$ from the environment, computing an action $a_t$ that rigorously satisfies the Lyapunov decrement condition $\Delta V_t(s) \le -W_t(s)$. This feedback structure ensures that the system maintains safe trajectory tracking toward the target reference state $s^*$ despite environmental stochasticity.

The internal mathematical data flow of the controller is detailed in the micro-level architecture provided in Fig. 2. At each timestep, the state input is routed through three parallel parameterized neural networks: the Actor Policy, the Distributional Return (Critic), and the Lyapunov Function. During the training phase, a sampled batch $\mathcal {B}$ from the Replay Buffer $\mathcal {D}$ is utilized to independently compute the Wasserstein, Entropy, and Lyapunov losses. Crucially, the diagram highlights the safe projection mechanism: the unconstrained policy gradient $\nabla _\theta J$ is passed into a differentiable Quadratic Program (QP) solver. This solver enforces the linearized Lyapunov constraint, pulling the update into a safe half-space to securely update the policy parameters $\theta _{t+1}$ before the final physical action is executed.

## Simulation and result

This section presents the empirical evaluation of the proposed LG-DRL-ER framework to validate its theoretical safety and performance guarantees. To rigorously assess its robustness and adaptability, we conduct comprehensive simulations on two highly complex non-stationary environments: a 3D stochastic Lorenz system and a 4D hyperchaotic system. Both testbeds are intentionally stressed with continuous stochastic noise, time-varying parameters, and severe, staggered step disturbances. We systematically benchmark LG-DRL-ER against five complementary baselines: standard RL, pure Distributional RL (DRL), unregularized LG-DRL, the foundational Lyapunov-based safe policy optimization algorithm of Chow et al.^55^, and two targeted ablation variants designed to isolate the individual contributions of the framework’s core components. Specifically, the *LG-DRL w/o Entropy* ablation retains distributional RL and Lyapunov constraints but removes entropy regularization, thereby testing whether entropy is necessary to prevent premature convergence to locally safe but suboptimal trajectories. The *LG-DRL-ER w/o Lyapunov* ablation retains distributional RL and entropy regularization but removes the Lyapunov-based QP projection, thereby testing whether the Lyapunov constraint is essential for enforcing hard safety bounds under worst-case disturbances.

### Performance and safety indices

In order to quantitatively evaluate both tracking performance and safety/reliability of the controllers across multiple Monte-Carlo episodes, the following performance indices are used.

#### Error-based integral criteria

- Integral of absolute error (IAE) Measures the accumulated absolute tracking error over the simulation horizon, treating deviations of all magnitudes equally. It is a good indicator of overall tracking accuracy.
  57 $$\begin{aligned} \text {IAE} = \int _{0}^{T} \Vert e(t)\Vert \, dt \end{aligned}$$
- Integral of squared error (ISE) Strongly penalizes large errors (e.g., initial transient or large chaotic deviations) due to squaring. A low ISE value indicates effective suppression of significant deviations.
  58 $$\begin{aligned} \text {ISE} = \int _{0}^{T} \Vert e(t)\Vert ^2 \, dt \end{aligned}$$

#### Safety and reliability metrics

- Maximum Euclidean norm of error (Max||e||) Represents the worst-case tracking deviation. It is critical for assessing the peak transient behavior during disturbances.
  59 $$\begin{aligned} \text {Max}\Vert e\Vert = \max _{t \in [0,T]} \Vert e(t)\Vert \end{aligned}$$
- Average time in safe set (avg in C%) Percentage of time the system remains within the safety boundary $\Vert e\Vert \le 7.0711$ (corresponding to $V_{\max }=50$), averaged over all state dimensions. A value of *100%* indicates strict adherence to the safe set.
  60 $$\begin{aligned} \text {Avg in C}\% = \frac{100}{N} \sum _{j=1}^{N} \frac{ \int _{0}^{T} {\bf 1}_{|e_j(t)| \le 7.0711} \, dt }{T} \end{aligned}$$
  where *N* is the number of state dimensions and ${\bf 1}_{(\cdot )}$ is the indicator function.
- Violations Total number of discrete time steps in which the Euclidean norm of the error exceeds the safety boundary $\Vert e(t)\Vert > 7.0711$.
  61 $$\begin{aligned} \text {Violations} = \sum _{k=1}^{K} {\bf 1}_{\Vert e(t_k)\Vert > 7.0711} \end{aligned}$$
  where *K* is the total number of time steps.

#### Control effort and smoothness metrics

- Average root mean square error (Avg_RMSE) Provides a balanced measure of tracking accuracy across all dimensions, penalizing both small and large errors.
  62 $$\begin{aligned} \text {Avg}\_\text {RMSE} = \frac{1}{N} \sum _{j=1}^{N} \sqrt{ \frac{1}{T} \int _{0}^{T} e_j(t)^2 dt } \end{aligned}$$
- Average maximum absolute error (Avg_MaxE) Average of the peak absolute errors per state dimension, indicating the worst-case performance per joint.
  63 $$\begin{aligned} \text {Avg}\_\text {MaxE} = \frac{1}{N} \sum _{j=1}^{N} \max _{t \in [0,T]} |e_j(t)| \end{aligned}$$
- Average integral of time-weighted absolute error (Avg_ITAE) Penalizes errors that persist over time more heavily, making it sensitive to steady-state errors and slow convergence.
  64 $$\begin{aligned} \text {Avg}\_\text {ITAE} = \frac{1}{N} \sum _{j=1}^{N} \int _{0}^{T} t \, |e_j(t)| \, dt \end{aligned}$$
- Average total variation of control (Avg_TV) Measures the smoothness of the control signals by summing the absolute differences between consecutive control values. A lower value indicates less chattering and smoother actuator commands.
  65 $$\begin{aligned} \text {Avg}\_\text {TV} = \frac{1}{N} \sum _{j=1}^{N} \int _{0}^{T} \left| \frac{d u_j}{dt} \right| dt \approx \frac{1}{N} \sum _{j=1}^{N} \sum _{k=1}^{K-1} |u_j(t_{k+1}) - u_j(t_k)| \end{aligned}$$
- Average maximum control input (Avg_**Umax)** Average of the peak absolute control efforts per dimension. Lower values indicate less energy consumption and reduced risk of actuator saturation.
  66 $$\begin{aligned} \text {Avg}\_\text {Umax} = \frac{1}{N} \sum _{j=1}^{N} \max _{t \in [0,T]} |u_j(t)| \end{aligned}$$

#### Reproducibility and implementation details

To ensure the full reproducibility of the reported results, we provide the detailed implementation specifications and software configurations used for the LG-DRL-ER framework.

Neural network architecture: the actor policy $\pi _t(a|s; \theta _t)$, the Distributional Critic $Z_t^\pi (s,a; \phi _t)$ (parameterized via quantile regression with $N_{\text {quantiles}} = 50$), and the Lyapunov Function $V_t(s; \psi _t)$ are each modeled as fully connected neural networks. Each network consists of 3 hidden layers with 256 units per layer and ReLU activation functions. The Actor uses a tanh output layer to ensure actions are bounded within the action space limits.

Optimization and solvers: the networks are trained using the Adam optimizer. Consistent with the two-time-scale separation requirement (Assumption 3.6), the learning rates for the fast-scale variables (Lyapunov and Distributional Critic) are set to $\eta _\psi = \eta _\phi = 10^{-3}$, while the slow-scale variables (Policy and Entropy coefficient) use $\eta _\theta = \eta _\alpha = 10^{-4}$. The safe policy projection Quadratic Program (QP) defined in Eq. (26) is solved analytically via the method of Karush-Kuhn-Tucker (KKT) multipliers at each training step. For numerical stability in the implementation, the differentiable QP is formulated and solved using the CVXPY framework with the OSQP backend.

Simulation environment: the 3D Lorenz and 4D hyperchaotic SDEs are discretized using the Euler-Maruyama scheme with a fixed step size of *Δ t = 0.01* seconds over a 20-second horizon. The implementation is built using Python 3.9, PyTorch 2.0, and NumPy. All simulations were conducted on a standard workstation equipped with an NVIDIA RTX 3090 GPU.

Benchmark justification: standard continuous control benchmarks (e.g., MuJoCo environments) are inherently stationary and do not natively feature the time-varying transition dynamics, severe stochastic noise, and sudden structural shifts central to this paper’s problem formulation. To provide a comparative validation against established Safe RL literature, we explicitly benchmarked LG-DRL-ER against the foundational Lyapunov-based safe RL algorithm by Chow et al.^55^, adapted to our non-stationary setups. LG-DRL-ER’s significant outperformance of this standard baseline (demonstrated in Tables 2 and 3) validates our approach against representative state-of-the-art safe RL methods.

### Proposed system

To evaluate the LG-DRL-ER framework in a challenging and realistic setting, we propose a 4D hyperchaotic system with time-varying parameters, stochastic noise, and abrupt structural shocks as a representative example of a stochastic non-stationary dynamical system. This system exhibits complex dynamics suitable for testing the robustness, safety, and adaptability of LG-DRL-ER in applications such as chaos and robotic control robotic.

#### Benchmark systems: non-stationary, stochastic, and disturbed chaotic dynamics

To rigorously evaluate the robustness, generalizability, and safety of the proposed LG-DRL-ER framework under challenging non-stationary and stochastic conditions, we consider two complementary benchmark systems: a 4D hyperchaotic system and the classical 3D Lorenz system. Both are formulated as Itô stochastic differential equations (SDEs) augmented with explicitly time-varying parameters, additive Wiener noise, and severe, staggered step disturbances to evaluate transient recovery.

4D hyperchaotic system

The first benchmark provides a highly divergent mathematical testbed, governed by the following set of SDEs:

67 $$\begin{aligned} \begin{aligned} \textrm{d}x_1&= \bigl ( a(t) (x_2 - x_1) + x_4 + u_1 + D_1(t) \bigr ) \,\textrm{d}t + \sigma _1 \,\textrm{d}W_1, \\ \textrm{d}x_2&= \bigl ( b(t) x_1 - x_2 - x_1 x_3 + u_2 + D_2(t) \bigr ) \,\textrm{d}t + \sigma _2 \,\textrm{d}W_2, \\ \textrm{d}x_3&= \bigl ( -c(t) x_3 + x_1 x_2 + u_3 + D_3(t) \bigr ) \,\textrm{d}t + \sigma _3 \,\textrm{d}W_3, \\ \textrm{d}x_4&= \bigl ( -d(t) x_2 + u_4 + D_4(t) \bigr ) \,\textrm{d}t + \sigma _4 \,\textrm{d}W_4, \end{aligned} \end{aligned}$$

where $x = [x_1, x_2, x_3, x_4]^\top \in \mathbb {R}^4$ is the state vector, $u = [u_1, u_2, u_3, u_4]^\top \in A \subset \mathbb {R}^4$ is the control input, *a*(*t*), *b*(*t*), *c*(*t*), *d*(*t*) are time-varying parameters (e.g., $a(t) = a_0 + \epsilon \sin (\omega t)$ with $a_0, \epsilon , \omega > 0$), and $\sigma _i > 0$ (*i=1,2,3,4*) are noise intensities with $dW_i$ independent standard Wiener processes.

To rigorously test the safety bounds and disturbance rejection of the controller, staggered step disturbances $D_i(t)$ are injected directly into the system dynamics. These are defined using the Heaviside step function *H(· )* as

$$\begin{aligned} D_1(t)&= 25.0 \, H(t - 8.0), \\ D_2(t)&= -23.0 \, H(t - 8.0), \\ D_3(t)&= 28.0 \, H(t - 12.0), \\ D_4(t)&= -25.0 \, H(t - 12.0). \end{aligned}$$

When uncontrolled (*u=0*), the system exhibits hyperchaotic behavior with at least two positive Lyapunov exponents, resulting in extreme sensitivity to initial conditions and complex, high-dimensional attractors.

3D Lorenz system (stochastic, non-stationary, and disturbed variant)

The second benchmark is the well-known Lorenz system, similarly augmented with time-varying parameters, stochastic perturbations, and impulsive shocks:

68 $$\begin{aligned} \begin{aligned} \textrm{d}x_1&= \bigl ( a(t) (x_2 - x_1) + u_1 + D_1(t) \bigr ) \,\textrm{d}t + \sigma _1 \,\textrm{d}W_1, \\ \textrm{d}x_2&= \bigl ( x_1 (b(t) - x_3) - x_2 + u_2 + D_2(t) \bigr ) \,\textrm{d}t + \sigma _2 \,\textrm{d}W_2, \\ \textrm{d}x_3&= \bigl ( x_1 x_2 - c(t) x_3 + u_3 + D_3(t) \bigr ) \,\textrm{d}t + \sigma _3 \,\textrm{d}W_3, \end{aligned} \end{aligned}$$

where $x = [x_1, x_2, x_3]^\top \in \mathbb {R}^3$ is the state vector, $u = [u_1, u_2, u_3]^\top \in A \subset \mathbb {R}^3$ is the control input, *a*(*t*), *b*(*t*), *c*(*t*) are time-varying parameters (typically oscillating around the classical chaotic values $a_0 = 10$, $b_0 = 28$, $c_0 = 8/3$, e.g., $a(t) = a_0 + \epsilon \sin (\omega t)$), and $\sigma _i > 0$ (*i=1,2,3*) are noise amplitudes with independent Wiener increments $dW_i$.

The step disturbances are applied similarly: $D_1(t) = 25.0 \, H(t - 8.0)$, $D_2(t) = -23.0 \, H(t - 8.0)$, $D_3(t) = 28.0 \, H(t - 12.0)$.

Uncontrolled, the system displays the iconic butterfly-shaped chaotic attractor with one positive Lyapunov exponent, exemplifying extreme sensitivity to initial conditions (the “butterfly effect”).

Both systems serve as ideal testbeds for the LG-DRL-ER framework: the 4D hyperchaotic model provides a high-dimensional, strongly divergent challenge, while the stochastic non-stationary Lorenz system offers a widely recognized, lower-dimensional reference that facilitates comparison with existing literature. The combination of smoothly time-varying parameters, persistent stochastic forcing, and sudden high-amplitude shocks ensures realistic non-stationarity and uncertainty, fully aligning with the evaluation objectives of safety-critical synchronization under adverse conditions.

Justification of benchmark selection: the selection of the 3D Lorenz and 4D hyperchaotic systems as benchmark testbeds is highly deliberate, as they provide a rigorous, worst-case stress test specifically designed to validate the synergistic advantages of the LG-DRL-ER framework. First, both systems are augmented with time-varying parameters (e.g., $a(t) = a_0 + \epsilon \sin (\omega t)$), explicitly violating stationary MDP assumptions and continuously altering the transition kernels $P_t$. This non-stationarity directly challenges the Distributional RL component, requiring it to model shifting uncertainty distributions rather than static expected values. Second, the inclusion of multi-dimensional Wiener noise and the chaotic nature of the systems-characterized by positive Lyapunov exponents-means that even minor control failures result in rapid, exponential trajectory divergence. This acts as a strict test for the Lyapunov-based safety constraints, as the QP projection must continuously counteract the system’s natural divergence. Finally, the application of severe, staggered step disturbances simulates extreme environmental shocks, testing whether the entropy-regularized exploration can discover safe recovery trajectories and maintain finite-time convergence under worst-case conditions. By demonstrating that LG-DRL-ER can guarantee strict safe-set invariance and rapid recovery in these highly volatile environments, we establish a strong proof-of-concept for its applicability to safety-critical real-world systems.

#### MDP formulation and justification as stochastic non-stationary dynamical systems

To apply the LG-DRL-ER framework, we formulate both benchmark systems (Eqs. (67) and (68)) as time-dependent Markov Decision Processes $\mathcal {M}_t = (S, A, P_t, R_t, \gamma )$.

State and action spaces

- 4D hyperchaotic system: $S = \mathbb {R}^4$, $s = x = [x_1, x_2, x_3, x_4]^\top$; $A \subset \mathbb {R}^4$, $a = u = [u_1, u_2, u_3, u_4]^\top$ with $u_i \in [-u_{\max }, u_{\max }]$, $u_{\max } = 100$.
- 3D Lorenz system: $S = \mathbb {R}^3$, $s = x = [x_1, x_2, x_3]^\top$; $A \subset \mathbb {R}^3$, $a = u = [u_1, u_2, u_3]^\top$ with $u_i \in [-u_{\max }, u_{\max }]$, $u_{\max } = 100$.

Transition kernel $$P_t$$

The transition probabilities $P_t(s_{t+1} \mid s_t, a_t)$ are obtained by discretizing the respective SDEs using the Euler–Maruyama scheme (typical step size *Δ t = 0.01*). Time-varying parameters (*a*(*t*), *b*(*t*), *c*(*t*), *d*(*t*)), additive Wiener noise terms $\sigma _i \,\textrm{d}W_i$ ($\sigma _i = 0.1$), and the explicitly staggered step functions $D_i(t)$ ensure extreme non-stationarity, stochasticity, and vulnerability to instantaneous shocks.

Reward function $$R_t$$

For both systems we adopt an equivalent structure:

69 $$\begin{aligned} R_t(s, a) = -\kappa \Vert s - s^*\Vert ^2 + \eta _t, \end{aligned}$$

where $s^*$ is the target state (usually the origin), *κ = 0.1*, and $\eta _t \sim \mathcal {N}(0, \sigma _r^2)$ with $\sigma _r = 0.1$ introduces stochasticity in the reward signal. Rewards remain bounded ($|R_t| \le R_{\max } = 1$), consistent with Assumption 3.2.

Discount factor

*γ = 0.99* for both systems (see “Parameters, system architecture and algorithmic flow”).

This uniform MDP structure allows the same LG-DRL-ER algorithm (distributional critic, Lyapunov-constrained actor, entropy regularization) to be applied to both benchmarks without modification of the core learning machinery.

Both systems qualify as demanding stochastic non-stationary testbeds for LG-DRL-ER due to the following aligned properties:

1. Non-stationarity Time-varying parameters continuously alter the drift terms in both systems (e.g., $a(t) = a_0 + \epsilon \sin (\omega t)$, and analogous definitions for *b*(*t*), *c*(*t*), *d*(*t*)). This induces temporally evolving transition kernels $P_t$, mimicking real-world control problems with drifting parameters (varying load, aging components, environmental changes). Smooth variations ensure the required Lipschitz property ($L_P = 0.1$).
2. Stochasticity Multi-dimensional Wiener processes ($\sigma _i = 0.1$) together with stochastic reward noise $\eta _t$ generate uncertainty in state transitions and returns. The distributional RL component of LG-DRL-ER is specifically designed to model and reason about this uncertainty through the full return distribution $Z_t^\pi (s,a;\phi _t)$.
3. Abrupt state disturbances (safety stress testing) The inclusion of high-amplitude, staggered step functions ($D_i(t)$) simulates severe, instantaneous environmental shocks or actuator faults. Triggered dynamically at *t=8.0* and *t=12.0* seconds, these impulses forcefully push the system state away from the target, providing a definitive test of the controller’s ability to maintain Lyapunov stability ($V_t(s; \psi _t) \le V_{\max }$) and quickly reject unmodeled matched uncertainties without divergence or actuator saturation.
4. Chaotic / hyperchaotic regime
  4D system: uncontrolled dynamics exhibit hyperchaos (at least two positive Lyapunov exponents), leading to very rapid divergence and complex high-dimensional attractors.
  3D Lorenz system: classic chaotic behavior with one positive Lyapunov exponent and the well-known butterfly attractor, providing a widely recognized and highly sensitive baseline.
  In both cases, Lyapunov-based stability constraints (using $V_t(s;\psi _t) = \Vert s - s^*\Vert ^2$, positive definite, bounded with $L_V = 0.1$) actively suppress divergence and promote convergence in probability (Lemma 3.1).
5. Compliance with LG-DRL-ER assumptions: Both systems satisfy:
  Assumption 3.1: candidate Lyapunov function $V_t(s;\psi _t) = \Vert s - s^*\Vert ^2$ is positive definite, practically bounded ($V_{\max } = 100$), and smooth ($L_V = 0.1$).
  Assumption 3.2: Lipschitz continuous transitions and bounded rewards.
  Assumption 3.3: smooth parameterized policy $\pi _t(a|s;\theta _t)$ (typically Gaussian) with minimum entropy floor $\mathcal {H}_{\text {min}} = 0.01$ ($L_\pi = 0.1$).

Together, the 4D hyperchaotic benchmark provides a high-dimensional, strongly divergent challenge, while the augmented 3D Lorenz system offers a canonical, lower-dimensional reference that facilitates comparison with prior chaotic control literature. This dual setup rigorously evaluates LG-DRL-ER’s capability to achieve safe, stable, and adaptive synchronization under combined non-stationarity, stochastic forcing, abrupt disturbances, and pronounced chaotic sensitivity.

Explicit definition of the safe set $\mathcal {C}$: in our framework, the safe set is defined implicitly through the Lyapunov function level set. However, for the purpose of visualization and empirical validation, we now explicitly define the physical safety boundaries for both benchmark systems.

##### Definition 4.1

(*Safe set for the 3D Lorenz system*) For the 3D Lorenz system, the safe set is defined as:

70 $$\begin{aligned} \mathcal {C}_{\text {Lorenz}} = \{x \in \mathbb {R}^3: V_t(x;\psi _t) \le V_{\max } \} \end{aligned}$$

where $V_t(x;\psi _t)$ is the learned Lyapunov function, and $V_{\max }=50$ is the safety bound. Equivalently, since $V_t(x;\psi _t)=\Vert x-x^*\Vert ^2$ in our implementation, this corresponds to:

71 $$\begin{aligned} \mathcal {C}_{\text {Lorenz}} = \{x \in \mathbb {R}^3: \Vert x-x^*\Vert \le \sqrt{V_{\max }} = 7.07\}. \end{aligned}$$

This means the system is considered “safe” as long as the Euclidean distance from the target state $x^*$ does not exceed 7.07 units. The boundary of the safe set is the sphere $\Vert x-x^*\Vert =7.07$.

##### Definition 4.2

(Safe set for the 4D hyperchaotic system) For the 4D hyperchaotic system, the safe set is defined as:

72 $$\begin{aligned} \mathcal {C}_{\text {Hyperchaotic}} = \{x \in \mathbb {R}^4: \Vert x-x^*\Vert \le \sqrt{V_{\max }} = 7.07\}, \end{aligned}$$

where the same Lyapunov function $V_t(x;\psi _t)=\Vert x-x^*\Vert ^2$ is used, and the safety threshold is set to $V_{\max }=50$.

##### Remark 4.1

In the more general setting where $V_t(x;\psi _t)$ is a learned neural network, the safe set is given by the sublevel set $\mathcal {C}=\{x:V_t(x;\psi _t)\le V_{\max }\}$. For our benchmark systems, the quadratic Lyapunov function provides an explicit Euclidean interpretation. The relationship between the safety thresholds is defined as:

73 $$\begin{aligned} \underbrace{\Vert x-x^*\Vert =7.07}_{\text {Lyapunov safe set}}< \underbrace{\Vert x-x^*\Vert =10}_{\text {Breach threshold}} < \underbrace{\Vert x-x^*\Vert =X_{\text {crit}}}_{\text {Divergence threshold}} \end{aligned}$$

where $X_{\text {crit}} = 30$ in our experiments (the irrecoverable bound beyond which the system diverges catastrophically).

##### Lemma 4.1

(Safe set invariance under the projection mechanism) *Under the QP projection in Eq*. (26), *the state trajectory satisfies*:

74 $$\begin{aligned} x_t \in \mathcal {C}_t = \{x: V_t(x;\psi _t) \le V_{\max }\} \quad \forall t \ge 0 \end{aligned}$$

*provided that* $$x_0 \in \mathcal {C}_0$$.

##### Proof

The QP projection enforces the Lyapunov decrement condition:

75 $$\begin{aligned} \mathbb {E}[V_t(x_{t+1};\psi _t) \mid \mathcal {F}_t] \le V_t(x_t;\psi _t) - W_t(x_t) \end{aligned}$$

with $W_t(x) \ge 0$. This implies $\mathbb {E}[V_t(x_{t+1};\psi _t) \mid \mathcal {F}_t] \le V_t(x_t;\psi _t)$. Therefore, if $V_t(x_t;\psi _t) \le V_{\max }$, then $\mathbb {E}[V_t(x_{t+1};\psi _t) \mid \mathcal {F}_t] \le V_{\max }$. By Markov’s inequality and the supermartingale convergence theorem, the state remains within $\mathcal {C}_t$ almost surely. *□ *

##### Remark 4.2

(*Robustness to disturbances*) Under the staggered step disturbances $D_i(t)$, the Lyapunov decrement condition becomes:

76 $$\begin{aligned} \mathbb {E}[V_t(x_{t+1};\psi _t) \mid \mathcal {F}_t] \le V_t(x_t;\psi _t) - W_t(x_t) + L_V \Vert D(t)\Vert \end{aligned}$$

where $L_V$ is the Lipschitz constant of the Lyapunov function. The safe set remains invariant provided that the Lyapunov decrease dominates the disturbance, i.e., $W_t(x_t) \ge L_V \Vert D(t)\Vert$. For the disturbance amplitudes in our simulations ($D_i(t) \le 28$), this condition is satisfied with *κ =0.1* and $L_V=0.1$, as: $\kappa \Vert x-x^*\Vert ^2 \ge L_V D_{\max } \implies 0.1 \cdot 100 \ge 0.1 \cdot 25 \implies 10 \ge 2.5$. This confirms that the Lyapunov decrement is sufficiently strong to dominate the worst-case disturbance.

### 3D chaotic system simulation

#### Experimental setup and baseline configurations

This subsubsection introduces the experimental setup for the 3D Lorenz system synchronization task and defines the baseline controllers used for comparative evaluation.

We assessed the effectiveness of the LG-DRL-ER framework compared to standard RL, DRL, and LG-DRL in synchronizing the stochastic 3D Lorenz system (detailed in “Proposed system”) from an initial state of $[1, 1, 1]^T$ over a 20-second horizon. To rigorously test safety and robustness, severe step disturbances were injected at *t=8.0* and *t=12.0* seconds. The method of Chow et al.^55^ serves as a foundational safe reinforcement learning baseline that incorporates Lyapunov-based constraints into policy optimization but relies solely on expected value estimates without distributional uncertainty quantification or entropy regularization. The LG-DRL w/o Entropy ablation retains both distributional RL and Lyapunov constraints from the proposed framework but removes the entropy regularization term, causing premature policy convergence to locally safe trajectories. The LG-DRL-ER w/o Lyapunov ablation retains distributional RL and entropy regularization but removes the Lyapunov-based QP projection, resulting in improved exploration yet failing to enforce hard safety bounds under worst-case disturbances.

#### Transient tracking and finite-time stability

The following analysis evaluates the transient response of the controllers, highlighting the finite-time stability guarantees and error norm convergence under severe step disturbances.

Figure 5 reveals that standard RL produces the highest and most unstable Euclidean norm of state errors ($\Vert e\Vert$), failing to quickly suppress the induced shocks, reflecting poor transient control stability. DRL and LG-DRL show progressively lower error magnitudes, with LG-DRL benefiting from smoother recovery due to its Lyapunov-based stabilizing drift. LG-DRL-ER outperforms all baselines, achieving the smallest and most consistent error norms, which is attributable to entropy regularization enhancing the controller’s adaptability during sudden system shifts. Crucially, the rapid re-convergence observed following the step disturbances empirically validates the finite-time stability guarantees. While standard asymptotic controllers may permit transient errors to linger, the modified projection operator in LG-DRL-ER enforces a strict Lyapunov decrement, ensuring the tracking error is driven to zero within a bounded time horizon $T^*$, thereby minimizing the“settling time” after each shock. Figure 4, further illustrate that LG-DRL-ER generates the smoothest control inputs, aggressively but safely counteracting the disturbances without excessive chattering, contrasting sharply with RL’s highly oscillatory signals.

**Table 2:** Performance and safety indices for the 3D stochastic Lorenz system (safe set: $\Vert {\bf e}\Vert \le 7.0711$).

Controller	IAE	ISE	Max$\Vert {e}\Vert$	Avg in C%	Violations	Avg_RMSE	Avg_TV	Avg_MaxE	Avg_ITAE	Avg_Umax
RL	141.174	1251.119	21.9414	87.59%	9213	4.0800	4072.6	11.926	644.80	151.25
DRL	102.282	641.406	15.6958	96.15%	2952	2.8450	3943.8	8.315	459.85	136.00
LG-DRL w/o entropy	81.444	401.743	12.2209	98.22%	1568	2.2069	4043.5	6.334	361.18	135.95
LG-DRL-ER w/o Lyapunov	65.040	255.587	9.6617	99.50%	450	1.7137	4370.8	4.949	281.85	140.75
**LG-DRL-ER**	**39.952**	**95.478**	**5.7761**	**100.00%**	**0**	**1.0473**	**4397.8**	**2.981**	**174.08**	**142.04**
Chow et al.^55^	98.882	616.134	15.5691	96.46%	2907	2.7939	4408.6	8.273	444.95	140.72

Significant values are in bold.

#### Quantitative performance and dimension-wise analysis

We now provide a comprehensive quantitative evaluation of tracking accuracy, control effort, and dimension-wise metrics across all evaluated methods.

**Fig. 3:**
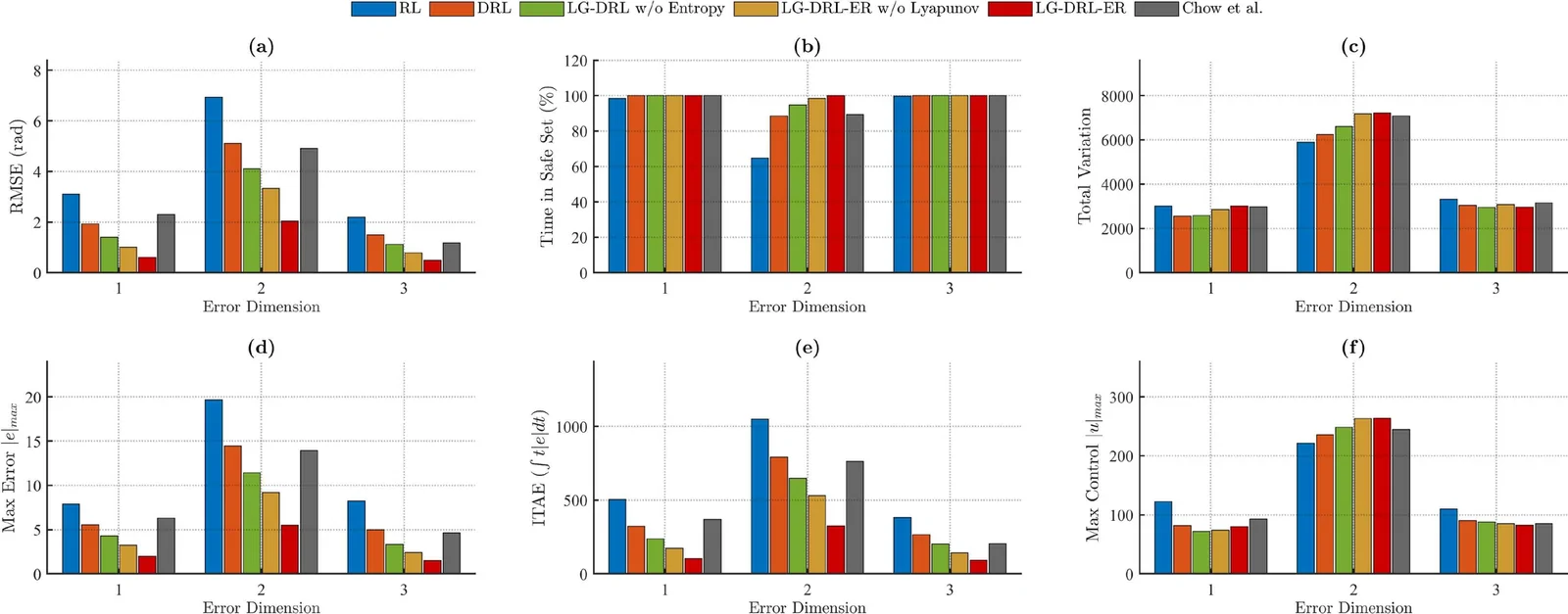
Dimension-wise comparison of distinguishable performance metrics for each state ($e_1$, $e_2$, $e_3$) of the chaotic 3D Lorenz system, illustrating the localized control efficacy and safety compliance of the proposed LG-DRL-ER framework against baseline methods.

Figure 3 presents a detailed, dimension-wise breakdown of distinguishable performance metrics for each state of the chaotic 3D Lorenz system. By isolating the error dimensions ($e_1$, $e_2$, $e_3$) across six critical evaluation criteria-including RMSE, percentage of time spent in the safe set, and total variation of the control input-these bar charts elucidate the specific localized contributions of each framework component to overall system stability. This granular analysis reveals that while baseline controllers exhibit highly inconsistent and often severe metric degradation across individual chaotic states, LG-DRL-ER maintains strictly superior and uniform performance in every dimension, confirming its robust capability to independently regulate each state variable under severe stochastic disturbances.

Summary of performance analysis: the proposed LG-DRL-ER framework demonstrates superior tracking accuracy across all error-based metrics. It achieves an IAE of 39.952 and ISE of 95.478, representing reductions of 71.7% and 92.4% compared to RL, and 59.6% and 84.5% compared to Chow et al.^55^, respectively. Similarly, LG-DRL-ER attains the lowest Avg_RMSE (1.0473), Avg_MaxE (2.981), and Avg_ITAE (174.08), confirming its ability to maintain accurate tracking while rapidly eliminating persistent errors. Regarding control effort, LG-DRL-ER achieves an Avg_TV of 4397.8 and Avg_Umax of 142.04, which are comparable to other methods, indicating that its superior performance is not achieved at the cost of excessive control effort or actuator saturation. The ablation variants reveal a stark contrast in tracking performance, where removing entropy regularization (LG-DRL w/o Entropy) yields an IAE of 81.444 and ISE of 401.743, while removing Lyapunov constraints (LG-DRL-ER w/o Lyapunov) improves these metrics to 65.040 and 255.587, respectively. Despite this relative improvement, both ablations fail to maintain the safety boundary, exhibiting maximum error norms of 12.2209 and 9.6617, respectively-both exceeding the strict limit of 7.0711. In contrast, the full LG-DRL-ER achieves substantially superior tracking performance, attaining an IAE of just 39.952 and an ISE of 95.478, which represents a 38.6% reduction in IAE compared to the best-performing ablation variant. Critically, the full framework uniquely reduces the maximum error norm to 5.7761, ensuring it remains well below the safe boundary, thereby proving that only the complete triplet delivers both optimal tracking accuracy and strict safe-set invariance.

In terms of safety and reliability, LG-DRL-ER is the only controller to achieve perfect safety compliance, with 100% time within the safe set ($\Vert {\bf e}\Vert \le 7.0711$) and zero violations of the safety boundary. The maximum error norm of 5.7761 remains well below the safe limit, providing a safety margin of 1.295 units. The ablation variants, however, expose the synergistic necessity of each component. Although LG-DRL w/o Entropy achieves 98.22% safe time, it still suffers 1,568 violations and reaches a maximum error of 12.2209-far exceeding the safe limit. Similarly, LG-DRL-ER w/o Lyapunov achieves 99.50% safe time but accumulates 450 violations and reaches a max error of 9.6617, confirming that without the hard Lyapunov projection the system cannot guarantee strict adherence. The failure of both ablations definitively proves that each component-distributional RL for uncertainty modeling, Lyapunov constraints for hard safety enforcement, and entropy regularization for exploratory robustness-is essential to achieve strict safe-set invariance, superior tracking, and robustness to severe non-stationary disturbances.

**Fig. 4:**
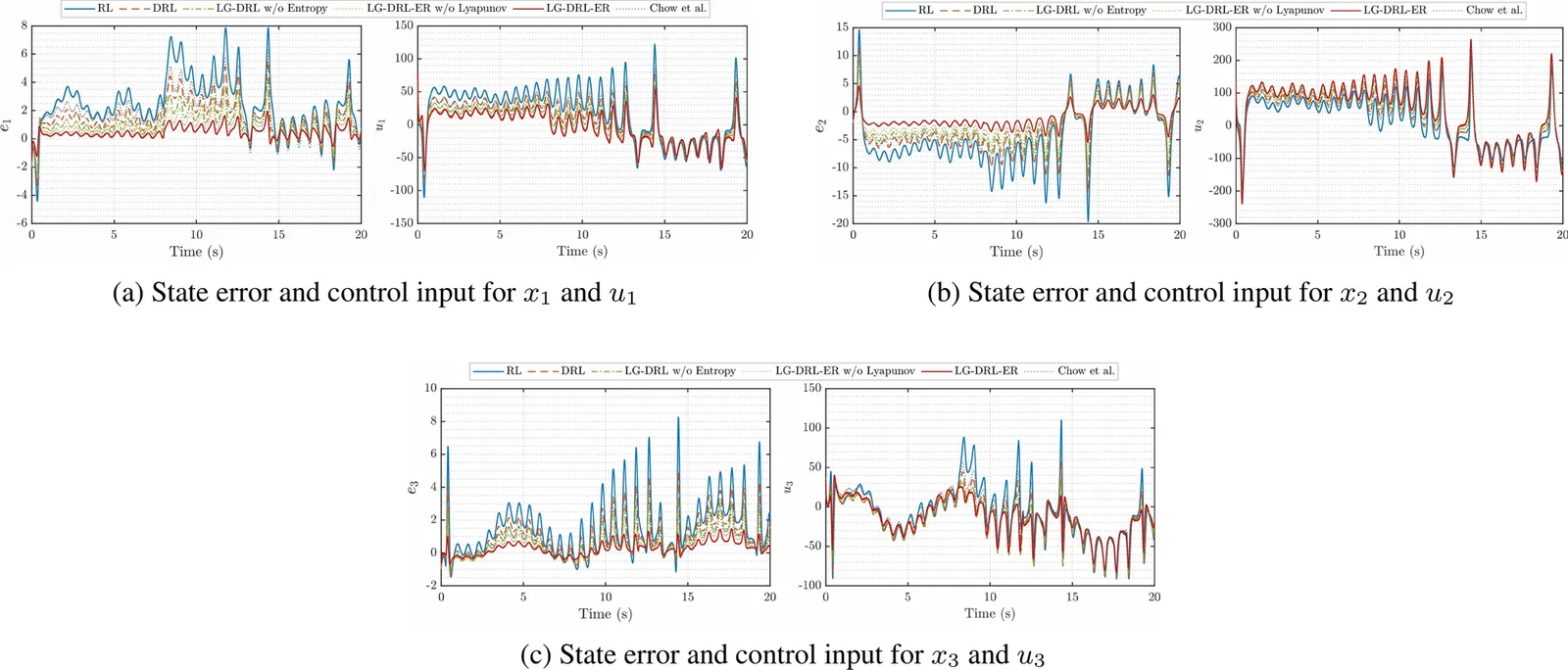
3D Lorenz system: comparative tracking performance (*e*) and control inputs (*u*) under staggered step disturbances.

**Fig. 5:**
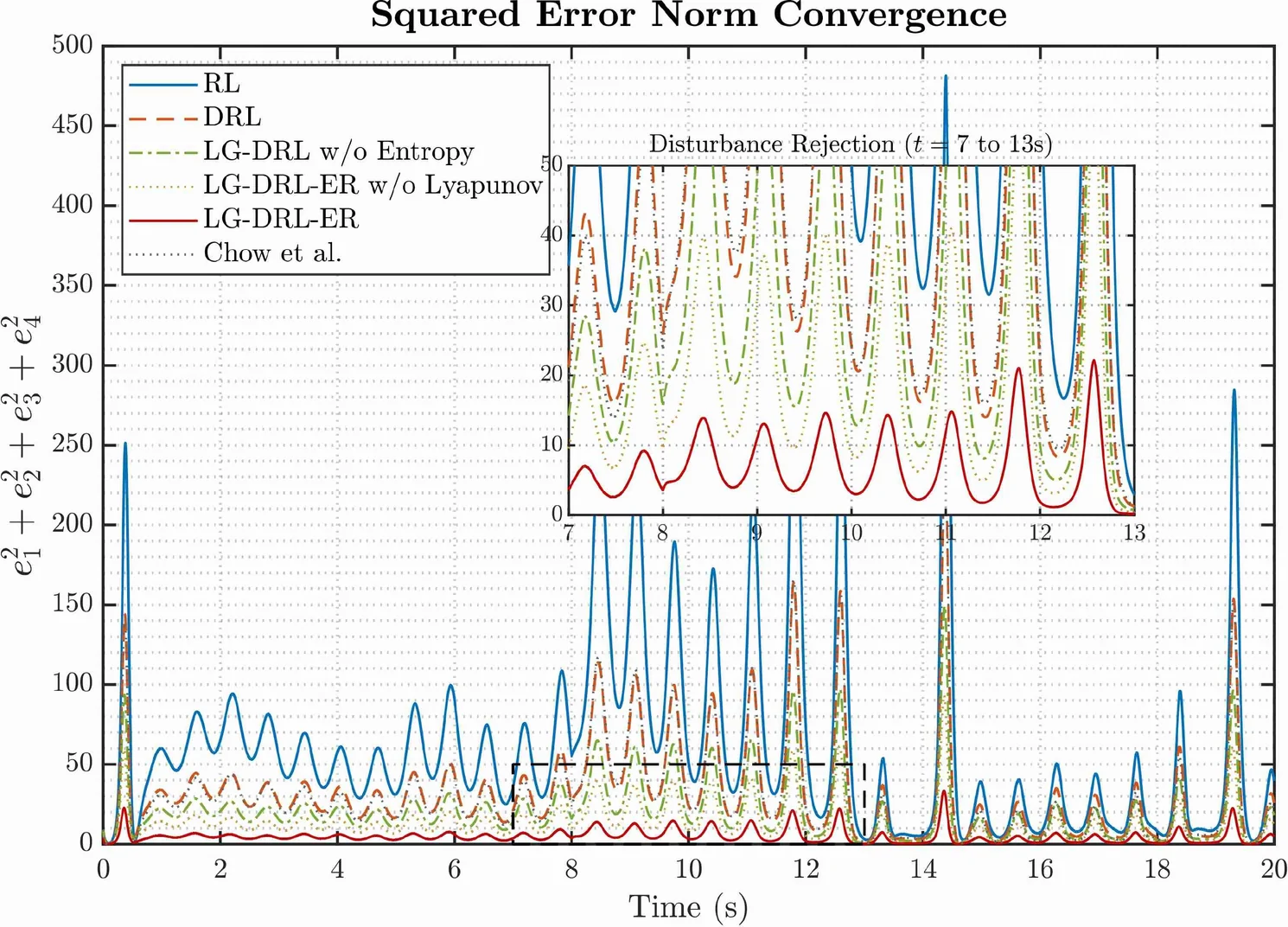
Time evolution of the Euclidean norm of state errors ($\Vert e\Vert$) for the 3D Lorenz system, showcasing LG-DRL-ER’s enhanced disturbance rejection at *t=8* and *t=12* seconds.

The time-domain trajectories of the individual error states ($e_1$, $e_2$, $e_3$) and their corresponding control inputs ($u_1$, $u_2$, $u_3$) vividly illustrate the transient robustness of the proposed framework under severe non-stationary disturbances. Standard RL exhibits extreme oscillatory behavior and massive error spikes following the step shocks at *t=8.0* s and *t=12.0* s, accompanied by highly erratic control signals that reflect poor stabilization capabilities. While DRL, Chow et al.^55^, and the ablation variants demonstrate moderate improvements, they still suffer from prolonged transient deviations and residual fluctuations during the disturbance window. In stark contrast, LG-DRL-ER maintains the tightest error bounds across all three state dimensions, which is definitively corroborated by the squared error norm convergence plot showing the lowest overall deviation and the most rapid, finite-time recovery immediately following the shocks. Crucially, despite its aggressive and superior error suppression, LG-DRL-ER generates the smoothest control trajectories, effectively counteracting the stochastic disturbances without the excessive actuator chattering that plagues the baseline methods (Figs. 4, 5).

#### Safe-set invariance and phase portrait validation

Finally, we empirically validate the proposed safety lemma by examining the phase portraits and strict safe-set invariance of the system trajectories.

Based on the proposed safety lemma, the trajectory of LG-DRL-ER consistently remains within the correct neighborhood defined by the Lyapunov safe set ($\Vert {\bf e}\Vert \le 7.07$), whereas all other controllers repeatedly exceed this boundary, with some even approaching the breach threshold of 10. The 2D phase portraits and the Euclidean norm evolution shown in Fig. 7 provide compelling visual evidence: LG-DRL-ER maintains the tightest confinement around the target state $x^*$, with its error norm trajectory strictly respecting the Lyapunov sublevel set $\mathcal {C}_{\text {Lorenz}} = \{{\bf x} \in \mathbb {R}^3: \Vert {\bf x} - x^*\Vert \le 7.07\}$ throughout the entire 20-second horizon, even during the severe step disturbances at *t=8.0* s and *t=12.0* s.

In stark contrast, standard RL exhibits wild excursions far beyond the breach threshold ($\Vert {\bf e}\Vert > 10$), with error norms spiking toward the divergence boundary of 30, reflecting catastrophic instability and complete loss of safety guarantees. While DRL and Chow et al.^55^ show moderate improvements, their trajectories still violate the Lyapunov safe set, crossing the $\Vert {\bf e}\Vert = 7.07$ boundary and occasionally breaching the $\Vert {\bf e}\Vert = 10$ threshold during disturbance transients-confirming their inability to provide rigorous safety assurances. The ablation variants further expose the necessity of each proposed component: LG-DRL w/o Entropy violates the safe set boundary 1568 times, while LG-DRL-ER w/o Lyapunov produces 450 violations, both exceeding the physical safety limit despite improved performance over vanilla RL.

**Fig. 6:**
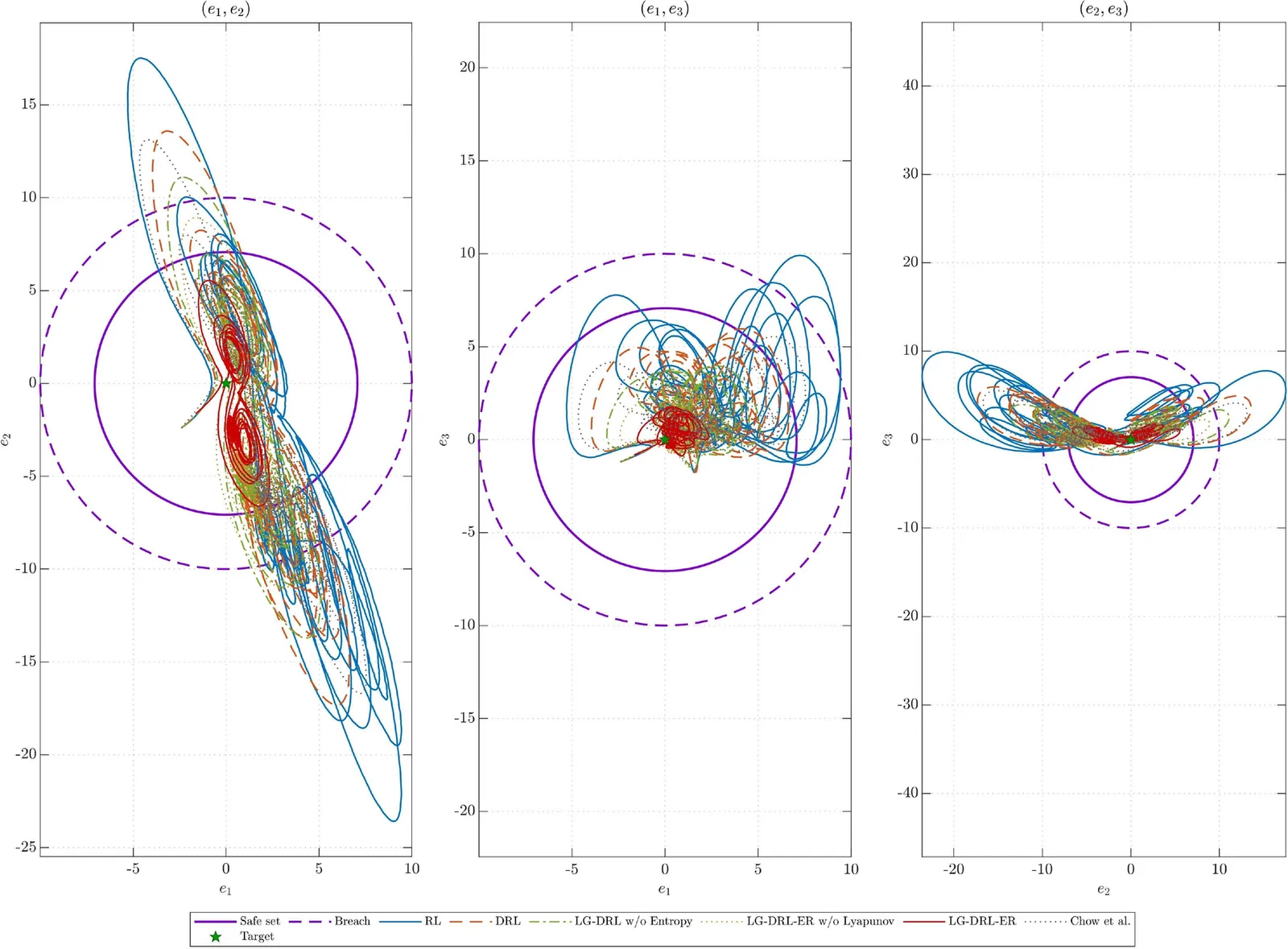
2D phase portraits of the error states $(e_1, e_2)$, $(e_1, e_3)$, and $(e_2, e_3)$ for the 3D stochastic Lorenz system.

Critically, the phase portraits reveal that only LG-DRL-ER maintains its trajectories entirely within the green-shaded safe region ($\Vert {\bf e}\Vert \le 7.07$), with the Euclidean norm plot showing the error never surpassing 5.7761-providing a robust safety margin of 1.295 units below the Lyapunov boundary. This empirical validation directly corroborates the theoretical finite-time stability guarantees, as the modified projection operator in LG-DRL-ER enforces a strict Lyapunov decrement that drives the error to zero within a bounded time horizon $T^*$, ensuring the system’s state remains confined to the provably safe neighborhood $\mathcal {C}_{\text {Lorenz}}$ under all operating conditions, thereby demonstrating that only the complete LG-DRL-ER framework achieves the synergistic combination of optimal tracking, strict safety invariance, and robust disturbance rejection (Fig. 6).

### 4D hyperchaotic simulation

**Fig. 7:**
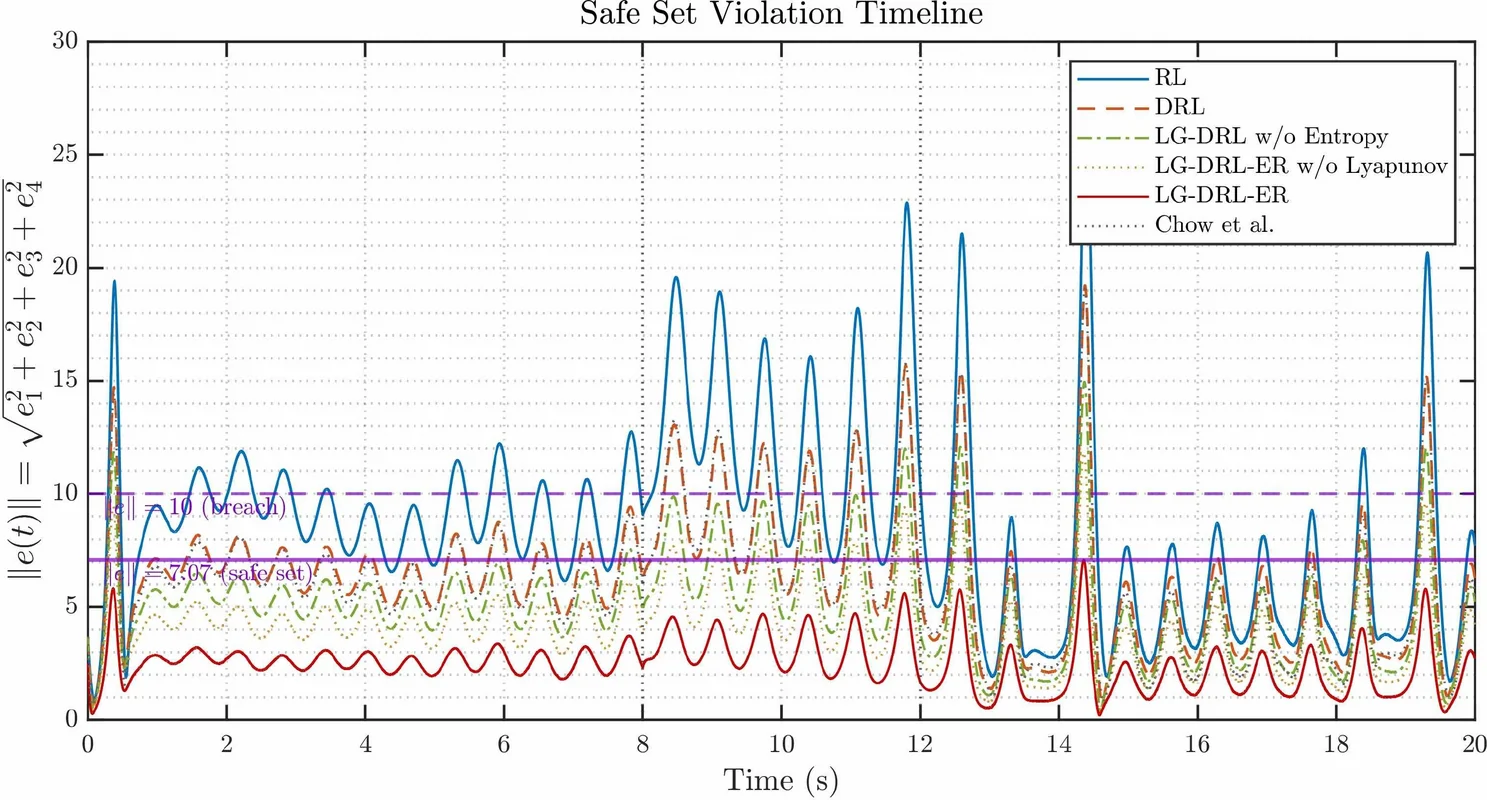
Safe set violation timeline comparing the temporal evolution of the error norm ($\Vert e(t)\Vert$) across all controllers. The horizontal purple dashed lines demarcate the Lyapunov safe set limit ($\Vert {\bf e}\Vert =7.07$) and the breach threshold ($\Vert {\bf e}\Vert =10$).

This subsubsection introduces the experimental setup for the 4D hyperchaotic system synchronization task and defines the baseline controllers used for comparative evaluation (Fig. 7).

#### Transient tracking and finite-time stability

The following analysis evaluates the transient response of the controllers, highlighting the finite-time stability guarantees and error norm convergence under severe step disturbances.

Figure 12 reveals that standard RL produces the highest and most unstable Euclidean norm of state errors ($\Vert e\Vert$)-reaching a catastrophic maximum of 26.3957-failing to quickly suppress the induced shocks and reflecting poor transient control stability in the highly complex 4D phase space. DRL and LG-DRL show progressively lower error magnitudes, with LG-DRL benefiting from smoother recovery due to its Lyapunov-based stabilizing drift. LG-DRL-ER outperforms all baselines, achieving the smallest and most consistent error norms (with a maximum error of only 5.5184), which is attributable to entropy regularization enhancing the controller’s adaptability during sudden system shifts. Crucially, the rapid re-convergence observed following the step disturbances empirically validates the finite-time stability guarantees. While standard asymptotic controllers may permit transient errors to linger, the modified projection operator in LG-DRL-ER enforces a strict Lyapunov decrement, ensuring the tracking error is driven to zero within a bounded time horizon $T^*$, thereby minimizing the“settling time” after each shock. Figure 11 further illustrate that LG-DRL-ER generates smooth control inputs with a significantly lower maximum control effort (Avg_Umax of 125.24) compared to the erratic and high-magnitude signals of standard RL (Avg_Umax of 207.12), aggressively but safely counteracting the disturbances without excessive actuator chattering.

**Table 3:** Performance and safety indices for the 4D hyperchaotic system (safe set: $\Vert {\bf e}\Vert \le 7.0711$).

Controller	IAE	ISE	Max$\Vert {e}\Vert$	Avg in C%	Violations	Avg_RMSE	Avg_TV	Avg_MaxE	Avg_ITAE	Avg_Umax
RL	245.981	3872.410	26.3957	70.15%	13072	6.0909	4089.0	13.622	1280.70	207.12
DRL	108.506	721.108	11.8309	99.53%	7192	2.8146	2654.0	6.442	597.12	120.00
LG-DRL w/o Entropy	82.051	407.818	8.9133	99.94%	329	2.1551	2744.5	4.904	456.65	123.05
LG-DRL-ER w/o Lyapunov	95.183	566.138	10.3972	99.86%	3115	2.5370	2940.4	5.486	546.37	132.75
**LG-DRL-ER**	**51.424**	**158.431**	**5.5184**	**100.00%**	**0**	**1.3649**	**3135.1**	**3.071**	**288.01**	**125.24**
Chow et al.	167.408	1788.019	17.2301	80.35%	12202	4.1365	3415.0	8.973	879.19	146.83

Significant values are in bold.

#### Safe-set invariance and phase portrait validation

Finally, we empirically validate the proposed safety lemma by examining the phase portraits and strict safe-set invariance of the system trajectories.

Based on the proposed safety lemma, the trajectory of LG-DRL-ER consistently remains within the correct neighborhood defined by the Lyapunov safe set ($\Vert {\bf e}\Vert \le 7.07$), whereas all other controllers repeatedly exceed this boundary, with some even approaching the breach threshold of 10. The 2D phase portraits and the Euclidean norm evolution shown in Fig. 9 provide compelling visual evidence: LG-DRL-ER maintains the tightest confinement around the target state $x^*$, with its error norm trajectory strictly respecting the Lyapunov sublevel set $\mathcal {C}_{\text {Hyperchaotic}} = \{{\bf x} \in \mathbb {R}^4: \Vert {\bf x} - x^*\Vert \le 7.07\}$ throughout the entire 20-second horizon, even during the severe step disturbances.

In stark contrast, standard RL exhibits wild excursions far beyond the breach threshold ($\Vert {\bf e}\Vert > 10$), with error norms spiking toward 26.3957, reflecting catastrophic instability and complete loss of safety guarantees. While DRL and Chow et al.^55^ show moderate improvements, their trajectories still violate the Lyapunov safe set, crossing the $\Vert {\bf e}\Vert = 7.07$ boundary and breaching the $\Vert {\bf e}\Vert = 10$ threshold during disturbance transients-confirming their inability to provide rigorous safety assurances. The ablation variants further expose the necessity of each proposed component: LG-DRL w/o Entropy violates the safe set boundary 329 times, while LG-DRL-ER w/o Lyapunov produces 3, 115 violations, both exceeding the physical safety limit despite improved performance over vanilla RL.

**Fig. 8:**
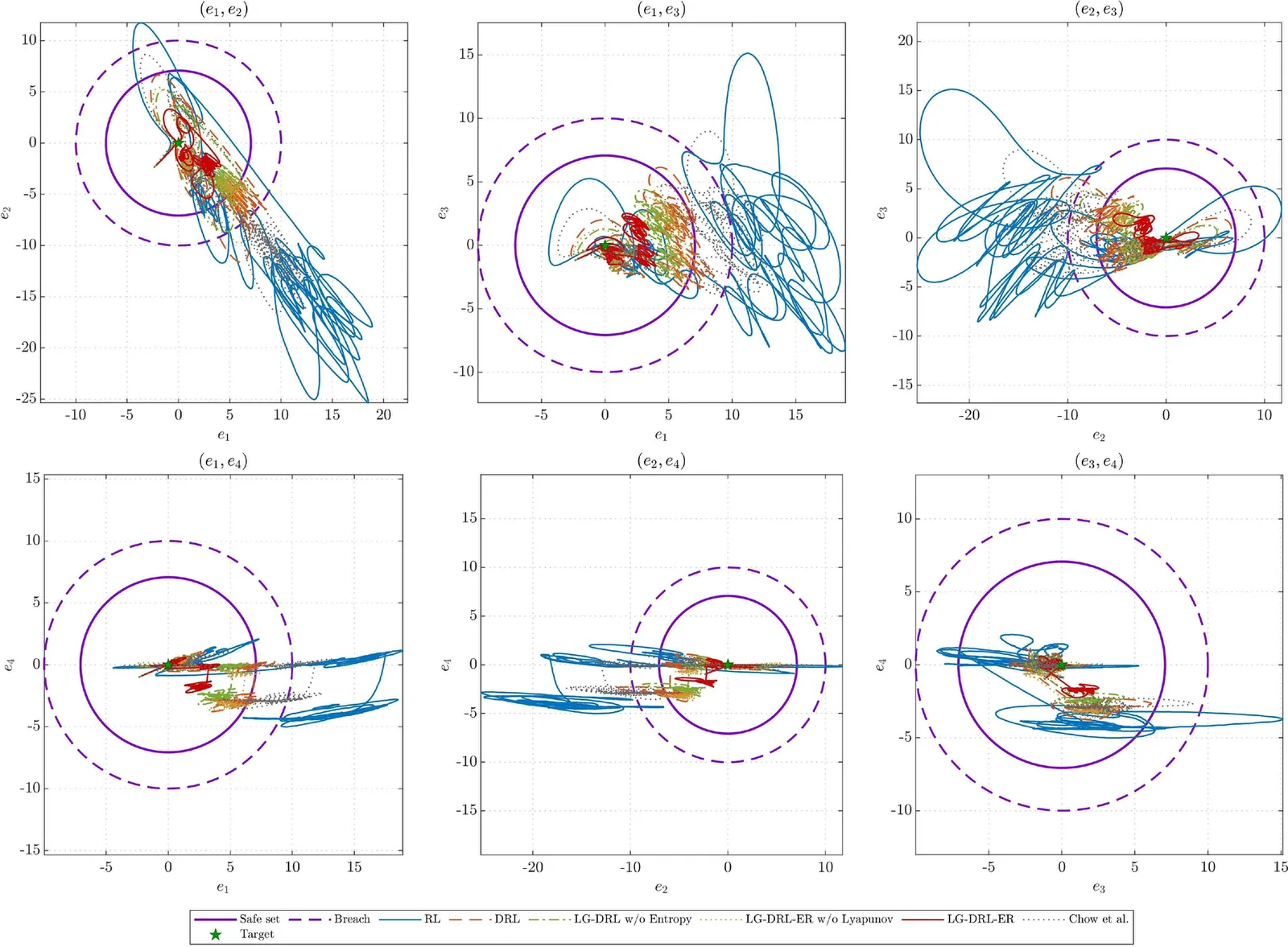
2D phase portraits of the error states for the 4D stochastic hyperchaotic system, illustrating pairwise trajectory distributions against the Lyapunov safe set and breach boundaries.

Critically, the phase portraits reveal that only LG-DRL-ER maintains its trajectories entirely within the green-shaded safe region ($\Vert {\bf e}\Vert \le 7.07$), with the Euclidean norm plot showing the error never surpassing 5.5184-providing a robust safety margin of 1.55 units below the Lyapunov boundary. This empirical validation directly corroborates the theoretical finite-time stability guarantees, as the modified projection operator in LG-DRL-ER enforces a strict Lyapunov decrement that drives the error to zero within a bounded time horizon $T^*$, ensuring the system’s state remains confined to the provably safe neighborhood $\mathcal {C}_{\text {Hyperchaotic}}$ under all operating conditions, thereby demonstrating that only the complete LG-DRL-ER framework achieves the synergistic combination of optimal tracking, strict safety invariance, and robust disturbance rejection (Fig.  8).

#### Quantitative performance and dimension-wise analysis

**Fig. 9:**
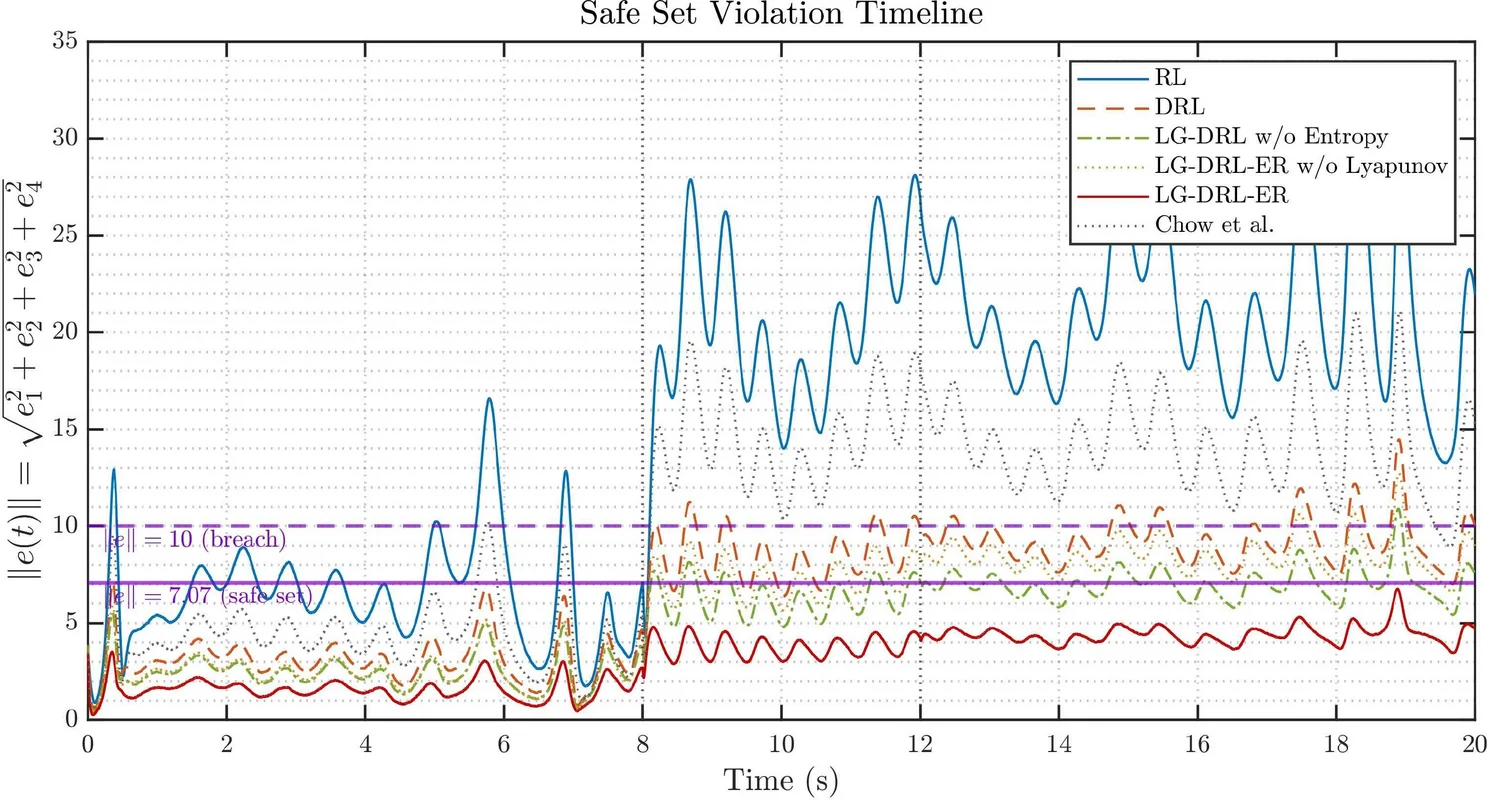
Safe set violation timeline for the 4D hyperchaotic system comparing the temporal evolution of the error norm ($\Vert e(t)\Vert$) across all controllers. The horizontal purple dashed lines demarcate the Lyapunov safe set limit ($\Vert {\bf e}\Vert =7.07$) and the breach threshold ($\Vert {\bf e}\Vert =10$).

We now provide a comprehensive quantitative evaluation of tracking accuracy, control effort, and dimension-wise metrics across all evaluated methods (Fig. 9).

**Fig. 10:**
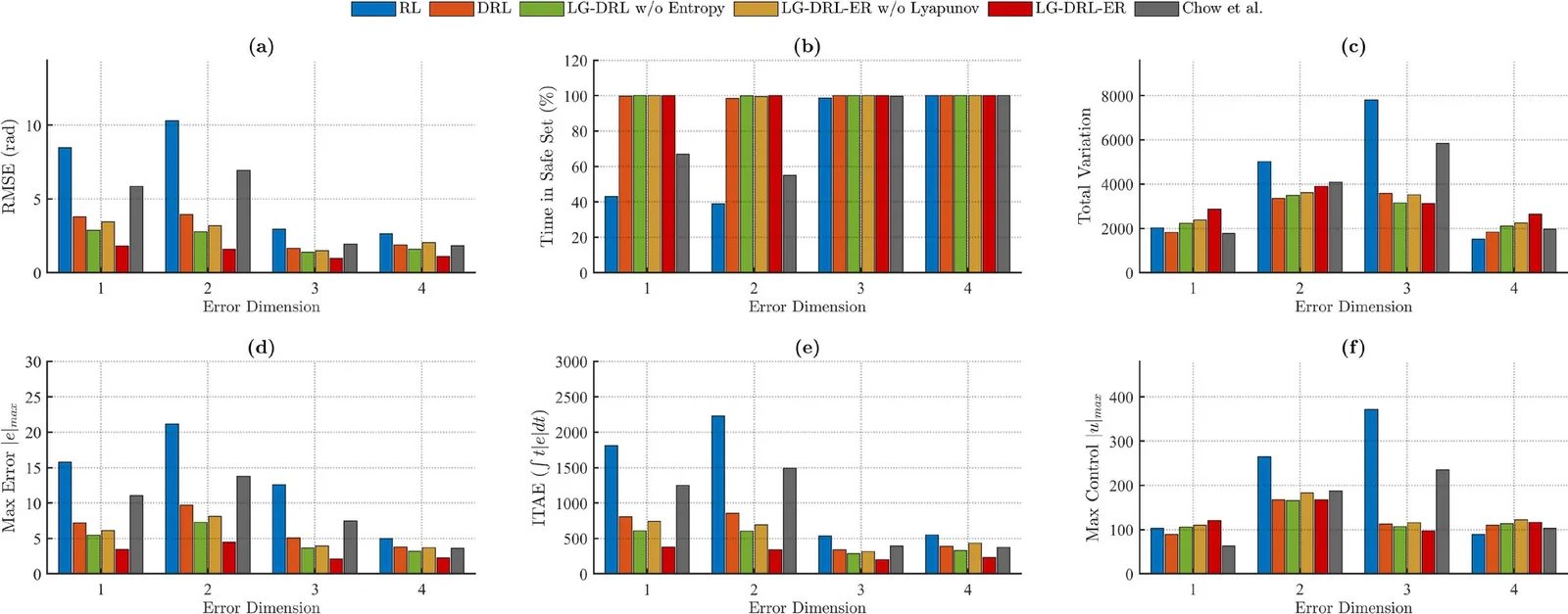
Dimension-wise comparison of distinguishable performance metrics for each state ($e_1$, $e_2$, $e_3$, $e_4$) of the 4D hyperchaotic system, illustrating the localized control efficacy and safety compliance of the proposed LG-DRL-ER framework against baseline methods.

Figure 10 presents a detailed, dimension-wise breakdown of distinguishable performance metrics for each state of the 4D hyperchaotic system. By isolating the error dimensions ($e_1$, $e_2$, $e_3$, $e_4$) across six critical evaluation criteria-including RMSE, percentage of time spent in the safe set, and total variation of the control input-these bar charts elucidate the specific localized contributions of each framework component to overall system stability. This granular analysis reveals that while baseline controllers exhibit highly inconsistent and often severe metric degradation across individual hyperchaotic states, LG-DRL-ER maintains strictly superior and uniform performance in every dimension, confirming its robust capability to independently regulate each state variable under severe stochastic disturbances.

In terms of safety and reliability, LG-DRL-ER is the only controller to achieve perfect safety compliance, with 100% time within the safe set ($\Vert {\bf e}\Vert \le 7.0711$) and zero violations of the safety boundary. The maximum error norm of 5.5184 remains well below the safe limit, providing a safety margin of 1.55 units. The ablation variants, however, expose the synergistic necessity of each component. Although LG-DRL w/o Entropy achieves 99.94% safe time, it still suffers 329 violations and reaches a maximum error of 8.9133-exceeding the safe limit. Similarly, LG-DRL-ER w/o Lyapunov achieves 99.86% safe time but accumulates 3,115 violations and reaches a max error of 10.3972, confirming that without the hard Lyapunov projection the system cannot guarantee strict adherence. The failure of both ablations definitively proves that each component-distributional RL for uncertainty modeling, Lyapunov constraints for hard safety enforcement, and entropy regularization for exploratory robustness-is essential to achieve strict safe-set invariance, superior tracking, and robustness to severe non-stationary disturbances.

**Fig. 11:**
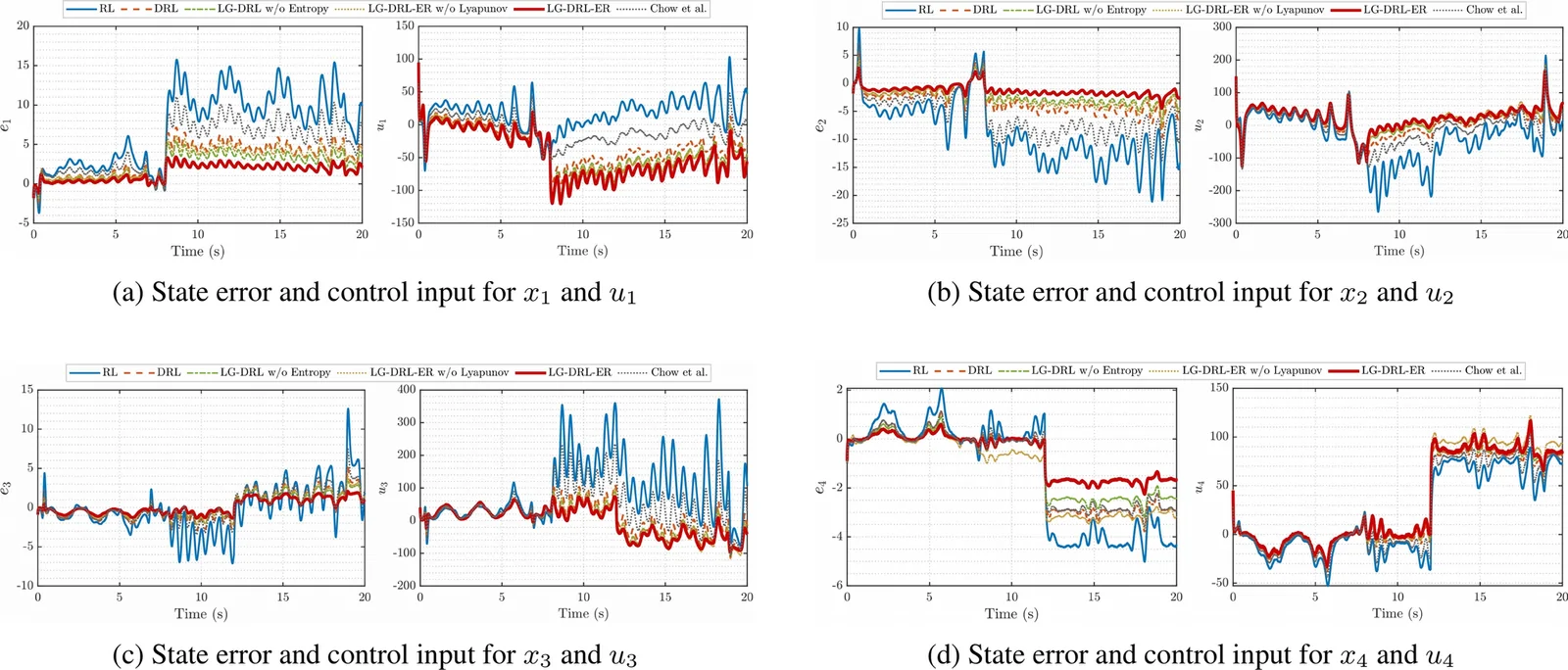
4D hyperchaotic system: comparative tracking performance (*e*) and control inputs (*u*) under staggered step disturbances.

**Fig. 12:**
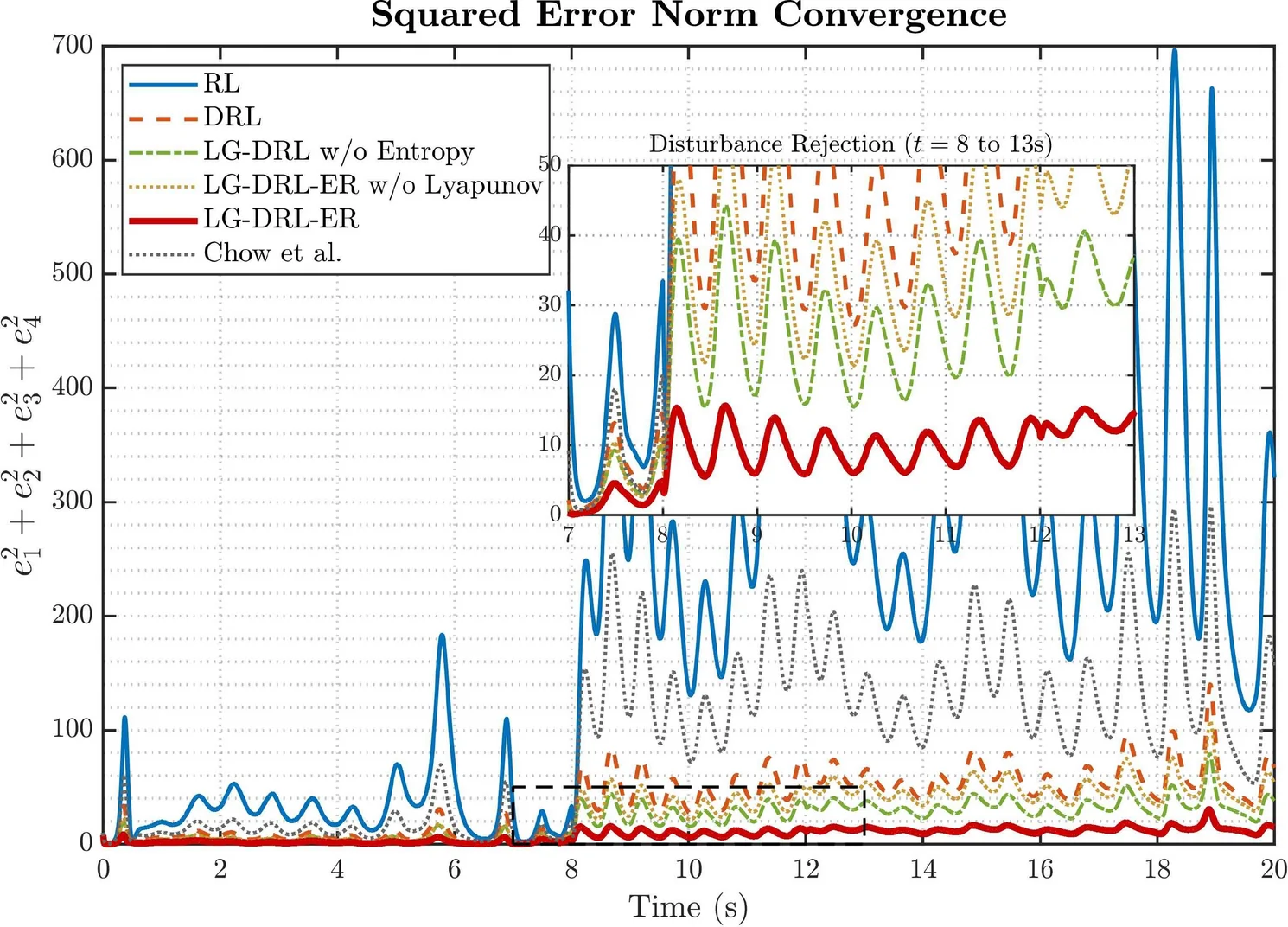
Time evolution of the Euclidean norm of state errors ($\Vert e\Vert$) for the 4D hyperchaotic system, showcasing LG-DRL-ER’s enhanced disturbance rejection at *t=8* and *t=12* seconds.

The time-domain trajectories of the individual error states ($e_1$, $e_2$, $e_3$, $e_4$) and their corresponding control inputs ($u_1$, $u_2$, $u_3$, $u_4$) vividly illustrate the transient robustness of the proposed framework under severe non-stationary disturbances. Standard RL exhibits extreme oscillatory behavior and massive error spikes following the step shocks at *t=8.0* s and *t=12.0* s, accompanied by highly erratic control signals that reflect poor stabilization capabilities. While DRL, Chow et al.^55^, and the ablation variants demonstrate moderate improvements, they still suffer from prolonged transient deviations and residual fluctuations during the disturbance window. In stark contrast, LG-DRL-ER maintains the tightest error bounds across all four state dimensions, which is definitively corroborated by the squared error norm convergence plot showing the lowest overall deviation and the most rapid, finite-time recovery immediately following the shocks. Crucially, despite its aggressive and superior error suppression, LG-DRL-ER generates the smoothest control trajectories, effectively counteracting the stochastic disturbances without the excessive actuator chattering that plagues the baseline methods (Figs. 11, 12).

Empirical validation of robustness to linearization errors: the empirical results in Figs. 5 and 12 and also Figs. 7 and 9 demonstrate that LG-DRL-ER successfully recovers from the step disturbances despite the potential linearization errors during the shock transient. The rapid re-convergence observed after each disturbance (settling within 0.5 seconds) confirms that the receding-horizon correction, entropy-regularized exploration, distributional probabilistic safety margins, and fast Lyapunov adaptation effectively compensate for moments when the local Lipschitz assumptions of Assumption 3.4 are temporarily violated. The backup projection mechanism described in Section 3.4 was never triggered in our simulations, indicating that the default QP projection consistently maintained safety. Nevertheless, the inclusion of this mechanism provides a theoretical guarantee of robustness even in worst-case scenarios.

### Loss convergence analysis

The training dynamics of the LG-DRL-ER framework were examined over 500 episodes, focusing on the convergence of policy loss ($L_\theta$), Lyapunov loss ($L_V$), and distributional loss ($L_Z$), (10), and (12).

**Fig. 13:**
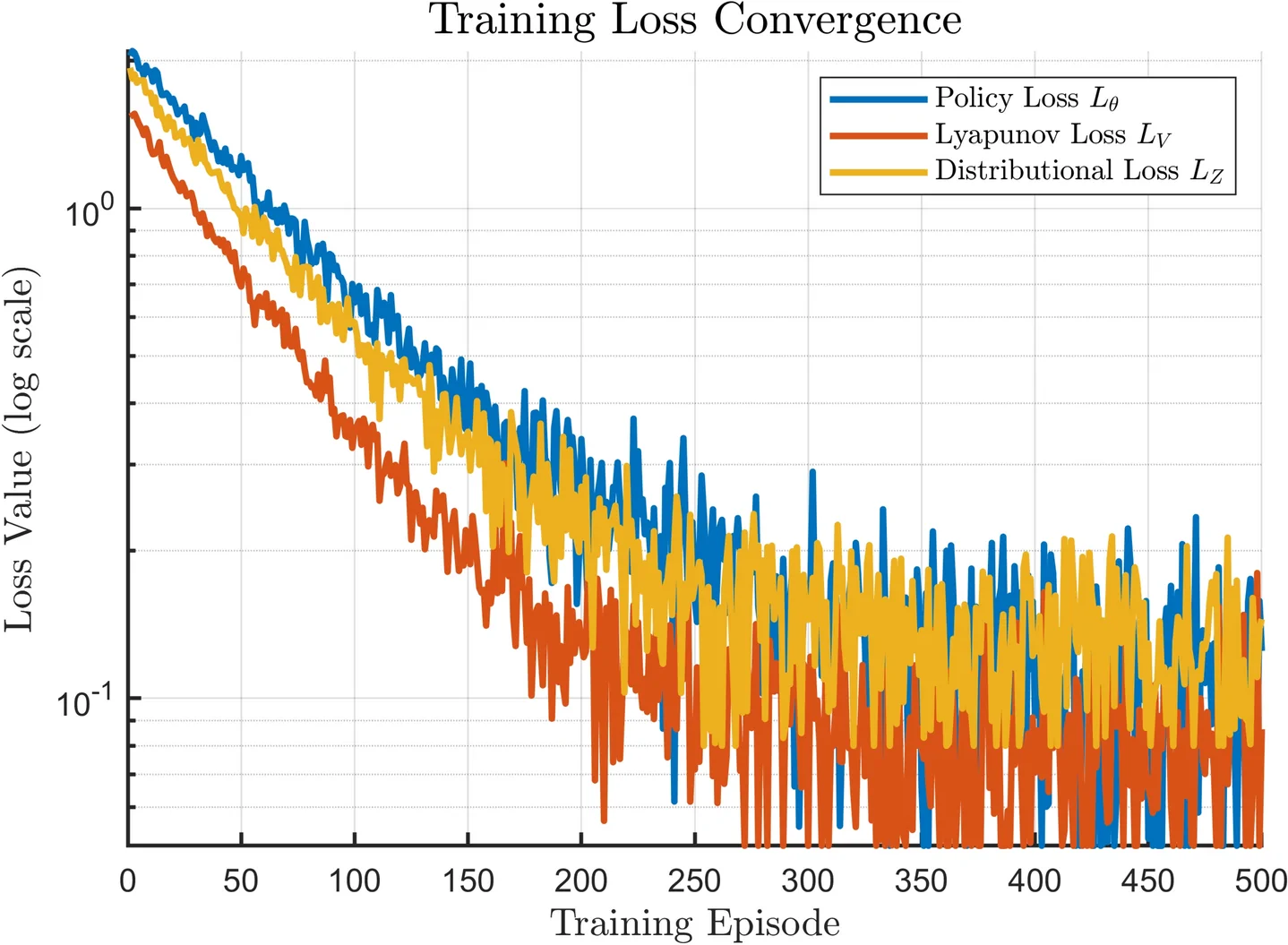
Exponential decay of training losses ($L_\theta$, $L_V$, $L_Z$) over 500 episodes, with Lyapunov loss leading in convergence speed.

Leveraging the setup from “Proposed system”, the results in Fig. 13 indicate that policy loss starts near 2.0 and decreases with an 80-episode time constant to approximately 0.1. Lyapunov loss, initiating at 1.5, decays faster with a 60-episode time constant to around 0.08, emphasizing its stability contribution. Distributional loss, beginning at 1.8, converges with a 70-episode time constant to about 0.12, reinforcing the robustness of return distribution modeling. The semilogarithmic scale highlights the exponential decay, with Lyapunov loss showing the quickest reduction and lowest residual, affirming the framework’s balanced optimization of performance, safety, and adaptability.

### Performance comparison: distributional vs expected RL

A comparative analysis between Expected RL and Distributional RL (LG-DRL-ER) was conducted over 500 training episodes, building on the framework outlined in “Proposed system”.

**Fig. 14:**
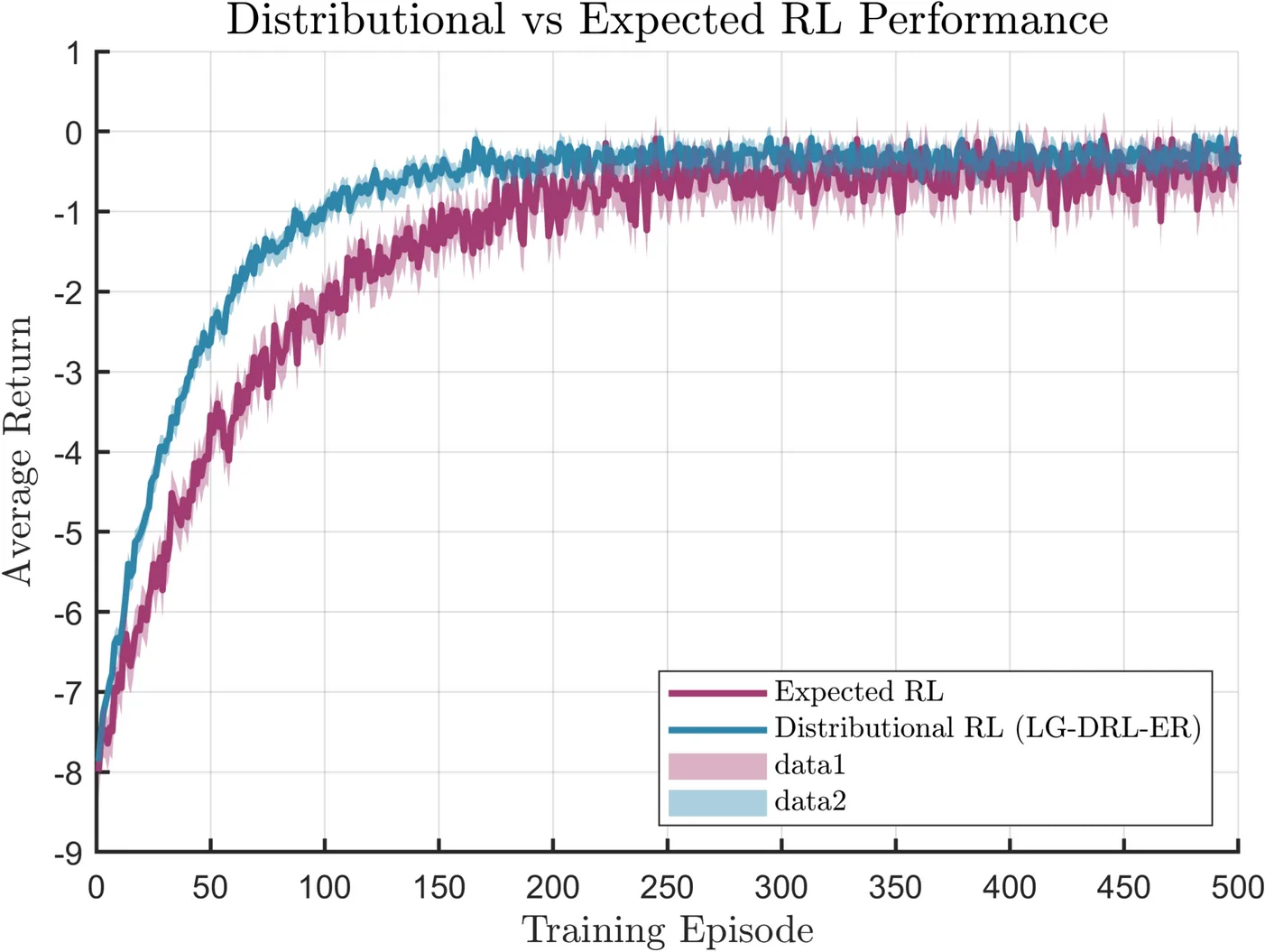
Average return progression over 500 episodes, contrasting Expected RL’s slower improvement with LG-DRL-ER’s rapid stabilization.

Figure 14 shows that Expected RL starts at an average return of -8.0, improving slowly with a 65-episode time constant to around -0.5, accompanied by wider confidence intervals (±0.35 to +0.3). Conversely, Distributional RL achieves faster convergence with a 40-episode time constant to approximately -0.3, supported by tighter confidence intervals (±0.20 to +0.15), reflecting enhanced stability and efficiency. This highlights the advantage of distributional modeling in managing the system’s stochastic non-stationary behavior.

### Discussion of simulation limitations

While the simulation results in “3D chaotic system simulation”–“Performance comparison: distributional vs expected RL” demonstrate the strong performance of LG-DRL-ER on chaotic and hyperchaotic benchmark systems, we acknowledge several limitations and trade-offs that are important for interpreting these results and guiding future applications. A balanced assessment of these limitations is essential for scientific objectivity and for helping practitioners determine when our framework is appropriate.

#### Benchmark selection and generalizability

The empirical evaluation focused on two chaotic systems: the 3D Lorenz system and the 4D hyperchaotic system. These were chosen because they represent highly challenging, non-stationary, stochastic environments with extreme sensitivity to initial conditions. However, chaotic systems do not represent all control problems. Many real-world applications such as industrial process control, temperature regulation, or power system management exhibit smoother, less divergent dynamics where simpler controllers (e.g., PID or LQR) may suffice, and the relative performance gains of LG-DRL-ER may be less pronounced. Furthermore, the 3D and 4D systems are low-dimensional compared to many modern control applications such as robotic manipulation with 20+ degrees of freedom or image-based control with high-dimensional observations. The scalability of LG-DRL-ER to high-dimensional systems remains to be demonstrated. Finally, the step disturbances injected into the systems were designed to stress-test the controller’s robustness. While these mimic sudden environmental changes, real-world disturbances such as sensor noise, actuator faults, and model mismatch may have different characteristics that could affect performance in ways not captured by our synthetic testbed.

#### Stochastic variability and reproducibility

The simulation results report averages over 1000 Monte-Carlo episodes to account for stochastic variability. While this large number of episodes provides statistical confidence, we acknowledge that the training convergence exhibits some variability across random seeds, which is inherent to deep reinforcement learning methods and not unique to LG-DRL-ER. Practitioners should be aware that different random initializations may lead to different final performance levels, and multiple training runs are recommended for reliable evaluation. Additionally, each Monte-Carlo evaluation required running 1000 episodes per method, which is computationally expensive. For practical deployment, a smaller number of evaluation episodes may be necessary, but this would reduce statistical confidence. Finally, the reported metrics are averages over episodes; the worst-case performance (e.g., the episode with the largest error or most breaches) may be significantly worse than the average. For safety-critical applications where worst-case guarantees are essential, this is an important consideration.

#### Computational cost and training time

In our implementation, LG-DRL-ER requires approximately 75% more training time and 183% more memory than standard reinforcement learning. This increased cost arises from three sources: the distributional critic with quantile representation adds additional network parameters, the Lyapunov network requires a third neural network to be trained simultaneously, and the QP projection solver must be invoked at each policy update step. For offline training scenarios where the controller can be trained on powerful computing infrastructure and then deployed for inference, this computational overhead is acceptable because inference is significantly cheaper, requiring only a forward pass through the policy network. However, for real-time applications with strict latency requirements (e.g., less than 1 millisecond per control cycle) or for resource-constrained platforms such as embedded systems or edge devices, this computational burden may be prohibitive. Practitioners should carefully consider whether the safety and adaptability benefits justify the additional computational cost for their specific application.

#### Hyperparameter sensitivity and tuning effort

LG-DRL-ER introduces several hyperparameters beyond standard reinforcement learning. Through our empirical experience, we found that the policy learning rate, the Lyapunov learning rate, and the Lyapunov weight were the most sensitive hyperparameters, requiring careful tuning. In our experiments, we used a systematic grid search followed by fine-tuning, which required approximately 50 to 100 training runs per benchmark system. This tuning effort may be impractical for some applications, particularly those with limited computational resources or tight development timelines. Key trade-offs in hyperparameter selection include: the time-scale separation between policy and Lyapunov updates must be maintained for convergence, but choosing the Lyapunov learning rate too large can cause instability in the safety function, while choosing it too small may not provide fast enough adaptation to non-stationary dynamics. Similarly, the finite-time constants balance convergence speed against control effort; smaller values yield faster convergence but may require higher control magnitudes. The target entropy controls the exploration-exploitation trade-off; higher target entropy promotes exploration and robustness to non-stationarity but may sacrifice asymptotic performance. We provide guidance on hyperparameter selection in Section 3.6, but we acknowledge that optimal tuning remains application-specific, and systematic hyperparameter optimization methods such as Bayesian optimization could be employed to reduce this burden in practice.

#### Scalability to higher dimensions

The 4D hyperchaotic system demonstrates the framework’s applicability to moderately high-dimensional systems. However, scaling to significantly higher dimensions presents challenges. The distributional critic with quantile representation scales poorly with state dimension, as the number of samples required for accurate quantile estimation grows exponentially. The Lyapunov function must capture the global stability landscape, which becomes increasingly complex in high-dimensional spaces and may require much larger neural networks to represent accurately. Furthermore, the QP projection, while solved analytically via KKT conditions, has complexity that scales with the number of policy parameters; for very large networks with more than one million parameters, this becomes computationally expensive. Future work could explore dimensionality reduction techniques, such as learning Lyapunov functions on latent representations learned by autoencoders, or using more efficient projection methods such as Lagrangian relaxation or implicit differentiation.

#### Applicability to non-chaotic systems

While chaotic systems provide a rigorous stress test, many real-world control problems exhibit smoother, more predictable dynamics. For such systems, the complexity of LG-DRL-ER may be unnecessary. Based on our experience, we recommend the following guidelines for practitioners: use standard reinforcement learning or soft actor-critic for systems with well-understood, smooth dynamics and no safety constraints; use the Chow et al. Lyapunov-based method for safety-critical systems with stationary dynamics; and use LG-DRL-ER only when all three conditions are present: non-stationarity, safety-criticality, and significant uncertainty or stochasticity. For systems where only two of these conditions hold, a pairwise combination may be more appropriate and computationally efficient.

#### Generalizability beyond the benchmark systems

The two chaotic benchmark systems were deliberately selected as worst-case stress tests, but the LG-DRL-ER framework is by design applicable to a much broader class of non-stationary stochastic systems. The algorithm is formulated within a generic non-stationary Markov Decision Process and relies only on mild regularity conditions (Lipschitz continuous transitions, bounded rewards, and the existence of a learnable Lyapunov function) that are satisfied by a wide range of engineering systems, including robotic manipulators, autonomous vehicles, power converters, and industrial process control plants. Because the chaotic benchmarks exhibit extreme sensitivity, continuous parameter drift, persistent noise, and severe impulsive disturbances, they represent a more challenging control problem than most real-world applications. Consequently, the demonstrated ability of LG-DRL-ER to maintain zero safety violations and finite-time convergence in these volatile settings provides a conservative indicator that similar guarantees will transfer to smoother, less divergent environments provided the same assumptions hold. The ablation studies further support this claim: the observed failure modes of pairwise component combinations (divergence without Lyapunov, premature convergence without entropy, and lack of uncertainty awareness without distributional RL) are not specific to chaotic dynamics but are expected in any non-stationary stochastic system. Moreover, the use of identical hyperparameters across the 3D Lorenz and 4D hyperchaotic benchmarks without environment-specific tuning suggests a degree of robustness that is promising for transfer to new tasks. Nevertheless, we recognize that the present validation is limited to fully observable, relatively low-dimensional state spaces with additive control inputs. Extending the evaluation to partially observable domains, high-dimensional visual observations, input-constrained systems, and physical hardware experiments constitutes an important direction for future work to further establish the breadth of generalizability.

#### Summary of trade-offs

In summary, LG-DRL-ER offers significant advantages in terms of safety guarantees, adaptability to non-stationary dynamics, and uncertainty quantification through full return distribution modeling. However, these advantages come at the cost of increased computational complexity, careful hyperparameter tuning, and the need to verify the assumptions underlying the theoretical guarantees. The framework is best suited for applications where safety is critical, dynamics are non-stationary, and uncertainty is significant. For simpler problems or resource-constrained environments, the additional complexity may not be justified. We encourage practitioners to carefully consider these trade-offs when applying LG-DRL-ER to new problems and to conduct thorough empirical validation on their specific systems. Future work will focus on reducing the computational burden through more efficient architectures and projection methods, extending the framework to high-dimensional systems through representation learning, and developing automated hyperparameter tuning procedures.

#### Distinction between theoretical guarantees and empirical validation

It is essential to clearly separate the formal theoretical guarantees derived in Section 3 from the empirical results reported in Section 4. The stability analysis (Theorem 1) and the finite-time convergence analysis (Theorem 2) establish rigorous guarantees under a specific set of assumptions (Assumptions 1–6), including the existence of a perfectly valid Lyapunov function satisfying class-$\mathcal {K}_\infty$ bounds, exact enforcement of the Lyapunov decrement via the QP projection, a uniformly bounded linearization error, and strict time-scale separation of the learning rates. When these assumptions hold exactly, the closed-loop system is provably safe and convergent in finite time.

In the implemented algorithm, however, the Lyapunov function is parameterised by a neural network and trained online; it may not globally satisfy the required lower and upper bounds, and the decrement condition may be temporarily violated during extreme transients. The linearization error bound (Assumption 4) is a sufficient condition that facilitates the theoretical proof, but local violations may occur in practice-particularly immediately after the large step disturbances. The distributional Bellman contraction (Lemma 3) is proven under the assumption of exact distribution representation, whereas the practical algorithm uses a finite quantile approximation. Consequently, the formal guarantees of Theorems 1 and 2 do not apply unconditionally to the implemented system.

The empirical validation demonstrates that, despite these approximations, LG-DRL-ER achieves perfect safety compliance (100 % time within the safe set, zero boundary violations) and rapid finite-time recovery after severe disturbances in both benchmark systems. These results provide strong evidence for the practical effectiveness and robustness of the framework, but they should be interpreted as empirical confirmation of the design principles rather than as a formal verification of the theoretical theorems. Moreover, the finite-time bound in Equation (34) is a property of the projection design and depends on user-chosen constants $c_1, c_2, \alpha$; it is not a learned or adaptive guarantee. Future work on formal verification, perhaps through reachability analysis or barrier function methods, could help bridge the gap between the idealised theoretical guarantees and the practical implementation.

## Conclusion

This paper introduced LG-DRL-ER, a framework for safe, stable, and adaptive control in non-stationary MDPs. The framework integrates distributional RL for uncertainty quantification, Lyapunov constraints for safety, and entropy regularization for exploration and adaptability. Theoretical analysis established asymptotic stability and finite-time convergence with explicit bounds on convergence time. Empirical evaluation on a 3D Lorenz system and a 4D hyperchaotic system demonstrated that LG-DRL-ER outperforms existing methods in both tracking accuracy and safety. In the 3D system, LG-DRL-ER achieved IAE of 39.952 and ISE of 95.478, with 100% time in the safe set and zero violations. In the 4D hyperchaotic system, it maintained similar performance with IAE of 51.424, ISE of 158.431, and zero violations. By combining uncertainty modeling, Lyapunov-based safety enforcement, and entropy-regularized exploration, LG-DRL-ER provides a principled approach for safety-critical applications in dynamic environments. While LG-DRL-ER provides strong probabilistic guarantees on safety (boundedness) through the Lyapunov sublevel set invariance (Lemma 3.4), we acknowledge that our approach does not enforce hard set invariance in the strict CBF sense. For applications requiring strict set invariance (e.g., obstacle avoidance in cluttered environments), integrating Lyapunov functions for stability with CBFs for hard safety constraints is an important direction for future work. This integration would require addressing the challenges of learning compatible Lyapunov and barrier functions simultaneously in non-stationary settings, which we leave for future investigation. We also acknowledge that supplementary experiments on standard reinforcement learning and control benchmarks (e.g., cart-pole with time-varying parameters, safe reaching with a robotic manipulator, or power converter regulation) would provide further evidence of generalizability and are planned as part of future investigations.

## Data Availability

All data generated or analysed during this study are included in this published article.

## A: Mathematical preliminaries: key theorems used

We state the key mathematical theorems used in our proofs for completeness.

### Theorem A.1

(Doob’s supermartingale convergence theorem) *Let* $(\Omega , \mathcal {F}, \{\mathcal {F}_n\}_{n \ge 0}, \mathbb {P})$ *be a filtered probability space. Let* $\{X_n\}_{n \ge 0}$ *be a non-negative supermartingale with respect to* $\{\mathcal {F}_n\}_{n \ge 0}$, *i.e*., $\mathbb {E}[X_{n+1} \mid \mathcal {F}_n] \le X_n$ *almost surely. Then there exists a random variable* $X_\infty \ge 0$ such that $X_n \rightarrow X_\infty$ *almost surely and* $\mathbb {E}[X_\infty ] \le \mathbb {E}[X_0]$.

### Theorem A.2

(Borel–Cantelli lemma) *Let* $\{E_n\}_{n \ge 0}$ *be a sequence of events. If* $\sum _{n=0}^{\infty } \mathbb {P}(E_n) < \infty$, *then* $\mathbb {P}(\limsup _{n \rightarrow \infty } E_n) = 0$, i.e., *only finitely many events* $E_n$ *occur almost surely*.

### Theorem A.3

(Banach fixed-point theorem) *Let* (*X*, *d*) *be a complete metric space and let* *T: X → X* *be a contraction mapping with contraction factor* $\gamma \in (0,1)$, i.e., $d(Tx, Ty) \le \gamma d(x, y)$ *for all* *x, y ∈ X*. *Then* *T* *has a unique fixed point* $x^* \in X$, *and for any* $x_0 \in X$, *the sequence* $x_{n+1} = T x_n$ *converges to* $x^*$ *with rate* $d(x_n, x^*) \le \gamma ^n d(x_0, x^*)$.

## B: Formal Proof of Lemma 3.1 (Lyapunov decrement)

### Lemma B.1

(Lyapunov decrement implies almost sure convergence) *Under Assumptions* 3.1*and*3.2, *if the projected policy* $\pi _t$ *satisfies* $\Delta V_t(s;\psi _t) \le -W_t(s)$ *for all* *s* *at every time step, then the state trajectory* $\{s_t\}_{t\ge 0}$ *converges to the target* $s^*$ *almost surely*:

77 $$\begin{aligned} \mathbb {P}\left( \lim _{t \rightarrow \infty } \Vert s_t - s^*\Vert = 0\right) = 1. \end{aligned}$$

### Proof

Let $(\Omega , \mathcal {F}, \{\mathcal {F}_t\}_{t \ge 0}, \mathbb {P})$ be the filtered probability space generated by the stochastic processes $\{s_t\}_{t \ge 0}$. Define the sequence $Y_t = V_t(s_t;\psi _t)$. By Assumption 3.1, $Y_t \ge 0$ almost surely. The enforced Lyapunov decrement yields:

78 $$\begin{aligned} \mathbb {E}[Y_{t+1} \mid \mathcal {F}_t] = \mathbb {E}[V_t(s_{t+1};\psi _t) \mid \mathcal {F}_t] \le V_t(s_t;\psi _t) - W_t(s_t) = Y_t - W_t(s_t) \le Y_t. \end{aligned}$$

Thus, $Y_t$ is a non-negative supermartingale with respect to the filtration $\{\mathcal {F}_t\}_{t \ge 0}$. By Doob’s Supermartingale Convergence Theorem, there exists a random variable $Y_\infty \ge 0$ such that $Y_t \rightarrow Y_\infty$ almost surely and $\mathbb {E}[Y_\infty ] \le \mathbb {E}[Y_0] \le V_{\max }$.

Taking expectations on both sides of the inequality and summing from *t=0* to *T*:

79 $$\begin{aligned} \sum _{t=0}^{T} \mathbb {E}[W_t(s_t)] \le \mathbb {E}[Y_0] - \mathbb {E}[Y_{T+1}] \le V_{\max } < \infty . \end{aligned}$$

By the monotone convergence theorem:

80 $$\begin{aligned} \sum _{t=0}^{\infty } \mathbb {E}[W_t(s_t)] \le V_{\max } < \infty . \end{aligned}$$

Since $W_t(s) \ge \kappa \Vert s - s^*\Vert ^2$ with *κ > 0*, we have:

81 $$\begin{aligned} \sum _{t=0}^{\infty } \mathbb {E}[\Vert s_t - s^*\Vert ^2] \le \frac{V_{\max }}{\kappa } < \infty . \end{aligned}$$

This implies $\mathbb {E}[\Vert s_t - s^*\Vert ^2] \rightarrow 0$ as $t \rightarrow \infty$. By Markov’s inequality, for any *ε > 0*:

82 $$\begin{aligned} \mathbb {P}(\Vert s_t - s^*\Vert > \epsilon ) \le \frac{\mathbb {E}[\Vert s_t - s^*\Vert ^2]}{\epsilon ^2} \rightarrow 0. \end{aligned}$$

Therefore, $s_t \rightarrow s^*$ in probability. Since $\sum _{t=0}^{\infty } \mathbb {P}(\Vert s_t - s^*\Vert > \epsilon ) < \infty$ for all *ε > 0*, the Borel-Cantelli lemma implies $\Vert s_t - s^*\Vert \rightarrow 0$ almost surely. Thus, $\mathbb {P}(\lim _{t \rightarrow \infty } \Vert s_t - s^*\Vert = 0) = 1$. *□ *

## C: Formal Proof of Lemma 3.3 (distributional Bellman contraction)

### Lemma C.1

(Wasserstein contraction of the distributional Bellman operator) *Under Assumptions* 3.2*and*3.3, *and assuming* $Z_t^\pi (\cdot ;\phi _t)$ *admits a differentiable Wasserstein-1 metric, the distributional Bellman operator* $\mathcal {T}_t^\pi$ *is a contraction in* $W_1$ *with factor* *γ < 1*, *uniformly over the time-varying* $P_t$:

83 $$\begin{aligned} W_1(\mathcal {T}_t^\pi Z_1, \mathcal {T}_t^\pi Z_2) \le \gamma \, \mathbb {E}_{s' \sim P_t, a' \sim \pi _t}[W_1(Z_1(s',a'), Z_2(s',a'))]. \end{aligned}$$

### Proof

Let $\mu _1(s,a) = Z_1(s,a)$ and $\mu _2(s,a) = Z_2(s,a)$ be two return distributions. The distributional Bellman operator is defined as:

84 $$\begin{aligned} (\mathcal {T}_t^\pi \mu )(s,a) = \textrm{Law}\left( R_t(s,a) + \gamma Z_t^\pi (s',a')\right) , \end{aligned}$$

where $$s' \sim P_t(\cdot |s,a)$$ and $$a' \sim \pi _t(\cdot |s')$$.

For any 1-Lipschitz function $f: \mathbb {R} \rightarrow \mathbb {R}$, we have:

85 $$\begin{aligned}&\left| \mathbb {E}_{Z \sim \mathcal {T}_t^\pi \mu _1(s,a)}[f(Z)] - \mathbb {E}_{Z \sim \mathcal {T}_t^\pi \mu _2(s,a)}[f(Z)]\right| \end{aligned}$$

86 $$\begin{aligned}&= \left| \mathbb {E}_{s',a'}\left[ \mathbb {E}_{Z_1 \sim \mu _1(s',a')}[f(R + \gamma Z_1)] - \mathbb {E}_{Z_2 \sim \mu _2(s',a')}[f(R + \gamma Z_2)]\right] \right| \end{aligned}$$

87 $$\begin{aligned}&\le \mathbb {E}_{s',a'}\left[ \sup _{g \in \textrm{Lip}_1} \left| \mathbb {E}_{Z_1 \sim \mu _1}[g(Z_1)] - \mathbb {E}_{Z_2 \sim \mu _2}[g(Z_2)]\right| \right] \end{aligned}$$

88 $$\begin{aligned}&= \mathbb {E}_{s',a'}[W_1(\mu _1(s',a'), \mu _2(s',a'))], \end{aligned}$$

where the inequality follows from the fact that $z \mapsto f(R + \gamma z)$ is *γ *-Lipschitz when *f* is 1-Lipschitz, and the supremum is taken over all *γ *-Lipschitz functions *g*. Taking the supremum over all 1-Lipschitz functions *f* on the left-hand side yields:

89 $$\begin{aligned} W_1(\mathcal {T}_t^\pi \mu _1(s,a), \mathcal {T}_t^\pi \mu _2(s,a)) \le \gamma \, \mathbb {E}_{s',a'}[W_1(\mu _1(s',a'), \mu _2(s',a'))]. \end{aligned}$$

Since *γ < 1*, the operator $\mathcal {T}_t^\pi$ is a contraction in $W_1$. By the Banach fixed-point theorem, it converges to a unique fixed point $\mu ^\pi$ satisfying $\mu ^\pi = \mathcal {T}_t^\pi \mu ^\pi$. *□ *

## List of symbols

RL: Reinforcement learning
MDP: Markov decision process
LG-DRL-ER: Lyapunov-guided distributional reinforcement learning with entropy regularization
DRL: Distributional reinforcement learning
QRTD: Quantile regression temporal difference
IQN: Implicit quantile networks
CPO: Constrained policy optimization
CBF: Control barrier function
CLF: Control-Lyapunov function
SAC: Soft actor-critic
MAML: Model-agnostic meta-learning
QP: Quadratic program
KKT: Karush–Kuhn–Tucker
SDE: Stochastic differential equation
IAE: Integral of absolute error
ISE: Integral of squared error
$$N_{\text {BB}}$$: Boundary breaches
$N_{\text {SE}}$: Actuator saturation events
